# IFIT1 + neutrophil is a causative factor of immunosuppressive features of poorly cohesive carcinoma (PCC)

**DOI:** 10.1186/s12967-024-05389-z

**Published:** 2024-06-19

**Authors:** Yuan-jie Liu, Jie-pin Li, Mei Han, Jing-xiao Li, Qian-wen Ye, Si-tian Lin, Jin-yong Zhou, Shen-lin Liu, Xi Zou

**Affiliations:** 1 Department of Oncology, Affiliated Hospital of Nanjing University of Chinese Medicine, Jiangsu Province Hospital of Chinese Medicine, Nanjing, 210029 Jiangsu China; 2 Department of Pathology, Affiliated Hospital of Nanjing University of Chinese Medicine, Jiangsu Province Hospital of Chinese Medicine, Nanjing, 210029 Jiangsu China; 3 No. 1 Clinical Medical College, Nanjing University of Chinese Medicine, Nanjing, 210023 Jiangsu China; 4 Jiangsu Collaborative Innovation Center of Traditional Chinese Medicine in Prevention and Treatment of Tumor, Nanjing, 210029 Jiangsu China; 5 Key Laboratory of Tumor System Biology of Traditional Chinese Medicine, Nanjing, 210029 Jiangsu China; 6 Central Laboratory, Affiliated Hospital of Nanjing University of Chinese Medicine, Jiangsu Province Hospital of Chinese Medicine, Nanjing, 210029 Jiangsu China

**Keywords:** Poorly cohesive carcinoma (PCC), Neutrophil, Tumor microenvironment (TME), IFIT1, CD274 (PDL1)

## Abstract

The importance of the immune microenvironment in poorly cohesive carcinoma (PCC) has been highlighted due to its limited response rate to conventional therapy and emerging treatment resistance. A combination of clinical cohorts, bioinformatics analyses, and functional/molecular experiments revealed that high infiltration of Interferon Induced Protein with Tetratricopeptide Repeats 1 (IFIT1) + tumor-associated neutrophils (TANs) is a distinguishing feature of PCC patients. Upregulation of IFIT1 + TANs promote migration and invasion of gastric cancer (GC) cell lines (MKN45 and MKN74) and stimulates the growth of cell-derived xenograft models. Besides, by promoting macrophage secreted phosphoprotein 1 (SPP1) expression and facilitating cancer-associated fibroblast and endothelial cell recruitment and activation through TANs, IFIT1 promotes a mesenchymal phenotype, which is associated with a poor prognosis. Importantly, compared to non-PCC (NPCC), PCC tumors is more immunosuppressive. Mechanistically, IFIT1 can be stimulated by IFN-γ and contributes to the expression of Programmed Cell Death 1 Ligand (PDL1) in TANs. We demonstrated in mouse models that IFIT1 + PDL1 + TANs can induce acquired resistance to anti-PD-1 immunotherapy, which may be responsible for the difficulty of PCC patients to benefit from immunotherapy. This work highlights the role of IFIT1 + TANs in mediating the remodeling of the tumor immune microenvironment and immunotherapeutic resistance and introduces IFIT1 + TANs as a promising target for precision therapy of PCC.

**Supplementary Information:**

The online version contains supplementary material available at 10.1186/s12967-024-05389-z.

## Introduction

As the fifth most commonly diagnosed cancer worldwide, Gastric cancer (GC) contributes to the fourth highest cancer-related mortality rate [1]. GC incidence shows strong geographic variation, with the highest prevalence in East Asia, placing a heavy economic burden on local health care systems [2]. Although the overall incidence of GC has declined over the past few decades thanks to advances in screening tools for early-stage cancer, the relative incidence of Poorly cohesive carcinoma (PCC) has been steadily increasing [3, 4]. Compared with other histologic subtypes of GC, PCC occurs in young adults and patients with PCC have a low survival rate [5, 6]. As defined by the World Health Organization (WHO), PCC consists of isolated or small clusters of tumor cells, tends to exhibit greater aggressiveness, and responds poorly to currently available therapies [7].

Although histopathologic classification has excellent clinical applicability and facilitates clinical decision-making by medical practitioners, molecular typing demonstrates the potential to develop subtype-specific precision therapies [8]. In recent years, a number of studies have developed various molecular-based typing systems in attempts to link the molecular features of GC with clinical and histologic features [9]. Sang Cheul Oh et al. identified two distinct molecular subtypes of GC: Mesenchymal phenotype (MP) and Epithelial phenotype (EP) based on The Cancer Genome Atlas (TCGA) and Asian Cancer Research Group (ACRG) programs [10]. Clinically, the MP subtype exhibited significantly poorer survival and resistance to treatment [11]. Importantly, up to 61.9% of examples of MP subtype cases were of the diffuse histological type and contained a higher non-tumor component [10]. In addition, a clinical cohort-based single-cell sequencing analysis revealed that poorly differentiated (diffuse) GCs were characterized by significant immunosuppression and a high degree of Epithelial mesenchymal transition (EMT) gene activation [12]. According to the most recent histological definitions, a significant proportion of examples of diffuse GC are PCC tumors, the latter being further classified as Signet Cell Carcinoma (SRC), Combined, and Not-otherwise-specified (NOS) PCC [13, 14]. There are few detailed studies on the molecular or biological mechanisms of PCC. A deeper understanding of the unique phenotype of PCC may provide a useful basis for exploring new therapeutic strategies for patients with PCC.

Mature neutrophils recruited to the tumor region are educated by tumor cells and acquire the phenotype of highly activated neutrophils, leading to the formation of Tumor-associated neutrophils (TANs) with immunosuppressive functions [15]. During this differentiation process, immature neutrophils with a high degree of plasticity respond to high levels of IFN-γ and Granulocyte–macrophage colony-stimulating factor (GM-CSF) in tumor tissues by transforming into Programmed death-ligand 1 (PD-L1) heterotrimeric neutrophils, which severely inhibit T cell-mediated tumor immunity [16]. Interestingly, under low-dose IFN-γ stimulation or under physiological conditions, neutrophils tend to exhibit Antigen-presenting cell (APC) characteristics, which trigger and enhance anti-tumor immune responses [17]. Broadly defined, neutrophils infiltrating solid tumors can be roughly divided into the TAN1 subtype, which possesses cytotoxic effects, and the TAN2 subtype, which supports tumor progression [18]. However, the landscape of tumor immune infiltration is complex and dynamic and is influenced by intra- and inter-tumor heterogeneity. Neutrophils reprogrammed in the context of cancer are an important component of the Tumor microenvironment (TME) and play a key role in tumor progression [19].

We previously identified an EMT signaling axis [Ubiquitin Specific Peptidase 51 (USP51)-Zinc Finger E-Box Binding Homeobox 1 (ZEB1)-Actin Alpha 2 (ACTA2)] in PCC, reinforcing the link between PCC and the mesenchymal phenotype [20]. In this study, by integrating a GC patient cohort, public datasets, and in vitro and in vivo models, we found that neutrophil-expressed Interferon Induced Protein with Tetratricopeptide Repeats 1 (IFIT1) promoted EMT in GC, particularly in PCC-GC, and promoted mesenchymal cell recruitment through multiple signals, which subsequently facilitated an exhausted phenotype of T cells and induced resistance to immunotherapy. In addition to revealing the role of IFIT1 in the mesenchymal phenotype of PCC, our work provides new insights into the role of IFN-γ/PDL1 in immunomodulation.

## Materials and methods

A comprehensive inventory of chemical compounds and antibodies employed in this study can be found in the Supplementary Material (Table S1). The concentrations of antibodies used were determined based on the recommendations of the respective manufacturer or previous research findings. The Supplementary Materials offer comprehensive information on data analysis, criteria for participant inclusion/exclusion, as well as supplementary details, tables, and figures.

### Transmission electron microscopy (TEM)

The tissue and cell TEM was carried out per the manufacturer’s protocol. Specimens were dyed with 0.3% lead citrate and photographed via an electron microscope (Hitachi, Tokyo, Japan; 2500 × or 30000 × Magnification).

### Immunohistochemistry (IHC) staining

Initially, the tissues were embedded in paraffin, followed by cutting the paraffin-embedded tissue sections and mounting them onto slides. IHC was conducted in accordance with a standardized protocol [21]. The calculation of the IHC score, also known as the H-score, was performed based on previously published literature [22]. Two pathologists performed the pathological diagnosis independently.

### Hematoxylin/eosin (HE) staining

The histopathology of GC tissues was assessed using HE staining. GC samples were subjected to dehydration in an ethanol gradient, followed by embedding in paraffin and sectioning into 4 μm pieces subsequent to immersion in a 10% formaldehyde solution. Following deparaffinization, the sections were stained with hematoxylin and eosin, mounted, and subsequently examined under an upright epifluorescent microscope (Nikon, Eclipse Ni-E, Tokyo, Japan).

### Flow cytometry

T cells were evaluated by flow cytometry. Specifically, two subsets were examined: (1) activated T cells (CD3 + , CD8 + , CD69 +), and (2) exhausted T cells (CD3 + , PD-1). Peripheral blood samples were collected in heparin sodium tubes. A 50 μL aliquot of each sample was taken and red blood cells were lysed using 3 times the volume of red blood cell lysis buffer. This process was carried out for 10 min at room temperature. Following a 5-min centrifugation, the cells were washed with 5 mL of PBS, centrifuged again, and resuspended in PBS. The cells were then counted in preparation for subsequent staining and analysis using flow cytometry.

For cell surface staining, the previously mentioned cells were suspended in 50 µL of PBS. They were stained with the different antibody panels in the dark at a temperature of 4 °C for a period of 30 min and washed a second time with PBS + 5% FBS. Then Flow cytometry was performed on the flow cytometer (BD BioSciences, FACS Celesta, Franklin Lakes, NJ, USA) and the data were analyzed by the Kaluza software. All experiments were replicated at least 3 times. The results were expressed as the mean value and standard error of the mean (SEM) if not indicated otherwise.

### Neutrophil chemotaxis assay

Neutrophil chemotaxis was assessed using a fluorescent chemotaxis assay, employing Calcein-labeled human neutrophils and 3-μm transwell filters. The upper compartment of the assay system contained 200 μL of neutrophil suspension (1 × 10^5^ cells), while the bottom chamber received 300 μL of different GC cell supernatants as conditioned medium. Following a 60-min incubation in a 5% CO_2_ incubator, images were captured using a fluorescence microscope (Olympus, BX-63, Tokyo, Japan) and the cell number was counted using ImageJ software.

### Immunofluorescence

Cells were seeded onto glass slides in 24-well culture plates. After indicated treatment, cells were fixed with formaldehyde (4%) and permeabilized with 0.3% Triton X-100. The slides were then washed by PBS and incubated with primary antibodies overnight. Next, the slides were stained with appropriate secondary antibodies and 4, 6-diamidino-2-phenylindole (DAPI). The multicolor immunofluorescence assessment for tumor tissue was based on the tyramide signal amplification (TSA) system. In brief, the sliced tissue specimens were dewaxed, rehydrated, treated for Heating-induced epitope retrieval (HIER) with H_2_O_2_, blocked using 3% Bovine serum albumin (BSA) to inhibit nonspecific interaction, labeled with primary and then with horseradish peroxidase (HRP)-conjugated anti-rabbit secondary antibodies and fluorescent tyramide successively. Then the sections were treated for HIER, BSA blocking, and antibody staining again; lastly, the nuclei were dyed with DAPI, and imaged under fluorescence microscope (Nikon, DS-QilMC, Tokyo, Japan).

### CAFs and endothelial cell migration assay

CAFs (2 × 10^4^/well) or HUVECs (2 × 10^4^/well) were propagated at the 24-well 8 μm Transwell upper chambers with medium (200 μL). Neutrophils (1 × 10^5^/well) seeded into the bottom chamber with medium (500 μL) augmented with 10% FBS after treatment with or without NC/knockdown/overexpression IFIT1. After co-culturing for a duration of 24 h, the upper Transwell chamber was rinsed with PBS. Subsequently, the chambers were treated with a 4% paraformaldehyde solution for a duration of 15 min before being stained with 0.1% crystal violet. The resulting images of the cells that successfully invaded the lower chambers were captured using a phase-contrast microscope (Olympus, CKX 41, Hachioji, Japan), and the cells were quantified using ImageJ.

### In vitro tumor cell invasion assay

GC Cells invasion assay was conducted using Matrigel-coated invasion chambers (24-well 8 μm). GC Cells (2 × 10^4^/well) were resuspended in 200 µL serum-free RPMI-1640 added to the upper compartments of the chambers. CAFs (2 × 10^4^/well) and neutrophils treatment with or without NC/knockdown/overexpression IFIT1 (2 × 10^4^/well) seeded into the bottom chamber with medium (500 μL) augmented with 10% FBS. After being incubated for a duration of 24 h at a temperature of 37 °C, the chamber underwent a gentle washing and swabbing process to eliminate cells that had not successfully traversed the membrane. Subsequently, the cells situated on the lower side of the insert were subjected to fixation using a 4% formaldehyde solution, followed by staining with crystal violet at a concentration of 0.1%. The quantification of migrated cells was accomplished by employing a phase-contrast microscope (Olympus CKX 41, Olympus, Hachioji, Japan).

### Wound healing assay

The MKN45 and MKN74 cells (4 × 10^5^/well) were cultured in 6-well 0.4 μm Transwell bottom chambers (2 mL) for the wound-healing assays to assess migration capabilities. CAFs (1 × 10^5^/well) and neutrophils treatment with or without NC/knockdown/overexpression IFIT1 (1 × 10^5^/well) seeded into the upper chamber with medium (2 mL) augmented with 10% FBS. Subsequently, the bottom chamber culture media was removed and wounds were induced in the cell monolayer using a 200 μL pipette tip. The rate of wound healing was evaluated at 0, 24, and 48 h using a phase-contrast microscope (Olympus CKX 41, Olympus, Hachioji, Japan).

### Cell counting kit 8 (CCK8) assay

Cell viability was determined with CCK8 assays. neutrophils were cultured in a 96-well plate (1 × 10^4^/well) and incubated in serum-free medium for 12–48 h. The CCK8 reagent was added according to the manufacturer’s protocol, and OD 490 was obtained from the plate reader. The sample size for CCK8 experiments is n = 3, and the individual experiment was replicated 3 times.

### Calcein-AM/PI living/dead cell double staining

To assess cell viability, live-dead staining was performed. neutrophils were labeled with calcein AM (live cells in green) and propidium iodide (PI, dead cells in red). Briefly, cell medium was removed and replaced by a mixture of calcein AM (CA, 4 µg/mL) and PI (1 µg/mL) diluted in a culture medium solution. After 30 min of incubation at 37 °C in a humidified atmosphere containing 5% CO2, the staining of living and dead cells was observed immediately under a fluorescence microscope (Nikon, DS-QilMC, Tokyo, Japan). The mean fluorescence intensity (MFI) of PI was used to estimate the statistical significance.

### Tubule formation assay

The tubule formation assay was performed as previously described, with slight modifications [23]. In brief, 50 μL of Matrigel was added to 24-well 0.4 μm Transwell bottom chambers for 30 min at 37 °C. HUVEC were seeded on Matrigel at 2 × 10^4^/well. Neutrophils (1 × 10^5^/well) seeded into the upper chamber with medium (500 μL) augmented with 10% FBS after treatment with or without NC/knockdown/overexpression IFIT1. Post 24 h of co-culturing Tubule formation was quantified using Angiogenesis Analyzer for ImageJ.

### Macrophages migration assay

Macrophage migration assays use Transwell chambers (8-μm pore size) in 24-well culture plates. 1 × 10^5^ macrophages in 100 μL RPMI-1640 medium per well in the upper chambers and 1 × 10^5^ neutrophils (Control NC sh-IFIT1 and oe-IFIT1) in 500 μL RPMI-1640 medium supplemented with 10% FBS were added into the lower chambers. The migration time of macrophages was stopped at 24 h, followed by fixation with 4% paraformaldehyde for 20 min. Cells from the upper surface of the wells were removed by cotton swabs, and the remaining cells in the wells were stained with crystal violet for 20 min. Cells were washed 3 times with PBS, and images were taken under an inverted microscope in five different areas per well and the cell number was counted using ImageJ software.

### WB

The WB protocol was executed according to the previously described [24]. Radioimmunoprecipitation (RIPA) buffer was utilized to extract proteins for cell lysis, and their quantification was accomplished using the Bradford assay. Each sample, comprising 20 µg, was isolated via Sodium dodecyl-sulfate polyacrylamide (SDS-PAGE) gel electrophoresis (10%) and subsequently transferred onto a Polyvinylidene fluoride (PVDF) membrane. Subsequently, the membrane was obstructed with BSA (5%), subjected to an overnight incubation with appropriate primary antibodies at 4 °C, rinsed thrice with Tris-buffered saline + Tween-20 (0.05%), and ultimately labeled with the corresponding secondary antibodies. The normalization of protein expressions was utilized β-actin as a reference.

### Enzyme-linked immunosorbent assay (ELISA)

The cytokine content was measured by ELISA (C-X-C Motif Chemokine Ligand 8, CXCL8; CXC chemokine receptor 2, CXCR2; Vascular endothelial growth factor A, VEGF-A; Nicotinamide phosphoribosyltransferase, NAMPT), CXCL2. The serum or culture medium supernatant was transferred into a fresh tube, and then centrifuged at 3500 rpm for 10 min at room temperature. ELISA kits were utilized as manufacturer’s instructions and previously published procedures [25]. The optical density (OD) value was measured at 450 nm using a microplate reader (Bio-Tek, ELX800, Winooski, VT).

### Mice xenograft tumor models

In the present study, we purchased a total of 70 C57BL/6 mice (male, 18 ~ 20 g, aged 8 weeks). We fed all the animals adaptively for 1 week under specific pathogen-free conditions, providing them with ad libitum food and water.

In order to study the effect of IFIT1 + neutrophils on the activation of T cells in mice, we established a xenograft tumor transplantation model in C57BL/6 mice. The Mouse Forestomach Carcinoma (MFC) cells (5 × 10^6^ cells/mouse) and Neutrophils (Control, NC, sh-IFIT1 oe-IFIT1, and 100ng/mL IFN-γ pretreated sh-IFIT1 group 5 × 10^5^ cells/mouse) were transplanted subcutaneously into the left axillary region of each mouse (n = 6) for 1 week to establish tumors (Day 7). When the tumors reached a size of 50 mm^3^, different groups of neutrophils (control, NC, sh-IFIT1, oe-IFIT1, and 100 ng/mL IFN-γ-pretreated sh-IFIT1 group 5 × 10^5^ cells/mouse) were injected into the tumor every other day, beginning on day 7 after inoculation. Day 28: We anesthetized tumor-bearing mice using CO_2_, following the American Veterinary Medical Association's (AVMA) Guidelines for Humane Animal Euthanasia. We collected and analyzed serum samples using flow cytometry.

In order to explore the underlying effect of IFIT1 + neutrophils on GC immunotherapy, The MFC cells (5 × 10^6^ cells/mouse) and neutrophils (NC, oe-IFIT1 5 × 10^5^ cells/mouse) were transplanted subcutaneously into the left axillary region of each mouse for 1 week to establish tumors (Day 7). The mice were intraperitoneally (i.p.) injected with 250 μg of anti-mouse PD-1 mAb. We administered the same volume of PBS to the mice in the comparison group. We injected different groups of neutrophils (oe-IFIT1 group and NC group) into the tumor every other day, starting from day 7 after inoculation. We injected anti-mouse PD-1 mAb or PBS 5 times at 3-day intervals into all mice. Tumor diameters were routinely measured using a caliper. Day 28: Tumor-bearing mice were sacrificed. Serum samples were collected and analyzed by flow cytometry, and tumor specimens were obtained for volume analysis using the formula V = 1/2ab^2^, as well as for generating growth curves.

### Statistical analysis

The correlation between variables was determined using Spearman correlation coefficients. For the analysis of variables that are not normally distributed, the Mann–Whitney U-test was used (also known as the Wilcoxon rank sum test). Student’s t test was used for two-group comparisons, and One-way analysis of variance (ANOVA) test was used for multiple-group comparisons. Survival analyses were performed using the log-rank (Mantel-Cox) test, and the corresponding Kaplan–Meier (KM) curves were plotted using “Survminer” package. Using univariate Cox regression, risk factors of independent prognostic value were identified, and hazard ratios (HRs) were calculated. R (version 4.1.2) and Excel (Microsoft) software were used to perform statistical analyses. A P value (two-tailed) of less than 0.05 was considered statistically significant.

## Results

### Cellular atlas of PCC and NPCC

**Fig. 1:**
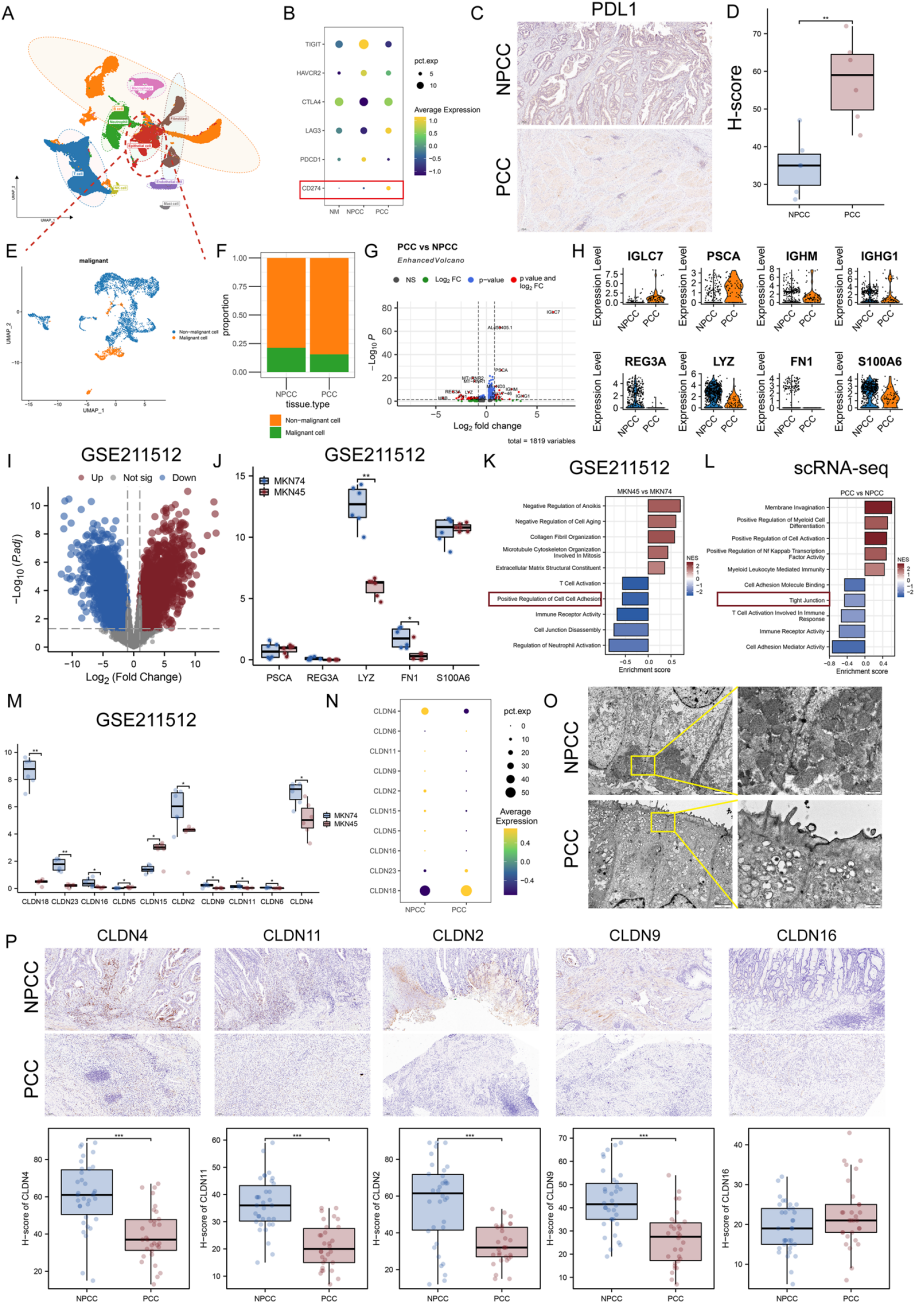
Representative single-cell transcriptome landscape of gastric cancer (GC). **A** Uniform Manifold Approximation and Projection (UMAP) plots showing cell types for the 64,454 cells. See also Fig. S3. **B** Bubble plots for the expression of immune checkpoints in all cells. CD274 is highlighted with a red box. **C** Immunohistochemistry (IHC) staining indicates the expression of PDL1 on PCC and NPCC tumor samples, Scale bars, 50 µm. **D** Box plots showing the expression (H-score) of PDL1 in NPP and NPCC patients (n = 12). **P < 0.01. **E** UMAP plot showing malignant and non-malignant cells in epithelial cells. See also Fig. S4. **F** The percentage of malignant and non-malignant cells in PCC and NPCC. **G** Volcano plot showing log2 fold change (FC) and the adjusted p value of differential genes between PCC and NPCC malignant cells (P < 0.01). **H** Violin plots showing the expressions of differential genes in malignant cells from PCC and NPCC samples. See also Fig. S5. **I** Volcano plot showing log2 FC and the adjusted p value of differential genes between PCC and NPCC cell lines in GSE211512 (P < 0.01). GSE211512 contains mRNA profiling data for the NPCC subtype cell line MKN74 and the PCC subtype cell line MKN45. **J** Box plots showing the expression of the top 8 differential genes (shown in Fig. 1H) in tumor samples for various xenograft tumors treated with MKN45 (SRC cells) vehicle and MKN74 (moderately differentiated adenocarcinoma cells) vehicle (n = 14) *P < 0.05, **P < 0.01. **K**, **L** The most enriched pathways for up/downregulated genes for malignant cells from samples in our own single-cell RNA data (**K**), and GSE211512 (**L**), respectively. Considering the well-known properties of PCC cells, cell junction-associated terms are highlighted with red boxes. **M**, **N** Box and bubble plots showing the expression of tight junction proteins in single cell data (**M**) and GSE211512 (**N**), respectively. *P < 0.05, **P < 0.01. **O** Ultrastructure of tight junctions (TJs) was observed by transmission electron microscope (EM) in PCC and NPCC. **P** Immunohistochemistry (IHC) staining indicates the expression of TJ proteins on PCC and NPCC tumor samples, Scale bars, 100 µm. ***P < 0.001

To characterize the cell populations and associated molecular features of PCC and NPCC, pathology sampling strategies and dropwise scRNA-seq (10X genomic) were used to generate single-cell data on surgically resected GC specimens consisting of six NPCC samples, three PCC samples, and three paired control samples (normal). After strict quality control (see “Materials and methods” section), 64,454 cells were finally retained for further biological analysis. After normalization by gene expression and dimension reduction, we classified cells into 31 clusters using graph-based clustering (see “Materials and methods” section). These clusters can be annotated by recognized marker genes (Figs. 1A and S3A-B) into nine known cell lineages: epithelial cells (marked by Keratin 18, *KRT18*), T cells (marked by CD3 Delta Subunit of T-Cell Receptor Complex, *CD3D*), B cells (marked by CD79a Molecule, *CD79A*), Neutrophils (marked by S100 Calcium Binding Protein A9, *S100A9*), endothelial cells (marked by Endoglin, *ENG*), fibroblasts (marked by Collagen Type I Alpha 2 Chain, *COL1A2*), macrophages (marked by CD14 Molecule, *CD14*), mast cells (marked by Carboxypeptidase A3, *CPA3*), and NK cells (marked by Fc Gamma Receptor IIIa, *FCGR3A*). Unlike NPCC, PCC has unique histologic and molecular features. Here we observed that the proportions of neutrophils and macrophages, were higher in PCC samples, where the proportion of epithelial cells decreased significantly (Fig. S3C-D).

The overall extremely poor prognosis of PCC may be due to the highly immunosuppressive TME features, which are often as a result of upregulation of immune checkpoints. In general, the expression levels of multiple immune checkpoints (Programmed Cell Death 1, *PDCD1*; T Cell Immunoreceptor with Ig and ITIM Domains, *TIGIT*; Cytotoxic T-Lymphocyte Associated Protein 4, *CTLA4*; Hepatitis A Virus Cellular Receptor 2, *HAVCR2*; Lymphocyte Activating 3, *LAG3*; CD274 Molecule, *CD274*) are indicators of the degree of immunosuppression. Therefore, we compared the differences in immune checkpoint gene expression among different tissue types. Interestingly, *CD274* (PDL1) was found to have the highest level in PCC samples (Fig. 1B), and IHC staining further confirmed this result (Fig. 1C, D, P < 0.01). These data suggested that the PDL1 pathway may be a crucial mediator of local immunosuppression in the PCC tumor microenvironment.

We then estimated the Copy number variation (CNV) levels of all epithelial cells using epithelial cells from normal samples as a reference (Figures S4A-B). To distinguish malignant cells from non-malignant cells in our data, clustering, tissue origin, and CNV information were combined, resulting in 2 clusters of malignant cells and 7 clusters of non-malignant cells (Figs. 1E and S4C-D). The proportion of malignant cells was observed to be slightly lower in PCC samples than in NPCC samples (Fig. 1F). We further compared the transcriptional profiles of PCC and NPCC cells to clarify their molecular differences (Fig. 1G). As for DEGs of malignant cells from PCC and NPCC, the expressions of Regenerating Family Member 3 Alpha (*REG3A*), Lysozyme (*LYZ)*, Fibronectin 1 (*FN1*) and S100 Calcium Binding Protein A6 (*S100A6*) were significantly higher in NPCC, while Immunoglobulin-related genes such as Immunoglobulin Lambda Constant 7 (*IGLC7)*, Prostate Stem Cell Antigen (*PSCA*), Immunoglobulin Heavy Constant Mu (*IGHM*) and Immunoglobulin Heavy Constant Gamma 1 (*IGHG1*) were specifically expressed in PCC (Fig. 1H). It has been suggested that IGLC7 may work synergistically with immune checkpoints to regulate the immune microenvironment [26]. Immunoglobulin G (IgG) is widely expressed in many cancers and it promotes cancer progression [27]. IGHG1 was shown to induce EMT in SGC7901 cells by regulating the TGF-β/SMAD3 signaling pathway [28]. Cox regression analysis showed that *FN1* (Overall survival, OS and Disease-specific survival, DSS) and Prostate Stem Cell Antigen (PSCA) (Progression-free interval, PFI) were significant prognostic factors for GC (Fig. S5A-C, P < 0.05). Importantly, the expression of PSCA in cancerous tissues correlates with the degree of tumor differentiation. FN1 was identified to be specifically overexpressed in the tumor stroma and involved in the formation of a fibrous network suitable for tumor growth [29, 30]. It’s noteworthy that, as a cell adhesion molecule, alterations in FN1 expression were frequently manifested in tumor cells, and thus we hypothesized that the lack of FN1 expression might be related with the poor cohesion of tumor cells [31]. We further analyzed the correlation between these differential molecular and clinical features in the TCGA-STAD cohort, including tissue type, T/N/M staging, pathological stage, tumor grade, and histological type. Comparing the mRNA expression levels of top 8 DEGs in tissue samples, *IGHG1* and *S100A6* were found at higher expression levels in cancer tissues (Fig. S5D, P < 0.001). We noted that the genes upregulated in PCC cells almost always showed positive correlations with clinical parameters, especially *IGHG1* (Fig. S5E-I, P < 0.05, with T stage, pathological stage, and tumor grade). Furthermore, we observed that the expression level of the *IGHG1* gene in 13 SRC cases was higher than that in 72 diffuse gastric adenocarcinoma cases, indicating the distinctive significance of *IGHG1* (Fig. S5J, P < 0.05). Previously we established the USP51-ZEB1-ACTA2 mesenchymal signaling axis in PCC, and interestingly, here we found that IGHG1 was positively correlated with this signaling intensity (Fig. S5K; *IGHG1*-*USP51*, R = 0.214, P < 0.001; *IGHG1*-*ZEB1*, R = 0.153, P < 0.001; *IGHG1*-*ACTA2*, R = 0.139, P = 0.007). To validate the differential expression results obtained in scRNA-seq, we introduced an additional dataset, GSE211512, containing sequencing data of MKN45 (SRC cells) vehicle and MKN74 (moderately differentiated adenocarcinoma cells) vehicle-treated tumor samples for various xenograft tumors. As shown in Fig. 1I, J, further results indicated that *FN1* and *LYZ* were significantly low-expressed in PCC tumors (P < 0.05).

Considering the finding that PCC cells and other types of GC cells differ in cell type of origin, the features of PCC cells should be investigated. Thus, we performed GSEA. In our own data, compared with NPCC, several cell junction and immune-related terms such as Cell Adhesion Molecule Binding, Tight Junction, Cell Adhesion Mediator Activity, T Cell Activation Involved in Immune Response, and Immune Receptor Activity were down-regulated in PCC (Fig. 1K). In GSE211512, we also observed the down-regulation of cell junctions and immune-related signaling (Fig. 1L). We suggested that immunosuppression and poor-cohesiveness were two of the most essential characteristics of PCC tumors. Considering that malignant transformation leads to the disorganization of Tight junctions (TJs) on the surface of tumor cells, we further compared the differences in TJ molecules (claudins) between the two tissue types of tumors. As shown in Fig. 1M (GSE211512) and Fig. 1N (our own data), most of the TJ molecules were determined to be significantly down-regulated in PCC tumors, suggesting that less TJs may be formed in PCC tumors, leading to greater aggressiveness. Since the expression of TJ protein is mainly regulated by post-transcriptional regulatory processes, further experimental validation was carried out. Imaging of TJ structures by transmission electron microscopy (TEM) showed that stable TJs were still observed in NPCC biopsy specimens, whereas in PCC biopsy specimens the TJs disappeared completely (Fig. 1O). In addition, TJ protein expression in PCC and NPCC tissues was analyzed using IHC staining. The information of GC patients suitable for study was demonstrated in Table S10. We observed similar results that TJ proteins were lowly expressed in PCC tissues, especially CLDN4, CLDN11, CLDN2, and CLDN9 (Fig. 1P, P < 0.001). These findings suggest the disruption of the structure of TJs may be an important feature of PCC.

### hdWGCNA defines a *CD274*-related neutrophil gene module

**Fig. 2:**
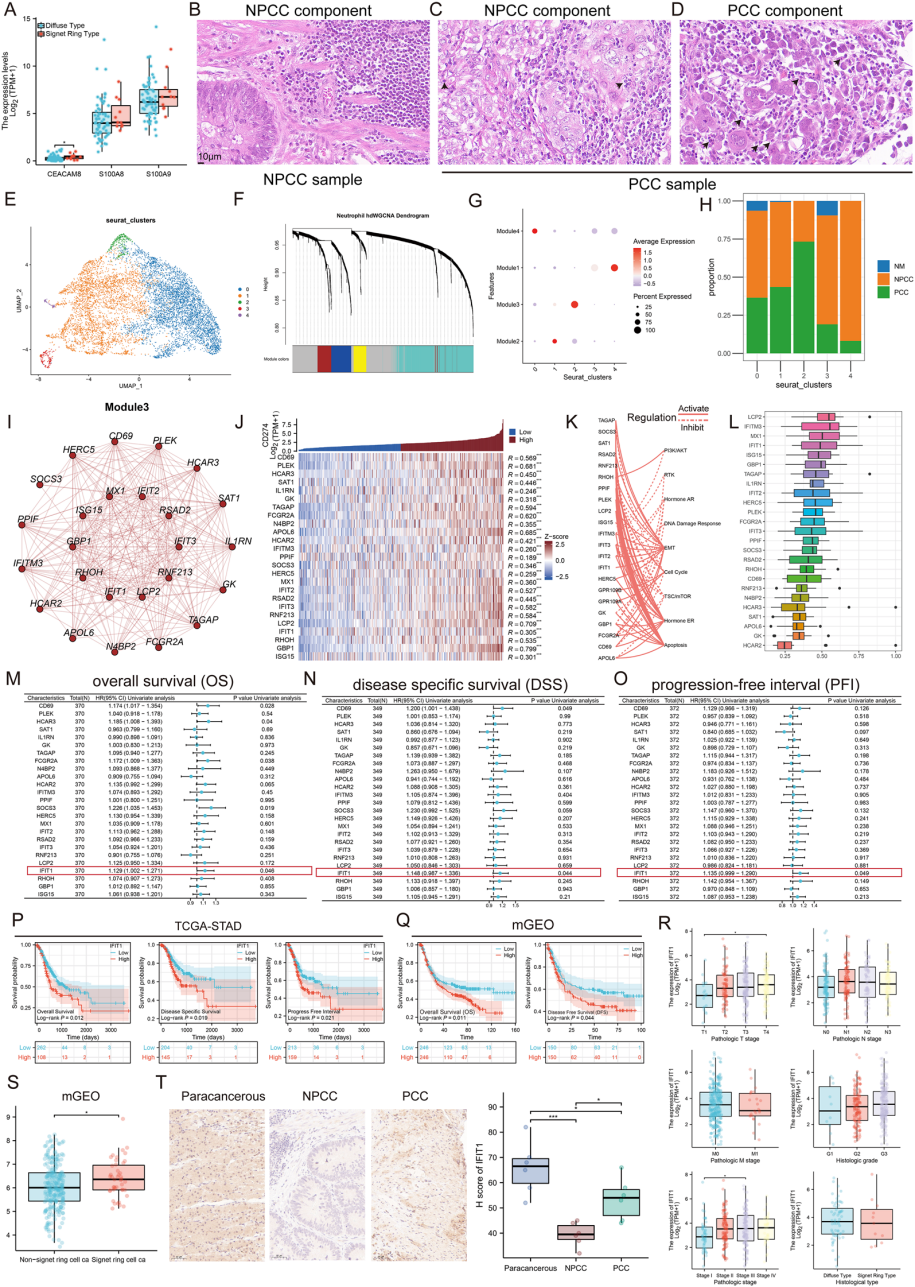
high-dimensional Weighted Gene Co-expression Network Analysis (hdWGCNA) defines a CD274-related neutrophil gene module, which represents PCC-specific neutrophil infiltration. **A** Box plots showing the expression of neutrophil markers in generally diffuse type and signet ring GC patients (TCGA-STAD, n = 74). *P < 0.05. **B**–**D** Image representing the pathological Hematoxylin/eosin (HE) staining variation among the NPCC component in NPCC sample (**B**), NPCC component in PCC sample (**C**), and PCC component in PCC sample (**D**) (n = 12) (Scale bars, 10 μm). **E** UMAP plot of 8638 neutrophils, colourcoded for five molecular clusters. See also Fig. S6. **F** Highly variable genes were clustered into 4 modules through hdWGCNA. See also Fig. S7. **G** Dot plot showing the different module scores in neutrophils. **H** Fraction of three tissue types in five cell subgroups. **I** Module 3 is valued for its PCC tissue specificity, demonstrating a specific network structure. **J** Heatmap showing the correlations between CD274 and Module 3 members. Red represents positive values and blue represents negative, normalizing gene expression to a Z-score. See also Fig. S8. **K** Correlation of 25 module-3 molecules in GC with important cancer signaling pathways. Solid lines indicate activation and dashed lines indicate inhibition. **L** Friends analysis of module-3 genes. **M**–**O** Forest maps showing the results of Cox regression analysis on the average overall survival (OS) (**M**), disease specific survival (DSS) (**N**), and progression free interval (PFI) (**O**) rate of 25 module 3-related molecules in the TCGA-STAD cohort. **P**, **Q** Survival analysis revealed that patients with high IFIT1 expression had shorter OS, DSS, and Progression-free interval (PFI) in TCGA-STAD (**P**) and OS, and disease-free survival (DFS) in mGEO (**Q**). **R** The expression level of *IFIT1* among different clinical features was analyzed in TCGA-STAD (Kruskal-Walli’s test). *P < 0.05. **S** The differences in *IFIT1* gene expression between non-signet ring cell carcinoma (ca) cases and signet ring cell ca cases in mGEO (Wilcoxon rank sum test). *P < 0.05. **T** IHC staining (left) and box plots (right) indicate the expression of IFIT1 on Paracancerous samples, PCC, and NPCC tumor samples, Scale bars, 50 µm. *P < 0.05, ***P < 0.001

Previous results showed PCC samples contained high levels of infiltrating neutrophils (Figure S3D). We compared neutrophil marker levels in TCGA samples and found that CEA Cell Adhesion Molecule 8 (*CEACAM8*, CD66b), S100 Calcium Binding Protein A8 (*S100A8*), and *S100A9* were all upregulated in SRC samples, especially *CEACAM8* (Fig. 2A, P < 0.05). As the most abundant leukocytes in the blood, neutrophils not only perform anti-microbial functions in injury or infection, but are also highly infiltrated in many types of tumors [32–34]. Due to the lengthy tumor growth and the short lifespan of neutrophils, early investigators considered neutrophils to be mere bystanders in tumors [35]. However, recent evidence suggests that neutrophils have a biphasic function in the antitumor immune response and play a role in the fate of tumors [36, 37]. Pathological sections of GC seemed to exhibit elevated levels of neutrophil infiltration in the PCC component (Figs. 2B–D, black arrows). Based on this phenomenon, we performed a cluster analysis of 8638 neutrophils and identified five prominent cell subpopulations (Fig. 2E, Cluster 0-Cluster 4). Neutrophils infiltrating solid tumors [tumor-associated neutrophils (TANs)] can be broadly defined as phenotypes displaying inflammatory and antitumor properties (TAN1) or phenotypes associated with tumor progression (TAN2) [38]. Interestingly, we observed that cluster 2 exhibited enriched expression of Intercellular Adhesion Molecule 1 (*ICAM1*, N1 marker) and Vascular Endothelial Growth Factor A (*VEGFA*, N2 maker) (Figure S6A, B). Upregulation of ICAM1, an immunoglobulin (Ig)-like cell adhesion molecule, enhances neutrophil antitumor capacity, while neutrophil-induced VEGFA promotes proliferation, invasion, and angiogenesis. To further explore the potential functions of neutrophils, hdWGCNA was applied and detailed information of gene modules was shown in Figure S7A–C. As shown in Fig. 2F, four gene modules were obtained, with the top hub gene presented along the hdWGCNA pipeline. We then assessed the module scores of neutrophil clusters (Figs. 2G and S7D-E). Enrichment analysis showed that the four gene modules had different functions (Figure S7F). Interestingly, module 3 was highly activated mainly in cluster 2. Considering the specificity of cluster 2, we quantified the proportion of cells from different tissues in each cluster. Notably, the majority of cells in cluster 2 were sourced from PCC samples (Fig. 2H). We generated specific module networks showing the gene members in each module (Figs. 2I and S7G-I). Besides, Fig. S7J demonstrates the interactions between modules, with Radical S-Adenosyl Methionine Domain Containing 2 (*RSAD2*) and *IFIT1* highlighted because of their importance in the network. It has been shown that there is a clear correlation between PDL1 expression and neutrophils that exert immunosuppressive functions [39]. Considering that cluster 2 may be associated with the immunosuppressive state of PCC, we sought to investigate the bulk correlation of *CD274* (PDL1) and module-3 members. In order to verify the stability of all calculations based on publicly available data, we additionally included a cohort (mGEO). The gene expression baseline of each GEO cohort is shown before (Figure S8A) and after (Figure S8B) the batch effect correlation. The results indicated that the batch effect had been effectively corrected. In TCGA-STAD, the module-3 gene was consistently positively correlated with *CD274*, and a similar phenomenon was observed in the independent mGEO data (Figs. 2J and S8C). These results implied that module 3 may function in regulating immunity through PDL1-related pathways. We further explored the characterization of module-3 related signaling pathways. In GC tissues, most of the molecules activated apoptosis, EMT and hormone estrogen receptor (ER) signaling pathways, but inhibited TSC/mTOR signaling pathway (Fig. 2K). To identify key regulators in Module 3, Friends analysis and COX regression were performed separately. The results showed that *IFIT1* was not only strongly correlated with other members (Fig. 2L), but also an independent prognostic factor for GC (Figs. 2M–O; OS, P = 0.046; DSS, P = 0.044; PFI, P = 0.049). We further categorized all GC samples into *IFIT1* high-expression and low-expression groups based on the optimal cutoff value. The phenomenon that GC patients in the *IFIT1* high-expression group had a relatively shorter OS than the low-expression group was observed using survival analysis (Fig. 2P, TCGA-STAD, P < 0.05). Similar results were also observed in mGEO (Fig. 2Q, OS and DFS, P < 0.05). Figure 2R shows further correlation between *IFIT1* expression and clinicopathologic features of GC patients, indicating that *IFIT1* was positively correlated with several gastric cancer classifications, i.e., T classification for TNM staging and clinical stage (TCGA-STAD, P < 0.05). Notably, due to the small sample size (only 13 SRC samples in TCGA-STAD), we were unable to determine the statistical significance of the histological type differences in IFIT1. Therefore, samples of mGEO were included to further compare differences. As expected, *IFIT1* was significantly upregulated in the SRC samples (Fig. 2S, P < 0.05). In addition, immunohistochemical techniques were applied to detect IFIT1 expression in 6 NPCC and 6 PCC tissues, which further confirmed the high expression of IFIT1 in PCC samples (Fig. 2T, P < 0.05). Of note, there is previous evidence that a subpopulation of IFIT1 + neutrophils showed high PDL1 expression, which may be due to the interaction of IFIT1 + cells with Interferon Gamma (IFNG) + lymphocytes [40]. In addition, m6A-modified IFIT1 was found to induce PDL1 upregulation in an independent colorectal cancer model. Thus, these evidences pointed to a regulatory mechanism between IFIT1 and PDL1 [41].

### Module-3 activation is associated with high stromal component of PCC tumors and is a detrimental factor in the prognosis of GC patients

**Fig. 3:**
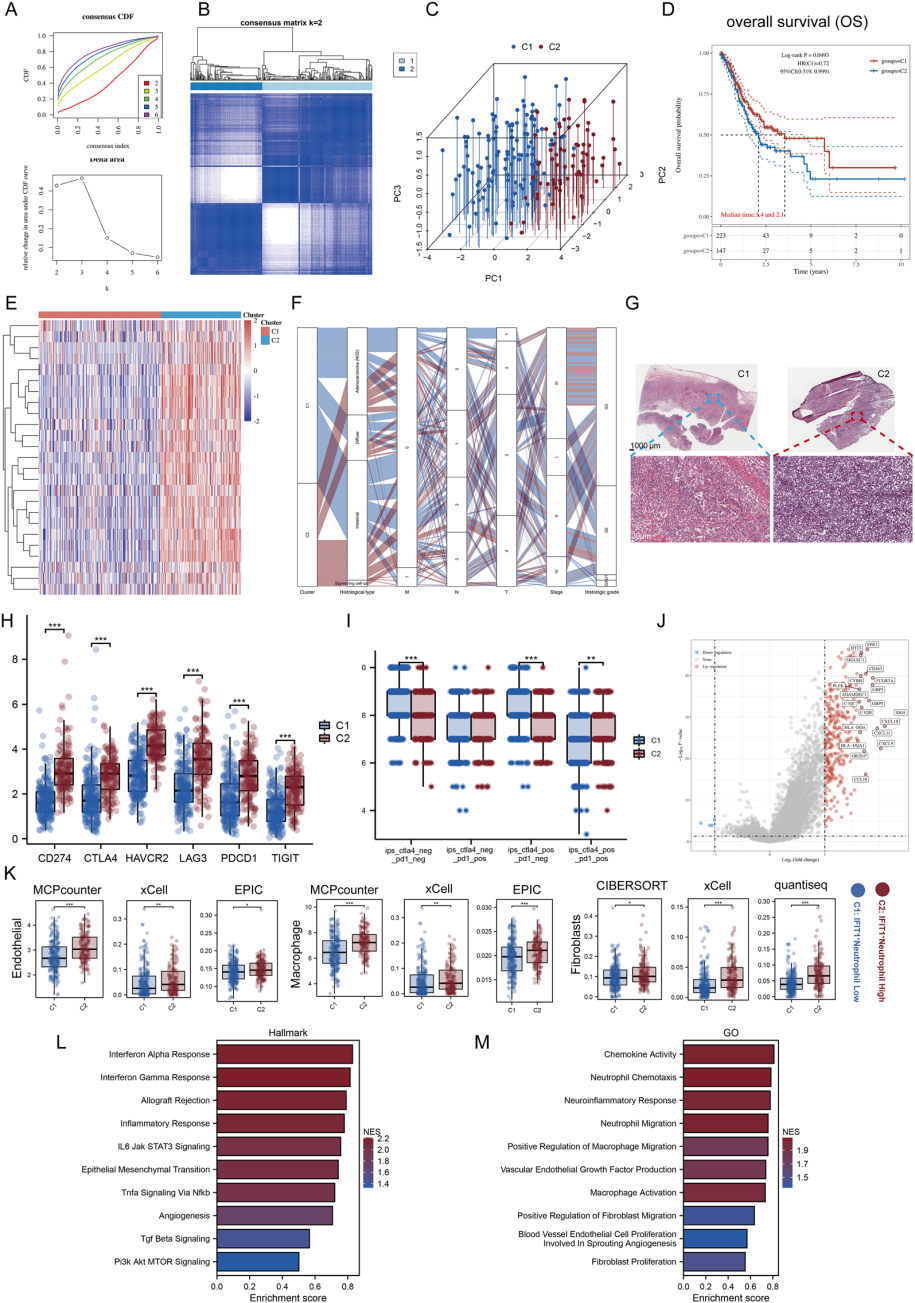
Unsupervised learning to identify two classification keywords by module 3. See also Figs. S9-S11. **A**, **B** The consensus clustering matrix for k = 2 was determined by Cumulative distribution function (CDF) for k = 2–6. **C** Principal component analysis (PCA) showed that the expression of the 25 module-3 molecules defines distinct phenotypes. **D** The Kaplan–Meier curve shows significant OS rate differences between the two kinds of phenotypes in TCGA-STAD. **E** Heatmap showing expression differences of Module-3 molecules between the two clusters. **F** Sankey diagram showing the correlation between GC classifications and clinical parameters. **G** Image representing the pathological HE staining variation between different phenotypes (TCGA database) Scale bars, 1000 μm (panoramic view) and 20 μm (partial view). **H**, **I** Box plots showing the expression of the immune checkpoint genes and Immune cell Proportion (IPS) Score in different phenotypes (TCGA-STAD, n = 375). **P < 0.01, ***P < 0.001. **J** Volcano plot showing log2 FC and the adjusted p value of differential genes between different phenotypes in TCGA-STAD. Selected highly significant genes are labeled. **K** Box plots showing the variations in levels of pro-tumor cell infiltration we focus on. *P < 0.05, **P < 0.01, ***P < 0.001. **L**, **M** Gene set enrichment analysis (GSEA) of the status of special biological pathways based on HALLMARK (**L**) and GO (**M**) in two module-3 phenotypes

Here, we sought to link IFIT1 + TANs to the clinicopathologic and TME features of GC. In order to understand the integrated molecular mechanism of Module 3 in GC, we defined two unique modification patterns, named Cluster 1 (C1, 227 cases) and Cluster 2 (C2, 148 cases), in the TCGA-STAD cohort using the “ConsensusClusterPlus” package (Figs. 3A, B and S9A-D). The principal component analysis showed Bhat the two clusters could be effectively distinguished by the expression profiles of the 25 Module-3 molecules (Fig. 3C). Survival analysis showed that C2 had a worse prognosis than C1 (OS, P = 0.0493, Fig. 3D). The heatmap showed that C2 was characterized by high activation of module 3 (Fig. 3E). We compared our molecular phenotypes with several commonly used clinical parameters. C2 contained more advanced patients (T4 and stage IV) than C1. Importantly, all of the SRC carcinoma samples (n = 13) were included in C2 (Fig. 3F). Pathological sections confirmed the presence of a significant PCC component in the tumor tissue of the C2 patient, which was absent in the C1 sample (Fig. 3G). Since previous results showed higher overall PDL1 levels in PCC, we went on to compare the PDL1 differences between the two clusters. As expected, the PDL1 levels were higher in C2 (Fig. 3H). More importantly, the IPS scoring system showed that ctla4_pos_pd1_pos immune cells level was significantly higher in C2 than in C1 (P < 0.01). However, the scores of ips_ctla4_neg_pd1_neg and ips_ctla4_pos_pd1_neg were significantly higher in the C1 (P < 0.001, Fig. 3I). These data suggested that C2 may have a highly activated PD1-PDL1 ligand-receptor pair (PD1 and PDL1 may bind more frequently), as does PCC.

Other studies indicated that PCC patients have a lower Tumor mutational burden (TMB) than NPCC patients [14]. Two subgroups of the mutational landscape were analyzed using the TCGA-STAD cohort. We found seven genes (Tumor Protein P53, *TP53*; Titin, *TTN*; Mucin 16, *MUC16*; LDL Receptor Related Protein 1B, *LRP1B*; Spectrin Repeat Containing Nuclear Envelope Protein 1, *SYNE1*; CUB And Sushi Multiple Domains 3, *CSMD3*; Filaggrin, *FLG*) with high mutation frequencies in both subtypes. Oncoplot shows the mutation spectra of the genes with the highest mutation frequencies in each cluster (Fig. S9E, F). In both subgroups, *TTN* and *TP53* were usually observed to have frequent missense mutations. Specifically, C2 had more AT-Rich Interaction Domain 1A (*ARID1A*), AHNAK Nucleoprotein 2 (*AHNAK2*), and FAT Atypical Cadherin 4 (*FAT4*) missense mutations than C1. Interestingly, we found more mutations co-occurring in C1, such as *TTN* and Spectrin Alpha Erythrocytic 1 (*SPTA1*) mutations (Fig. S9G), while the only mutually exclusive mutational events (*ARID1A* and *TP53*) were observed in C2 (Fig. S9H). We focused on the mutation site differences of TP53 in the two subgroups, and noticed that C1 has more transactivation domain (TAD) and tetramer mutations (Fig. S9I).

In addition to the immune checkpoint-related mechanisms discussed above, there are other mechanisms of immune evasion mediated by immune/stroma-associated cells. We used the “Limma” R package to obtain DEGs between C1 and C2 and found that a large number of chemokines such as C-X-C Motif Chemokine Ligand 10 (*CXCL10*), *CXCL11*, *CXCL9*, and *CCL18* were upregulated in C2. In addition, high expression of *CD163*, which is generally recognized as a marker of macrophage anti-inflammatory phenotype, was found in C2 (Fig. 3J). These results reflected the impact of module 3 on the tumor immune microenvironment. Therefore, we further estimated the abundance of immune cell infiltration in GC samples using bulk data by Cibersort, Quantiseq, Timer, Mcp_counter, Epic, and Xcell algorithm. Specifically, we used the “BisqueRNA” package to deconvolve the bulk data to quantify the level of IFIT1 + (cluster 2) neutrophils in GC samples (Fig. S9J–M). The results showed an overall higher level of immune infiltration in C2 (Fig. S10A, B). Our group previously reported that PCC cells have a strong ability to recruit tumorigenic mesenchymal cells, including fibroblasts, M2 macrophages, and vascular endothelial cells. Therefore, we focused on examining the level of infiltration of these three cell types. As expected, higher levels of fibroblasts, M2 macrophages, and endothelial cells were found in C2 (Fig. 3K). As a portion of our study, we further confirmed that these cells were predictive of poor prognosis and linked them to IFIT1 + neutrophils (which is what this analysis concerns. We also used the “Estimation” algorithm for validation, as the immune score and stromal score obtained by the estimation algorithm compared the differences in the immune and stromal components in the TME subtypes. It can be seen that C2 has a higher immune score and stromal score. Importantly, the higher the percentage of non-tumor components, the worse the prognosis of tumor patients tends to be. In addition, to verify the reproducibility of the clustering, we repeated the above process in the mGEO dataset (Fig. S11). Consistent with the results in TCGA, hyperactivation of module 3 was highly associated with SRC. It is also noteworthy that, compared with the ACRG subtype, C2 is mostly MP and EMT subtypes. Invasive subtypes were also predominant in C2 compared with Singapore subtype (GSE15459), with mostly Proliferative subtypes in C1. Previous studies have shown that EMT and Invasive subtypes are prevalent in young adults and contribute to drug resistance and metastasis after cancer chemotherapy [42]. Another work from our team also confirmed that PCC has an aberrantly activated EMT program (highly expressed EMT transcription factor ZEB1). This may somehow suggest a potential correlation between the module-3 activation subgroup (C2) and PCC subtypes. As our molecular typing exhibited a high degree of correlation with clinicopathological features, additional GSEA was performed to explore its relationship with biological pathways, thus revealing functional differences between the two clusters. Ten up-regulated pathways in C2 were obtained. Among them, for HALLMARK, macrophage-, fibroblast-, vascular endothelial cell-, and neutrophil-associated signals were significantly enriched in C2; and for GO, C2 had principal enrichment in EMT-, interferon-, TNFα-, and angiogenesis-associated signals (Fig. 3L, M).

### In PCC, neutrophils may interact more frequently with stromal and immune cells through multiple signaling mechanisms

**Fig. 4:**
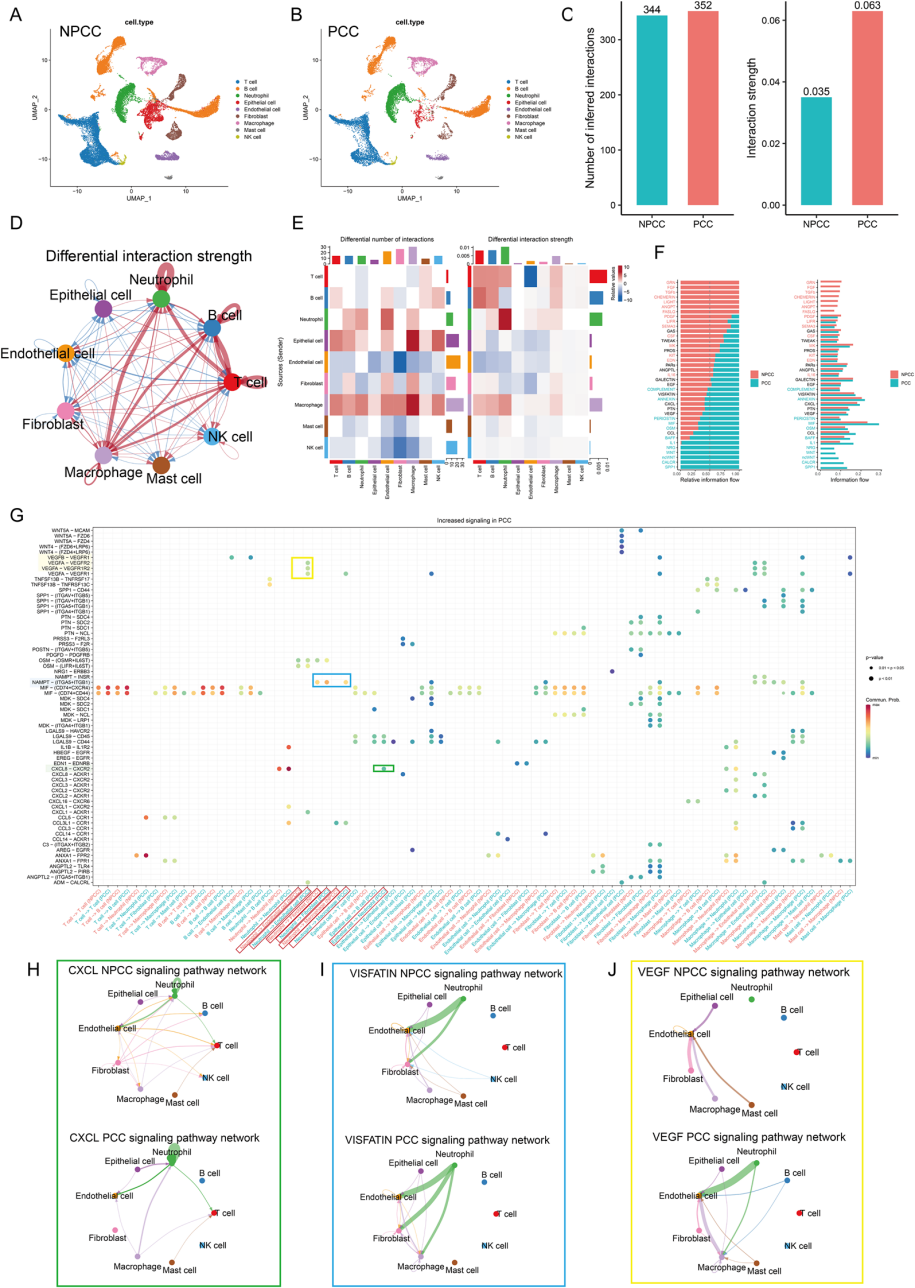
CellChat identifies the differences in cell–cell communications between PCC and NPCC samples. See also Fig. S12. **A**, **B** UMAP plot showing the distribution of cells from NPCC (**A**) and PCC (**B**) samples. **C** The difference in cell–cell interaction number and strength between NPCC and PCC samples was analyzed based on CellChat. **D**, **E** Circle plot (**D**) and heatmap (**E**) showing the difference in the number and intensity of interactions per cell type at NPCC and PCC samples. **F** Differences in the cell–cell interaction information flow in different groups (NPCC and PCC). **G**–**J** Bubble diagram and circle diagram showing that fibroblasts, macrophages, endothelial cells and epithelial cells may communicate with neutrophils frequently through multiple signals in PCC samples. Important signal pairs and cell pairs were highlighted with boxes

Next, we investigated the factors intrinsic to the differences in TME between PCC and NPCC samples (Fig. 4A, B). The “CellChat” package was used to compare cellular interactions between the PCC and NPCC groups. The results showed that there was no difference in the number of receptors or ligands between the two groups, and the interaction was significantly stronger in the PCC group than in the NPCC group (Fig. 4C). The number and strength of signaling pathways between neutrophils/macrophages and other cells were significantly higher in the PCC group than in the NPCC group (Fig. 4D, E). We compared the information flow of each signaling pathway between the two groups and found that Secreted Phosphoprotein 1 (*SPP1*), Calcitonin Receptor (*CALCR*), and WNT-related signals were enriched in PCC, whereas Granulin Precursor (GRN), Fibroblast Growth Factor (FGF), and Transforming Growth Factor Beta (TGFb)-related signals were enriched in NPCC (Fig. 4F). Notably, SPP1 is associated with a non-inflammatory, immunosuppressive phenotype of macrophage activation and WNT is a well-known inducer of EMT [43, 44]. Considering the significant neutrophil infiltration features of PCC samples, we focused here on the potential impact of neutrophils on TME. Further analysis showed that the strength of signaling pathways from neutrophils to fibroblasts, macrophages, and endothelial cells was significantly higher in the PCC group and that the strength of signaling pathways from epithelial cells to neutrophils was also higher than in the NPCC group (Figs. 4G and S12). Figure 4H–J illustrates the specific communication networks we focused on (highlighted with boxes in Fig. 4G) for CXCL, (Nicotinamide Phosphoribosyltransferase, NAMPT also called VISFATIN), and Vascular Endothelial Growth Factor (VEGF) signaling. Based on these data, we hypothesized that activation of Module 3 may maintain a high infiltration of stromal cells in TME through a communication network centered by neutrophils, just as shown in Fig. 3K.

### IFIT1 + TAN is highly infiltrated in PCC and upregulates PDL1 expression

**Fig. 5:**
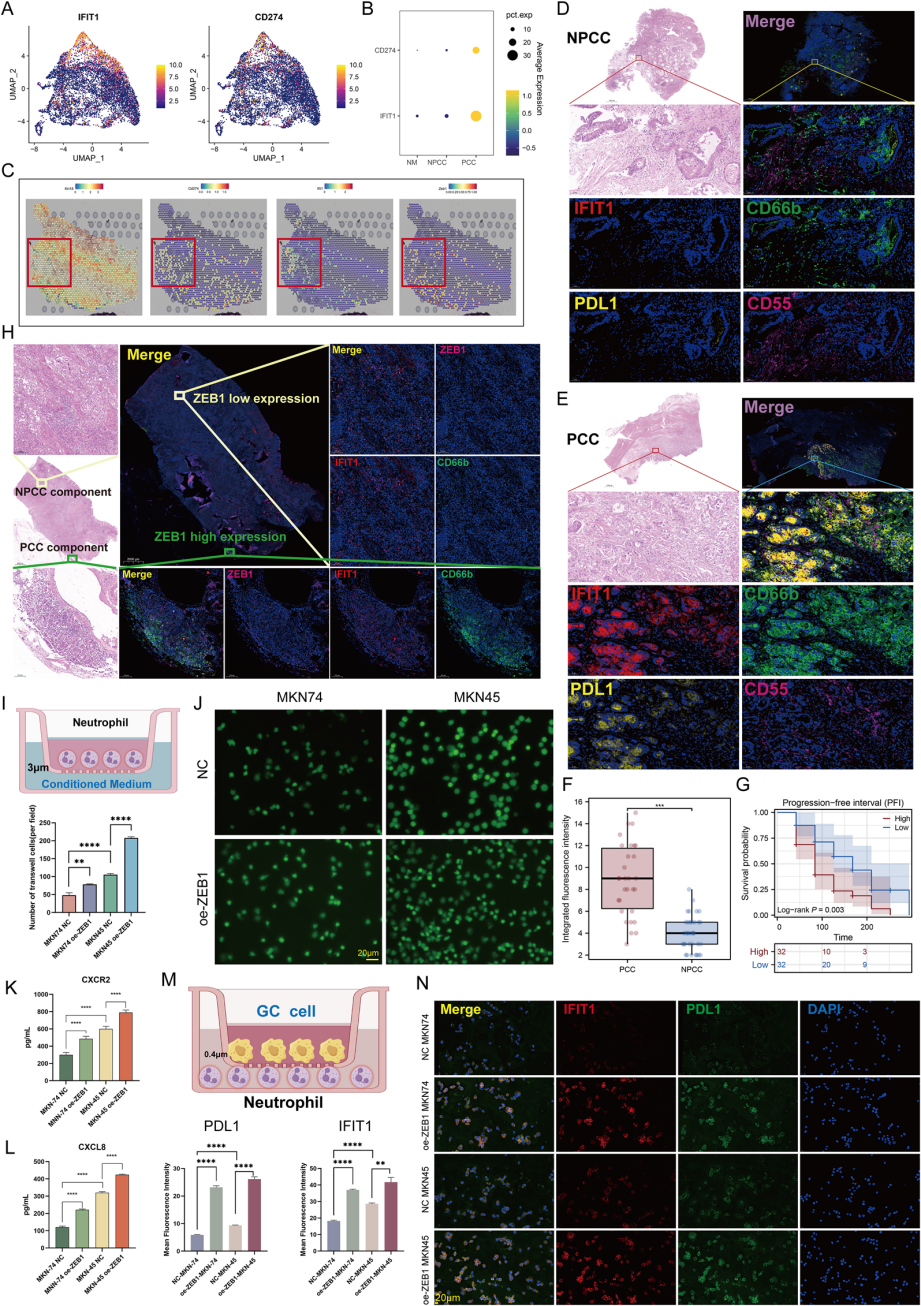
Interferon Induced Protein with Tetratricopeptide Repeats 1 (IFIT1) + TANs are highly infiltrated in PCC tissues to exert potential immunosuppressive functions and are educated by PCC cells. **A** UMAP plots of *IFIT1* (left) and *CD274* (right) in neutrophils. **B** Bubble plots for the expression of IFIT1 and CD274 in neutrophils among different tissue types. **C** Spatial transcription sections show the spatial expression of Keratin 18 (*KRT18*) (epithelial marker), *CD274*, *IFIT1*, and PCC marker Zinc Finger E-Box Binding Homeobox 1 (*ZEB1*). The dot color represents the expression level of the genes. **D**, **E** Multiple immunofluorescence (mIF) staining of PDL1, IFIT1, and neutrophil marker CD66b and CD55 in NPCC (**K**) and PCC (**L**) samples. Representative regions are indicated by in the enlarged images at right. Scale bars, 1000 μm (left) and 100 μm (right). **F** Differences in IFIT1 + neutrophils infiltration levels (IFIT1 + CD66B + CD55 integrated fluorescence intensity) between PCC and NPCC (n = 64, 30 PCC cases and 34 NPCC cases). ***P < 0.001. **G** Survival analysis revealed that patients with high IFIT1 + neutrophils levels had a shorter PFI (n = 64, 30 PCC cases and 34 NPCC cases). **H** mIF staining of ZEB1, IFIT1, and CD66b in PCC samples. Representative regions are indicated by in the enlarged images at right, and the PCC components (ZEB1 +) and NPCC (ZEB1-) components are distinguished by HE staining. Scale bars, 1000 μm (panoramic view) and 100 μm (partial view). See also Fig. S13. **I** Schematic drawing of the co-culture model [neutrophils (above) and different GC cell supernatants as conditioned medium (below)]. **J** Neutrophil chemotaxis assays were performed using Transwell chambers (3-μm pore size), in which the conditioned medium was derived from MKN45 and MKN74 cells overexpressing ZEB1. Scale bars, 20 μm. **P < 0.01, ****P < 0.0001 vs. respective control by t-test. **K**, **L** Enzyme-linked adsorbent assay (ELISA) assay in co-culture system with the presence or absence of oe-ZEB1 in MKN45 and MKN74. CXC chemokine receptor 2 (CXCR2) (Q) C-X-C Motif Chemokine Ligand 8 (CXCL8) (R). ****P < 0.0001 vs. respective control by t-test. **M** Schematic drawing of the co-culture model [tumor cells (above) and neutrophils (below)]. **N** mIF of IFIT1 and PDL1 expression in neutrophils with the intervention of MAKN45 and MKN74 cell lines overexpressing ZEB1. Scale bars, 20 μm. **P < 0.01, ****P < 0.0001 vs. respective control by t-test. The data represent three independent experiments

Since the above results suggested that IFIT1 + TANs may be a unique effector mechanism of PCC, we further investigated the potential function of IFIT1 + TANs here. Previously the transcriptional correlation between module 3 and *CD274* was confirmed and we further highlighted the co-expression of *IFIT1* and *CD274* at the single cell level. Uniform Manifold Approximation and Projection (UMAP) plots showed that *IFIT1* and *CD274* were upregulated in cluster-2 neutrophils (Fig. 5A), and bubble plots showed that both were at significantly higher levels in neutrophils derived from PCC than in those derived from other tissue samples (Fig. 5B). These results further implied that IFIT1 + TANs may play an immunosuppressive role in PCC.

To verify the specific infiltration of IFIT1 + PDL1 + TANs in PCC samples, we analyzed the potential physical interactions between IFIT1 + TANs and PCC cells. As a well-known EMT transcription factor, the unique function of ZEB1 in PCC cells has been demonstrated [20]. Thus, we used *ZEB1* to label potential PCC cells in ST analysis. The results showed that a portion of ZEB1 + epithelial cells and IFIT1 + neutrophils co-located at the same spots (Fig. 5C, red box). Furthermore, IF staining was carried out on GC biopsy specimens (n = 64, 30 PCC cases and 34 NPCC cases) and quantified the level of infiltration of IFIT1 + TANs using integrated fluorescence intensity (IFIT1 and the typical neutrophil markers CD66b and CD55). We observed that PCC samples were associated with a higher level of IFIT1 + PDL1 + TANs infiltration compared to NPCC samples (Fig. 5D–F). Since the prognostic value of overall IFIT1 levels in GC has been previously demonstrated (Fig. 2P, Q), here we attempted to determine whether high infiltration of IFIT1 + TANs was associated with poor prognosis. As shown in Fig. 5G, IFIT1 + TANs was identified as a deleterious factor for PFI in GC patients. To further validate the results of ST analysis, the expression of IFIT1, ZEB1, and CD66b was analyzed by IF staining of the GC sample containing PCC components (> 50%, T6). As shown in Figs. 5H and S13, HE staining highlighted regions containing PCC components and regions not containing high proportion of PCC components. IF staining showed that high co-expression of IFIT1, CD66b, and ZEB1 was detected in the PCC region (IFIT1 + CD66b VS ZEB1, R = 0.8636, P < 0.0001), whereas these three showed only weak co-localization in the NPCC region (R = 0.1740, P < 0.0001). Many ZEB1 + cells were surrounded by the presence of a large infiltration of IFIT1 + neutrophils, indicating their physical proximity (direct interaction). To determine whether PCC or NPCC conditions could affect the phenotype of neutrophils, we cultured neutrophils with GC cell-conditioned medium (Fig. 5I, MKN45 and MKN74 cell lines, with or without upregulation of ZEB1 expression). We demonstrated an increase in neutrophil recruitment assessed by Transwell after incubation with MKN45 (especially in the oe-ZEB1 group), which mimiced the environment of PCC (Fig. 5J, P < 0.01). These data support the increased infiltration of neutrophils in PCC tissues, highest in MKN45 following treated with oe-ZEB1, which may reflect a unique immune mechanism of the PCC. Previous cell communication analysis suggested that PCC cells may act on neutrophils through stronger CXCL8-CXCR2 signaling. For this purpose, cell culture supernatants were collected and cytokine (CXCL8, CXCR2) concentrations were measured by ELISA assay. The results showed that MKN45 indeed exhibited stronger CXCL8-CXCR2 signaling compared to MKN74, and this effect was further enhanced by the upregulation of ZEB1, demonstrated by the higher levels of CXCL8/CXCR2 in the cell supernatants (Fig. 5K, L). In addition, IFIT1 and PDL1 expression was upregulated in neutrophils co-cultured with oe-ZEB1-treated GC cells and was particularly evident in the MKN45 cell line (Fig. 5M, N; PDL1, P < 0.0001; IFIT1, P < 0.01). Collectively, these results suggested that PCC cells possessed a greater ability to recruit and induce an immunosuppressive phenotype for TANs.

### PCC exhibits immunosuppressive features, which are associated with IFIT1 + TANs

**Fig. 6:**
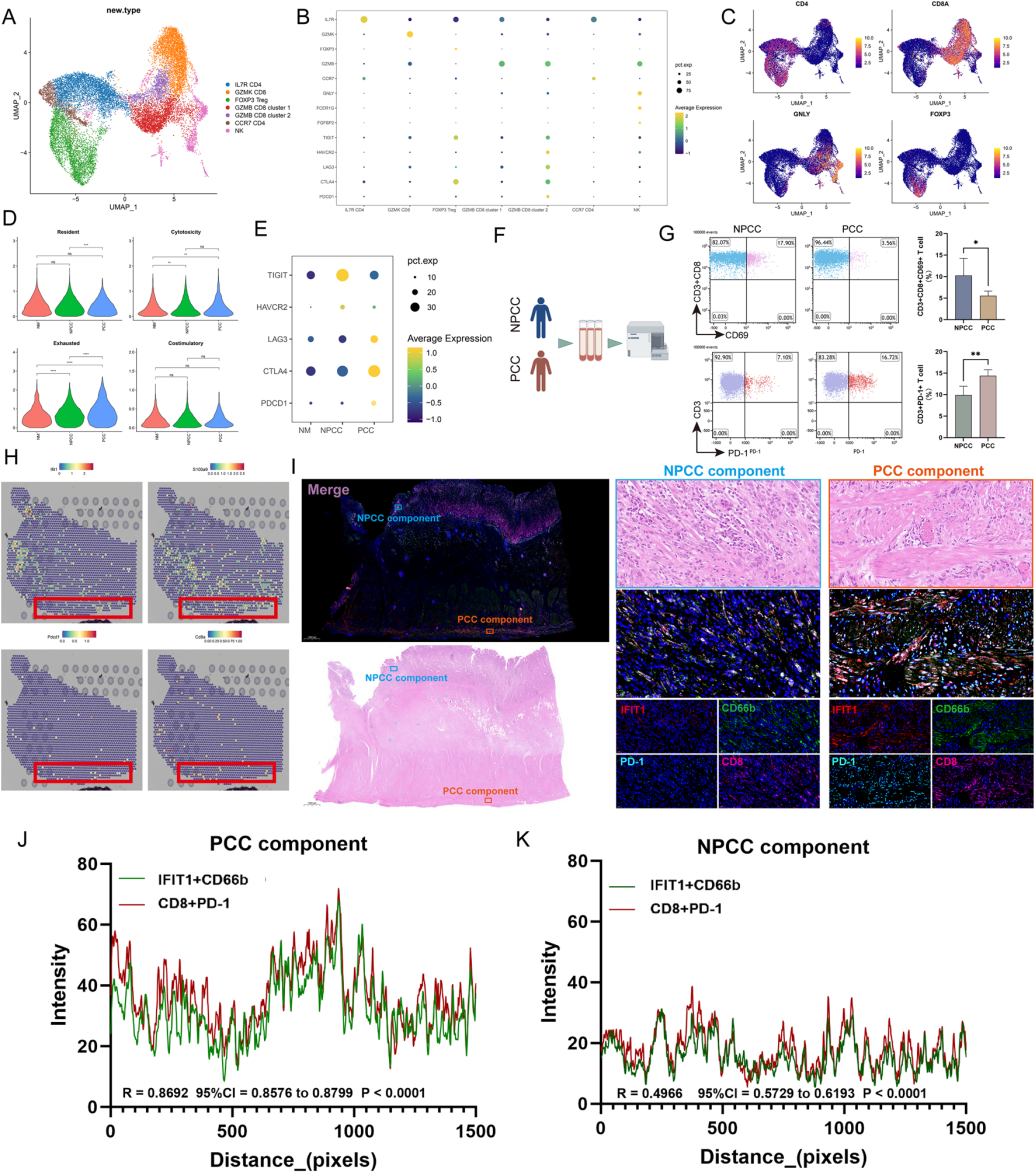
PCC exhibits IFIT1 + TAN-related immunosuppressive features. **A** Based on recognized markers, 10 clusters were annotated to seven T/NK cell lineages. See also Fig. S14A-B. **B** Bubble plot showing the expression of corresponding markers for different T/NK cell clusters. **C** UMAP plot showing the expression of *CD4*, *CD8A*, Granulysin (*GNLY*), and Forkhead Box P3 (*FOXP3*). **D** Violin plot showing the expression of resident, cytotoxic, exhausted, and stimulatory signature genes in CD8 + T cells in NM (red; N1, N2, N3), NPCC (green; T1, T2, T4, T5, T7, T8) and PCC (blue; T3, T6, T9) samples. Significance was determined by Wilcoxon rank-sum test. Ns: not significant, **P < 0.01, ***P < 0.001, ****P < 0.0001. See also Fig. S14C. **E** Bubble plot showing the expression of immune checkpoint genes in all T/NK cells in NM, NPCC, and PCC samples. **F** Flow chart of flow cytometry experiments. **G** Blood was collected from NPCC and PCC patients for flow cytometric analysis, and then labeled with CD3, CD8, and CD69. The box plots represent the ratio of activated and exhausted T cells for each group. T test was applied, *P < 0.05, **P < 0.01. See also Fig. S15. **H** Spatial transcription sections show the spatial expression of *IFIT1*, S100 Calcium Binding Protein A9 (*S100A9*) (Neutrophil marker), *PDCD1*, and *CD8A*. The dot color represents the expression level of the genes. **I** mIF staining of CD8, CD66b, IFIT1, and PD-1 in PCC samples. Representative regions are indicated by in the enlarged images at right, and the PCC components and NPCC components are distinguished by HE staining. Scale bars, 1000 μm (panoramic view) and 20 μm (partial view). **J**, **K** Co-localization of IFIT1 + neutrophils and PD1 + CD8 + T cells was quantified using the Spearman correlation coefficient in PCC components (J, R = 0.8692) and NPCC components (K, R = 0.4966)

Since high levels of IFIT1 + TANs in PCC samples suggest the activation of PD1/PDL1 pathway, the immunosuppressive features of PCC were further characterized here. We reclustered 19,344 T/NK cells and obtained 10 unique subpopulations (Fig. S14A, B), which were defined as 7 cell lineages by different marker genes, including 1 NK-cell cluster, 3 CD8 + T-cell clusters, 2 CD4 + T-cell clusters, and 1 Treg-cell cluster (Fig. 6A–C). As compared with NM and NPCC, the proportion of Treg cells (Fig. S14C) and the exhausted signature score of CD8 + T and NK cells (Fig. 6D, Wilcoxon rank-sum test, P < 0.0001) was higher in the PCC samples, while the resident score of CD8 + T and NK cells was significantly lower (Fig. 6D, Wilcoxon rank-sum test, P < 0.001). In addition, all T and NK cells from PCC samples exhibited higher levels of immune checkpoint genes, including *LAG3*, *CTLA4*, and *PDCD1*, compared to NM and NPCC (Fig. 6E). For experimental validation, we performed flow cytometry to explore the differences in immune profiles between NPCC and PCC patients (Figs. 6F and S15). We isolated T cells from the blood of NPCC and PCC patients, respectively, and examined the activation rate of CD8 + T cells and the proportion of all types of T cells expressing PD-1. The results showed that the activation rate of CD8 + T cells was significantly higher in the NPCC group, while the proportion of all types of T cells expressing PD-1 was higher in the PCC group (Fig. 6G, P < 0.001). Thus, our data confirmed that PCC patients possessed a stronger immunosuppressive TME.

ST analysis revealed spatial overlap of *IFIT1*, *PDCD1*, and *CD8A* (Fig. 6H). Considering that the test results of peripheral blood do not substantially reflect the characteristics of TME, we performed IF staining on PCC samples. The results showed the physical proximity of IFIT1 + neutrophils and PD1 + CD8 + T cells (Fig. 6I), supporting their direct interaction. Importantly, this phenomenon was more evident in the PCC regions (Fig. 6J, R = 0.8692) than in the NPCC regions (Fig. 6K, R = 0.4966), which was demonstrated by stronger CD8 + PD-1 and CD66b + IFIT1 co-localization of fluorescent signals. Together, these results suggested that the immunosuppressive features exhibited by PCC may be related to IFIT1 + TANs.

### IFIT1 + TAN induces SPP1 expressions from macrophages in PCC

**Fig. 7:**
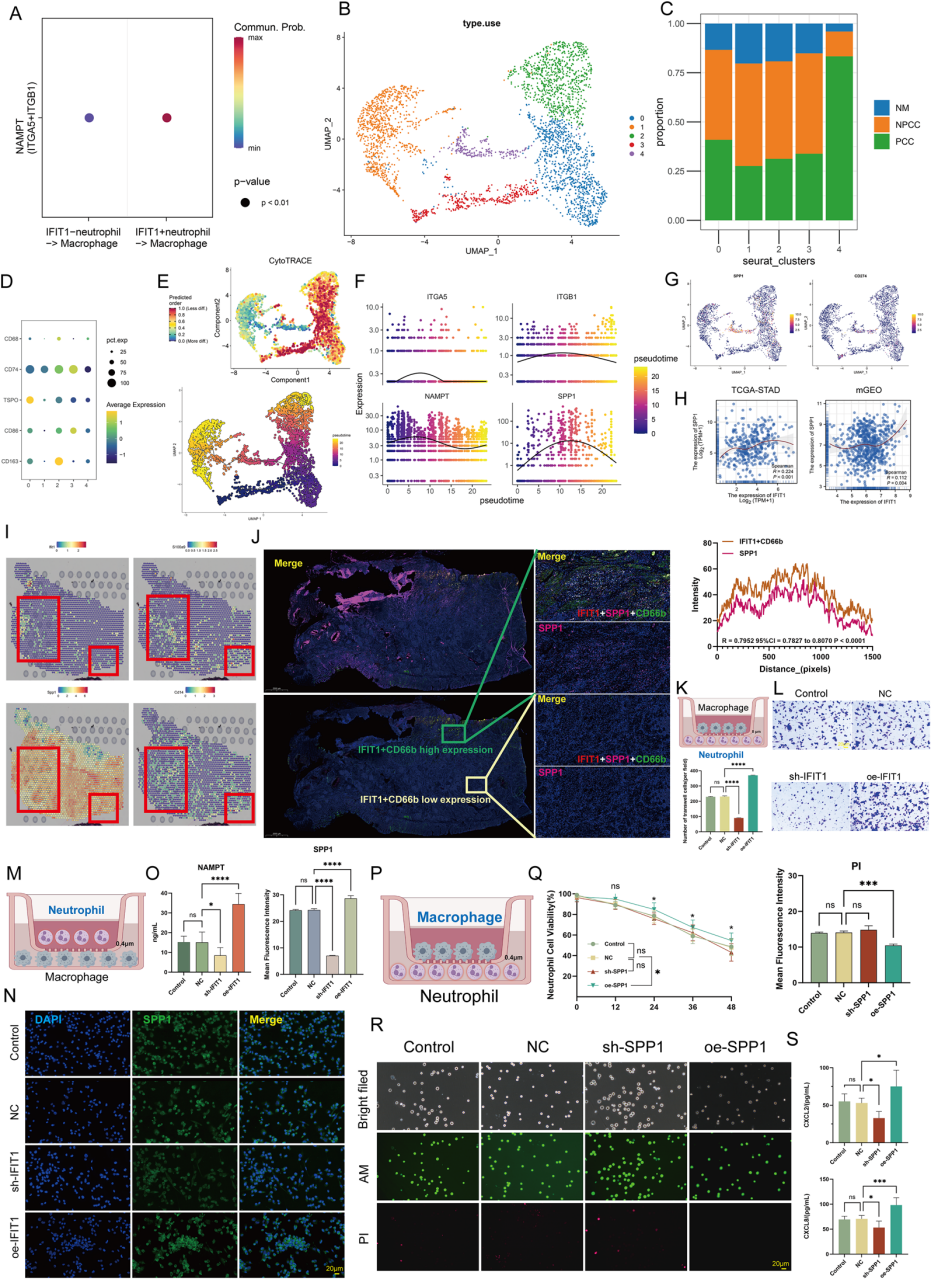
IFIT1 + TANs may promote SPP1 + macrophages infiltration through the Nicotinamide phosphoribosyltransferase (NAMPT) signaling pathway in PCC. **A** Bubble diagram showing that IFIT1 + TANs may communicate with SPP1 + macrophages more frequently through NAMPT signals. **B** UMAP plot of 3,388 macrophages, colourcoded for six molecular clusters. **C** Fraction of three tissue types in six cell subgroups. **D** Bubble plot showing the expression of macrophage marker genes for M1/M2 phenotype in all macrophages. **E** Trajectory of macrophages in constructed by “Monocle3”. Each point corresponds to a single cell and is colour-coded by pseudotime. **F** Expression patterns of NAMPT signals (Integrin Subunit Alpha 5, *ITGA5*; Integrin Subunit Beta 5, *ITGB5*; *NAMPT*) and Secreted Phosphoprotein 1 (*SPP1*) along the pseudo-temporal sequence. **G** UMAP plot showing co-expression of SPP1 and CD274 (PDL1). **H** Spearman correlation between *IFIT1* expression and *SPP1* expression as analyzed in TCGA-STAD (above) and mGEO (below). **I** Spatial transcription sections show the spatial expression of *IFIT1*, *S100A9*, *SPP1*, and *CD14* (macrophage marker). The dot color represents the expression level of the genes. **J** mIF staining of IFIT1, CD66b, and SPP1 in PCC samples. Representative regions are indicated by in the enlarged images at right, and the high/low level of IFIT1 + TANs regions are shown separately. Scale bars, 1000 μm (panoramic view) and 100 μm (partial view). Co-localization of IFIT1 + TANs and SPP1 was quantified using the Spearman correlation coefficient in the whole GC biopsy (R = 0.7952). **K** Schematic drawing of the co-culture model [macrophages (above) and neutrophils (below)]. **L** Transwell migration assay in neutrophils with the intervention of neutrophils over-/under-expressing IFIT1. Scale bars, 20 μm. ****P < 0.0001 vs. NC by t-test. **M** Schematic drawing of the co-culture model [neutrophils (above) and macrophages (below)]. **N** IF of SPP1 expression in macrophages with the intervention of neutrophils over-/under-expressing IFIT1. Scale bars, 20 μm. ****P < 0.0001 vs. respective control by t-test. **O** NAMPT ELISA assay in co-culture system with the over-expression/low-expression of IFIT1 in neutrophils. *P < 0.05, ****P < 0.0001 vs. NC by t-test. **P** Schematic drawing of the co-culture model [macrophages (above) and neutrophils (below)]. **Q** Line graph showing neutrophil viability with the intervention of macrophages over-/under-expressing SPP1. ns, not significant, *P < 0.05 vs. NC by t-test. **R** Calcein-AM/propidium iodide (PI) living/dead cell double staining in neutrophils with the intervention of macrophages over-/under-expressing SPP1. Scale bars, 20 μm. ns, not significant, ***P < 0.001 vs. NC by t-test. **S** CXCL2 and CXCL8 ELISA assays in co-culture system with the over-expression/low-expression of SPP1 in macrophages. ns, not significant, *P < 0.05, ***P < 0.001 vs. NC by t-test. The data represent three independent experiments

Neutrophils are usually recruited early to the site of inflammation, after which neutrophils enter an activated state, releasing Interleukin-8 (IL-8) and TNF-α, leading to intense macrophage infiltration [45]. This neutrophil-macrophage interaction is a widespread mechanism in the regulation of inflammation. In cancer, inflammation and fibrosis reinforce each other, conferring powerful invasiveness to tumor cells [46, 47]. Understanding tumor-associated inflammation can help reduce metastasis and improve antitumor therapy [48]. CellChat analysis showed that, compared to IFIT1- neutrophils, IFIT1 + neutrophils displayed stronger NAMPT signaling to macrophages (Fig. 7A). There is evidence that NAMPT is important for the differentiation of resting monocytes, polarizing them into M2 macrophages that inhibit inflammation and stimulate angiogenesis. We analyzed 3388 macrophages and classified them into 5 clusters based on the corresponding gene expression (Fig. 7B). Except for cluster 4, the other clusters were relatively evenly distributed across the different tissue samples (Fig. 7C). Importantly, we found that a high proportion of cluster-4 neutrophils originated from PCC samples. This suggested that macrophages, similar to neutrophils, had subpopulations that were tissue-specifically enriched, implying the presence of a PCC-associated neutrophil-macrophage regulatory mechanism. Among these macrophage clusters, cluster 0 showed M2-like features (high expression of CD163), while clusters 2 and 3 showed M1-like features (high expression of CD86) (Fig. 7D). To determine the evolutionary process within macrophages, we used the “Monocle3” and “CytoTRACE” tools to infer pseudotime trajectories and identify the path of macrophages from Cluster 3 to Cluster 1 (Fig. 7E, a continuous transition with a high CytoTRACE score cluster as the root). Interestingly, a recent study showed that macrophages infiltrated in the TME require SPP1 to maintain M2-like features, which in turn induces Tumor-associated macrophages (TAMs) to derive more SPP1, ultimately creating a positive feedback loop with pro-tumorigenic effects [49]. Based on previous results (Fig. 4), we focused on NAPMT signaling (ITGA5 + ITGB1) and SPP1 and found that they had similar gene expression trends along pseudotimes (Fig. 7F). In addition, co-localization of *SPP1* and *CD274* in cluster 4 was noted, further supporting the immunosuppressive phenotype (Fig. 7G). There is a positive association between TAM infiltration and upregulated PDL1, detected in approximately 40% of GC cases. Besides, we observed weak but significantly positive correlation between *IFIT1* and *SPP1* in the bulk data (Fig. 7H; above, TCGA-STAD, R = 0.224, P < 0.001; below, mGEO, R = 0.112, P = 0.004).

Next, we sought to investigate the regulatory role of IFIT1 + TANs on macrophages. Figure. 7I showed that IFIT1 + TANs and SPP1 + TAMs were in close spatial proximity, suggesting an interaction between these two cell types. To verify this physical proximity, IF labeling was used in PCC samples. As shown in Fig. 7J, we observed that SPP1 was strongly expressed in the high IFIT1 + TAN expression region, while the opposite result was found in the low IFIT1 + TAN expression region, further confirming the existence of potential crosstalk between these two cell types (IFIT1 + CD66b VS SPP1, R = 0.7952). The above evidence suggested that the spatial proximity between IFIT1 + TANs and TAMs may lead to a phenotypic shift in TAMs. Therefore, we established in vitro co-culture models to model the complex interactions that occur between these cells in vivo (Fig. 7K). To validate the results of the CellChat analysis (Fig. 4), we first examined macrophage mobility in the co-culture system, and IFIT1 was found to enhance the ability of neutrophils to recruit macrophages (Fig. 7L). Furthermore, in the co-culture model shown in Fig. 7M, upregulation of IFIT1 in TANs not only led to upregulation of SPP1 in TAMs (Fig. 7N, P < 0.0001) but also induced high levels of NAMPT (Fig. 7O, P < 0.05).

Considering that macrophages may also act on neutrophils, we further established a co-culture model as shown in Fig. 7P to detect this potential role. Interestingly, upregulation of SPP1 in macrophages significantly promoted neutrophil survival (Figs. 7Q, R, P < 0.05). More importantly, the co-culture system showed a simultaneous increase in the levels of CXCL2 and CXCL8 (Fig. 7S), both of which have been widely shown to promote neutrophil activation and migration [50, 51]. Altogether, these observations revealed the presence of the IFIT1 + neutrophil-SPP1 + macrophage axis.

### IFIT1 + TANs promote stromal cell activation

**Fig. 8:**
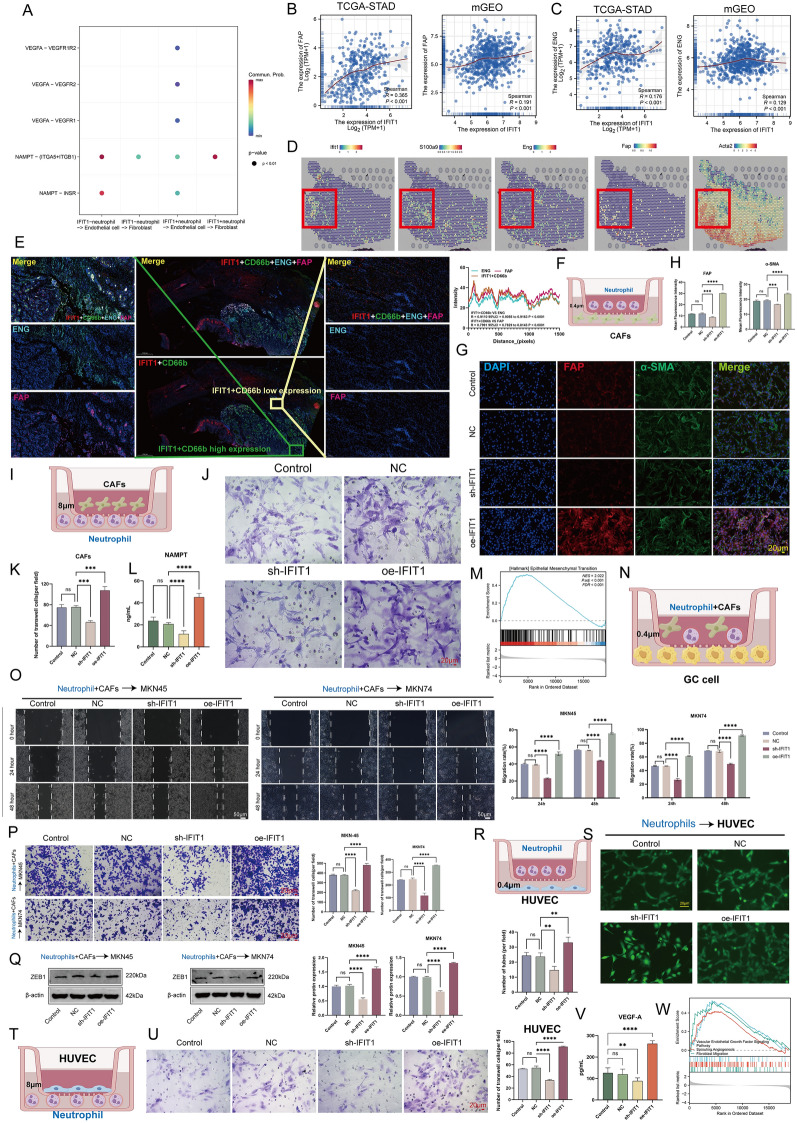
IFIT1 + TANs may promote stromal cell activation through the NAMPT and VEGF signaling pathway in PCC. **A** Bubble diagram showing that IFIT1 + TANs may communicate with fibroblast (through NAMPT signals) and endothelial cells (through VEGF signals) more frequently. **B**, **C** Spearman correlation between *IFIT1* expression and Fibroblast Activation Protein Alpha (*FAP)* (fibroblast marker) expression (**B**) as well as Endoglin (*ENG*) (endothelial marker) expression (**C**) in TCGA-STAD and mGEO. **D** Spatial transcription sections show the spatial expression of *IFIT1*, *S100A9*, *ENG*, *FAP*, and *ACTA2*. The dot color represents the expression level of the genes. **E** mIF staining of IFIT1, CD66b, ENG, and FAP in PCC samples. Representative regions are indicated by in the enlarged images at left (high IFIT1 + TANs level region) and right (low IFIT1 + TANs level region). Scale bars, 1000 μm (panoramic view) and 100 μm (partial view). Co-localization of IFIT1 + TANs and ENG/FAP was quantified using the Spearman correlation coefficient in the whole GC biopsy (R = 0.9110 for IFIT1 + CD66b VS ENG, R = 0.7991 for IFIT1 + CD66b VS FAP). **F** Schematic drawing of the co-culture model [neutrophils (above) and cancer-associated fibroblasts (CAFs, below)]. **G**, **H** mIF of FAP and α-SMA expression in CAFs with the intervention of neutrophils over-/under-expressing IFIT1. Scale bars, 20 μm. **P < 0.01, ****P < 0.0001 vs. respective control by t-test. **I** Schematic drawing of the co-culture model [CAFs (above) and neutrophils (below)]. **J**, **K** Transwell migration assays in CAFs with the intervention of neutrophils over-/under-expressing IFIT1. Scale bars, 20 μm. ***P < 0.001 vs. respective control by t-test. **L** ELISA assay of NAMPT in co-culture system with the over-expression/low-expression of IFIT1 in neutrophils. ****P < 0.0001 vs. NC by t-test. **M** Gene set enrichment analysis (GSEA) showing a positive correlation between IFIT1 expression and the EMT pathway (background gene set: HALLMARK). **N** Schematic drawing of the co-culture model [neutrophils and CAFs(above), and tumor cells (MKN45 and MKN74, below)]. **O**, **P** Wound-healing (**O**) and invasion assay (**P**) in MKN45 and MKN74 cells with the intervention of neutrophils over-/under-expressing IFIT1, which were direct co-cultured with CAFs. Scale bars, 50 μm. ****P < 0.0001 vs. NC by t-test. **Q** Western blotting of ZEB1 in MKN45 and MKN74 cells in co-culture system which is described in **N**. Scale bars, 50 μm. ****P < 0.0001 vs. NC by t-test. **R** Schematic drawing of the co-culture model [neutrophils (above) and Human umbilical vein endothelial cells (Human umbilical vein endothelial cells, HUVECs below)]. **S** Tubule formation assay in HUVECs with the intervention of neutrophils over-/under-expressing IFIT1. Scale bars, 20 μm. **P < 0.01 vs. NC by t-test. **T** Schematic drawing of the co-culture model [HUVECs (above) and neutrophils (below)]. **U** Transwell migration assays in HUVECs with the intervention of neutrophils over-/under-expressing IFIT1. Scale bars, 20 μm. ****P < 0.0001 vs. NC by t-test. **V** ELISA assay of VEGF-A in co-culture system with the over-expression/low-expression of IFIT1 in neutrophils. **P < 0.01, ****P < 0.0001 vs. NC by t-test. **W** GSEA showing a positive correlation between IFIT1 expression and Vascular Endothelial Growth Factor Signaling Pathway, Sprouting Angiogenesis, and Fibroblast Migration (background gene set: Gene Ontology). The data represent three independent experiments

Our previous study demonstrated the presence of a large number of myofibroblasts and abundant blood vessels in the PCC microenvironment [20]. Here we attempted to further parse the underlying biological mechanisms. On the basis of the above results (Fig. 4G), CellChat analysis showed that IFIT1 + neutrophils may frequently communicate with fibroblasts and endothelial cells through NAMPT signaling, and VEGF signaling, respectively (Fig. 8A). Correlation scatter plots were generated based on bulk data to investigate the overall correlation between *IFIT1* and the fibroblast marker Fibroblast Activation Protein Alpha (*FAP*) as well as the endothelial cell marker Endoglin (*ENG*). As shown in Figs. 8B, C, there was a significant but weak positive correlation between the levels of *IFIT1* and *FAP* (Fig. 8B; left, TCGA-STAD, R = 0.365, P < 0.001; right, mGEO, R = 0.191, P < 0.001); the levels of *IFIT1* and *ENG* were positively correlated as well (Fig. 8C; left, TCGA-STAD, R = 0.176, P < 0.001; right, mGEO, R = 0.129, P < 0.001). ST analysis further highlighted their physical proximity (Fig. 8D). IF staining showed high expression of ENG and FAP around IFIT1 + neutrophils and low expression around IFIT1- neutrophils (Fig. 8E; IFIT1 + CD66b VS ENG, R = 0.9110; IFIT1 + CD66b VS FAP, R = 0.7991). The results suggested that over-infiltration of IFIT1 + neutrophils was closely related to remodeling of the stroma.

Next, we sought to clarify the action of IFIT1 + TANs on fibroblasts. Therefore, we co-cultured neutrophils with CAFs for 24 h (Fig. 8F). CAFs were then assayed for expression of activation markers. CAFs from co-cultures with oe-*IFIT1* TANs expressed higher levels of FAP and α-SMA compared with NC/sh-*IFIT1* TANs, confirming that IFIT1 + TANs possessed the ability to promote fibroblast activation (Fig. 8G-H, P < 0.001). In addition, the ability of IFIT1 + TANs to recruit CAFs was tested (Fig. 8I). As shown in Fig. 8J–L, we found that upregulation of IFIT1 expressed by TANs resulted in a significant increase in CAF recruitment, whereas the opposite result was observed in the sh-*IFIT1* group (P < 0.001). Enrichment analysis showed that IFIT1 may be involved in the EMT process (Fig. 8M, TCGA-STAD, adjust P < 0.001). There are strong evidences that CAFs are in direct or indirect contact with pre-EMT tumor cells and emit pro-EMT signals from these stromal cells to promote tumor cell EMT [52]. Therefore, a co-culture model as shown in Fig. 8N was established to detect the effect of the TAN-CAF axis on tumor cells. Specifically, MKN45 and MKN74 cells were cultured in conditioned media generated using the TANs and CAFs co-culture system, and then the invasive ability of GC cells was examined under different conditions (Control, NC, sh-*IFIT1*, and oe-*IFIT1*). Wound healing (Fig. 8O) and transwell (Fig. 8P) assays demonstrated that treatment with oe-*IFIT1* promoted cell migration and invasion in MKN45 and MKN74 cells; however, the sh-IFIT1 group showed the opposite phenomenon (P < 0.0001). Importantly, WB assays showed that IFIT1 overexpression (TANs) significantly upregulated ZEB1 protein expression in MAK45 and MKN74 cells (Fig. 8Q, P < 0.0001). Previously, we demonstrated that ZEB1 overexpressing GC cells promoted IFIT1 expression in TANs, suggesting the existence of a positive feedback loop between IFIT1 + TANs and ZEB1 + GC cells.

Finally, we investigated the effects of TANs on vascular endothelial cells in two co-culture systems. Tube formation (Fig. 8R, S) and Transwell (Fig. 8T, U) assays confirmed that HUVECs co-cultured with oe-*IFIT1*-treated TANs showed stronger migration and higher tube counts compared with the other groups (P < 0.01). To validate the results of the CellChat analysis (Fig. 4J), we also examined the levels of VEGFA in the culture system. As shown in Fig. 8V, upregulation of IFIT1 induced high levels of VEGFA (P < 0.0001). Enrichment analysis summarized IFIT1-associated stromal signals including VEGF Signaling Pathway, Sprouting Angiogenesis, and Fibroblast Migration (Fig. 8W, adjust P < 0.001). Accordingly, these data suggested that IFIT1 + TANs regulated stromal cells activation.

### Overview of IFIT1 in human cancers

Considering the lack of IFIT1-related cancer research, we evaluated *IFIT1* expression at the pan-cancer level. The results showed that *IFIT1* expression was downregulated in seven cancers, including Colon adenocarcinoma (COAD), Kidney chromophobe (KICH), Kidney renal papillary cell carcinoma (KIRP), Lung adenocarcinoma (LUAD), Lung squamous cell carcinoma (LUSC), Rectum adenocarcinoma (READ), and Uterine corpus endometrial carcinoma (UCEC), compared with normal tissues (Fig. S16A, P < 0.05). Proteomics data further supported these results (Fig. S16B). And as expected, the transcribed mRNA and translated protein levels of IFIT1 were positively correlated in 10 available cancers (Fig. S16C, linkedomicsKB), suggesting that IFIT1 expression may be primarily regulated by pre-transcriptional modifications. To characterize the relationship between *IFIT1* expression and intertumoral biological signaling, correlations between *IFIT1* in pan-cancer data and well-known molecular signatures (HALLMARK database) in pan-cancer dataset were calculated. In most cancers, upregulated *IFIT1* was found to correlate with interferon α response level, interferon γ response level, EMT level, angiogenesis level, pi3k akt mtor signaling level, and mtorc1 signaling level (Fig. S16D). Considering the close association of interferon response and adaptive immunity, we employed TIMER, CIBERSORT, CIBERSORT-ABS, QUANTISEQ, MCPCOUNTER, and EPIC algorithms for further immune infiltration estimation. As shown in Fig. S16E-J, we observed a relatively significant correlation between *IFIT1* expression and the level of immune infiltration of neutrophils, monocytes/macrophages, dendritic cells, and Treg cells in most cancers. Furthermore, we found that *IFIT1* was positively correlated with immune checkpoint gens such as *PDCD1*, *CD274*, *CTLA4*, *TIGIT*, *LAG3*, *HAVCR2*, and Programmed Cell Death 1 Ligand 2 (*PDCD1LG2*) in cancers other than Mesothelioma (MESO), Liver hepatocellular carcinoma (LIHC), Kidney renal papillary cell carcinoma (KIRP), KIRC, Cholangiocarcinoma (CHOL), and Adrenocortical carcinoma (ACC) (Fig. S16K). Concurrently, using univariate Cox regression analysis, the prognostic value of *IFIT1* expression for OS was validated in an independent TCGA cancer cohort containing 9163 tumor samples (Fig. S16L). *IFIT1* expression was supported as a prognostic biomarker in five independent TCGA cohorts including KIRC, KIRP, Skin cutaneous melanoma (SKCM), STAD, and UCEC. Notably, unlike in STAD and UCEC (HR > 1), *IFIT1* was a positive prognostic factor in tumors with high immunogenicity, TMB, and immune infiltration, such as SKCM, KIRC, KIRP (HR < 1). Since the correlation between *IFIT1* and neutrophils is broad, we obtained ST data for pan-cancer. As shown in Fig. S16M, ST analysis depicted the spatial overlap of *IFIT1* and neutrophil biomarkers *S100A9,* and *PDL1* on Cervical cancer (CESC), Glioblastoma Multiforme (GBM), Renal cell carcinoma (RCC), Breast invasive carcinoma (BRCA), and Bladder urothelial carcinoma (BLCA) cancer tissues. These results supported the idea that IFIT1 was a key factor in the construction of TME in many cancers, possibly by suppressing immunostimulatory functions and immune checkpoint effects.

### IFIT1 + TANs exert pro-tumorigenic functions in vivo and mediate the resistance to immunotherapy via IFN-γ

The above pan-cancer analysis further showed the importance of IFIT1 in TME, and since immunosuppressive TME is a major obstacle to immunotherapy, we next investigated the effect of IFIT1 + TANs on immunotherapy response in in vivo models.

First, the ability of IFIT1 + TANs to promote tumor growth in vivo was tested in a xenograft tumor model. As shown in Fig. S17A, IFIT1 up-/down-regulated TANs or control cells were mixed with tumor cells (MKN45) and the mixture was injected into the subcutaneous tissue of BALB/c nude mice. We found that upregulation of IFIT1 resulted in a significant increase in tumor growth; however, this effect was reversed in mice carrying tumors with sh-IFIT1 (Fig. S17B-D), confirming that IFIT1 promoted tumor growth through in vivo regulation of neutrophils. ZEB1 and PDL1 within the xenograft tumor tissues were labeled by IF staining, as shown in Fig. S17E-F, IFIT1 overexpression (TANs) significantly promoted the expression of ZEB1 and PDL1 (P < 0.0001), and NAMPT and VEGF signaling levels were altered accordingly (P < 0.0001), further supporting the conclusions of our in vitro experiments.

**Fig. 9:**
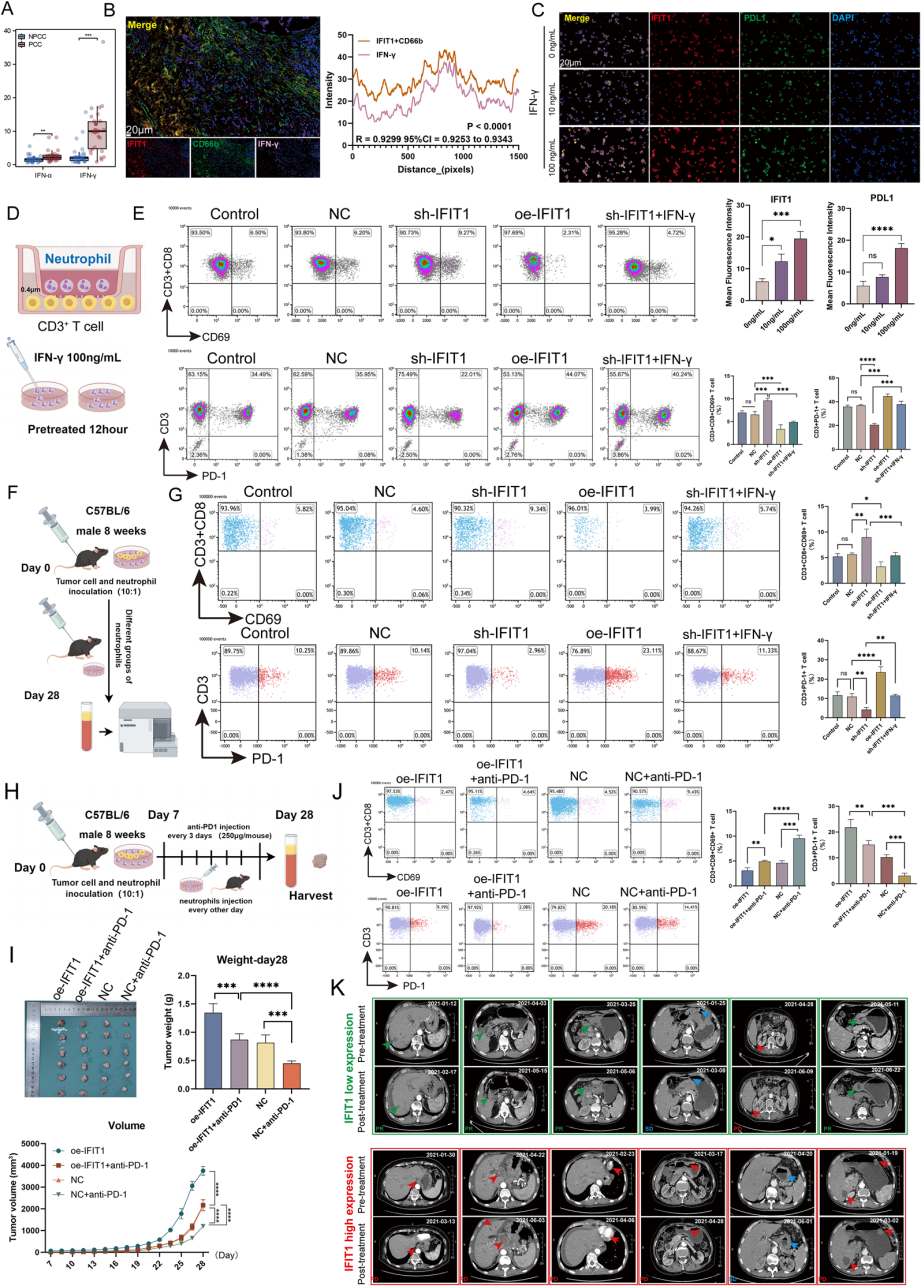
The tumor promotion functions of IFIT1 in vivo and the adverse effects of IFIT1 on immunotherapy. See also Fig. S16-S17. **A** Box plots showing interferon-alpha/gamma (IFN-α/γ) levels in PCC and NPCC serum samples (before surgery, n = 64, 30 PCC cases and 34 NPCC cases). **P < 0.01, ***P < 0.001 vs. respective control by t-test. See also Fig. S18. **B** mIF staining of IFIT1, CD66b, and IFN-γ in GC samples. Scale bars, 20 μm. Co-localization of IFIT1 + TANs and IFN-γ was quantified using the Spearman correlation coefficient in the whole GC biopsy (R = 0.9299). **C** In vivo experiments, mIF of ZEB1 and PDL1 expression after stimulation with IFN-γ. Scale bars, 20 μm. *P < 0.05, ***P < 0.001, ****P < 0.0001 vs. respective control by t test. **D** Schematic drawing of the co-culture model [neutrophils (above) and CD3 + T cells (below)] with or without addition of human IFNγ. **E** The activation and exhaustion phenotypes of tumor-infiltrating lymphocytes (TILs) assessed using flow cytometry and the statistical analysis of the results. ns, not significant, ***P < 0.001, ****P < 0.0001. See also Fig. S19A. **F** Protocol schematic of the combination of IFIT1 interferences and IFNγ treatment for mice implanted subcutaneously with MFC cells. **G** The activation and exhaustion phenotypes of tumor-infiltrating lymphocytes (TILs) assessed using flow cytometry and the statistical analysis of the results (n = 6). *P < 0.05, **P < 0.01, ***P < 0.001, ****P < 0.0001. See also Fig. S19B. **H** Protocol schematic of the combination of IFIT1 interferences and anti-PDL1 therapy for mice implanted subcutaneously with MFC cells. **I** Photographs, weights, and volumes of tumors removed from mice treated with the oe-IFIT1, anti-PDL1, their combination, or NC (n = 6). ***P < 0.001, ****P < 0.0001. **J** The activation and exhaustion phenotypes of tumor-infiltrating lymphocytes (TILs) assessed using flow cytometry and the statistical analysis of the results (n = 6). **P < 0.01, ***P < 0.001, ****P < 0.0001. See also Fig. S19B. **K** Representative CT images during immunotherapy for GC patients (n = 12) with low IFIT1 levels (**R**) and high IFIT1 levels (**S**) at baseline (H score). The arrows indicate the primary or metastatic tumor foci. Red for progressive disease (PD), Green for partial response (PR), Blue for stable disease (SD). See also Figs. S20 and S21. The data represent three independent experiments

The results of enrichment analysis suggested an important role for *IFIT1* in the interferon response. And *IFIT1*, as one of the Interferon-stimulated genes (ISGs), was strongly induced by type I interferons (IFN-α and IFN-β), double-stranded RNA and viral infection [53]. Since interferons directly regulate the transcription of a large number of downstream genes, this leads to a myriad of direct (on cancer cells) and indirect (through immune and stromal components) effects on tumors [54, 55]. We hypothesized that this interferon-related response may be one of the main reasons why IFIT1 affects TME. To this end, we focused on the significance of the interferon pathway for IFIT1-PDL1 signaling in GC. Cytokine assay data from 12 GC patients were utilized. Interestingly, we observed that IFN-α and IFN-γ showed consistent upregulation in serum samples from PCC patients (Fig. 9A, P < 0.01), which was also confirmed by IF staining in GC biopsy specimen (Fig. 9B, PCC-specific IFIT1 + TANs are surrounded by large amounts of INF-γ). This implied a potential role for interferon responses in IFIT1-PDL1 signaling. To further determine which of the two interferon responses is more important, we extracted transcriptional profiling data from TCGA-STAD and mGEO data and clustered these molecules using a hierarchical clustering method with Manhattan distance. We found that these 16 molecules can be visually divided into 2 clusters, where *IFIT1*, *CD274*, and *IFNG* were clustered together because of their similar expression profiles (Fig. S18A-D). More importantly, in the single-cell data, IFIT1 + neutrophils expressed much higher levels of *IFNG* than IFIT1- neutrophils (Fig. S18E). Further comparing the mRNA levels of interferon-coding genes in the two previously identified modification patterns, as shown in Fig. S18F, *IFNG* exhibited upregulation in C2 from both independent datasets (upper part, TCGA-STAD, P < 0.001; bottom part, mGEO, P < 0.05). These results suggested that *IFNG* may play an important function in IFIT1 + TAN-related effector mechanisms. Extensive studies have shown that IFN-γ is a key driver of PD-L1 expression in tumor and host cells [56, 57]. In addition, a previous study showed that spleen-derived IFN-γ induced the production of PD-L1 + -inhibitory neutrophils thereby playing a key role in immunosuppression [58]. Based on these facts, we hypothesized that IFN-γ may exert an immunosuppressive function in GC, especially in PCC, by inducing the expression of IFIT1 and PDL1. To clarify this, we further investigated the role of IFN-γ signaling in inhibitory neutrophil production by stimulating freshly isolated neutrophils with IFN-γ. As shown in Fig. 9C, IFN-γ, especially at high doses, was confirmed to increase the expression of IFIT1 and PDL1 by in vitro experiments (Fig. 9C, P < 0.05).

We next experimentally verified the effect of IFIT1 on anti-tumor immunity. Firstly, in the in vitro model, we co-cultured neutrophils with different treatments and T cells for 24h (Fig. 9D) and then performed flow cytometry to detect the phenotypes of T cells. Flow cytometry showed that the activation percentage of T cells (CD3 + lymphocyte) in the sh-IFIT1 group was significantly higher than that of IFN-γ in the control and sh-IFIT1 + groups (Figs. 9E and S19A, P < 0.001), whereas the exhaustion percentage showed the opposite trend (Fig. 9E, P < 0.001). T cell is a main component of adaptive antitumor immunity. We thus investigated the infiltration of T lymphocytes in IFIT1 + neutrophil-MFC tumors in an in vivo model. For this purpose, we implanted a mixture of differently treated neutrophils and tumor cells into immunocompetent C57BL/6 mice (Fig. 9F). As expected, flow cytometry showed similar results to the in vitro experiments (Figs. 9G and S19B, P < 0.05). These results further supported the influence of IFIT1 + neutrophils on T-cell immunity, especially in the context of IFN-γ stimulation. To determine the effect of IFIT1 + neutrophils on the responses of immunotherapy, we administered oe-IFIT1 vector alone or in combination with anti-PD1 in the above tumor models (Fig. 9H). We observed a significant increase in tumor size, tumor volume, and tumor weight in the oe-IFIT1 group compared to the control group, and these increases were significantly attenuated in the combined anti-PD1 group (Fig. 9I, P < 0.001). Based on flow cytometry analysis, anti-PD1 treatment significantly promoted the activation but suppressed the exhaustion phenotype of tumor-infiltrating cytotoxic CD8 + T cells in mice compared to controls, and these effects were reversed by oe-IFIT1 (Fig. 9J, P < 0.01). These results confirm that IFIT1 + neutrophils impair effective immunotherapy in malignant tumors.

Clinical evidence suggests that IFN-γ associated transcriptional profiles predict clinical response rates to PD-1 blockade [59]. Thus, we further evaluated whether IFIT1 could serve as an immunotherapy predictor for GC patients. We calculated the Tumor Immune Dysfunction and Exclusion (TIDE) score to predict the response to immune checkpoint blockade in GC patients in the TCGA-STAD cohort. The results showed that IFIT1 expression correlated with TIDE score and dysfunction score (Fig. S20A, P < 0.01). Since higher TIDE scores indicate that patients are less likely to benefit from immunotherapy, we hypothesized that IFIT1 exerts an immunosuppressive effect that may be more dependent on T cell dysfunction-related pathways. To this end, we examined the relationship between *IFIT1* expression and response to immunotherapy from a dataset (PRJEB25780 cohort) containing 45 GC patients treated with anti-PD1. As shown in Fig. S20B, patients who were responsive had lower *IFIT1* expression than those who were not responsive (P < 0.01). To validate these results, we evaluated the relationship between immunotherapy response and IFIT1 expression by CT imaging in our cohort (n = 12). Tumor volume changes recorded by computed tomography (CT) in all cases were shown (Fig. 9K). The results showed that, among all patients, IFIT1 high-expression cases exhibited a higher rate of acquired immunotherapy resistance than IFIT1 low-expression cases. We also performed an analysis using another single-cell data cohort containing 32 melanoma patients treated with anti-PD1 (Fig. S21A-D) and found that in S100A9 + myeloid cell (Mye_C6_S100A9), the proportion of cells from responsive patients (87.25%) was much lower than that of cells from non-responsive patients (12.75%) (Fig. S21E). More importantly, *IFIT1* was expressed in a much higher proportion of non-responsive cells than in responsive cells (Fig. S12F). These data strengthened the evidence that *IFIT1* was a deleterious factor for immunotherapy.

## Discussion

As a highly phenotypically and molecularly heterogeneous disease, the intra-tumor heterogeneity of GC is a major obstacle to precision therapy [60, 61]. PCC is a distinct histological subtype of GC that tends to metastasize and often results in low survival [62]. Currently, immunotherapy targeting the PD-1/PDL1 pathway is one of the most effective therapeutic options, but its efficacy in PCC is unsatisfactory, partly due to the highly immunosuppressive state of PCC [63]. In addition, patients with PCC are prone to develop resistance to immunotherapy and chemotherapy, thus failing to sustain benefit [64, 65]. Therefore, new knowledge is urgently needed to help understand the unique molecular mechanisms of PCC and to develop new effective therapeutic agents for PCC.

Immunosuppressive TME is a major barrier to immunotherapy [66]. Tumors can acquire an immunosuppressive phenotype through the expression of PDL1, CTLA4, and other immunosuppressive proteins to suppress innate and adaptive immune function, leading to immune escape of cancer cells [67]. Binding of major histocompatibility complex (MHC)-presented immunogenic peptide antigens to heterodimeric T cell receptors (TCRs) is required for T cell activation [68]. Binding of PD1 to its ligand, PDL1, inhibits signaling downstream of TCRs, thereby inducing T cell apoptosis and exhaustion [68]. In this study, we noted that PDL1 was the only immune checkpoint gene highly expressed in PCC samples. A transcriptomic study in breast cancer showed a significant correlation between EMT scores and PDL1 mRNA levels [69]. More importantly, this correlation was particularly evident in claudin-low subtype breast cancers, suggesting a bidirectional interference between PDL1 and mesenchymal phenotype [69]. Our preliminary results demonstrated that PCC exhibited low expression of TJ proteins (claudins), the loss of which is an early step in the EMT process [70]. We therefore hypothesized that a similar crosstalk mechanism may also exist in PCC.

Leading our interest, PCC samples had a strong neutrophil infiltration. Notably, a TAN population enriched in myeloid cell-enriched subtypes was associated with a poor prognosis of hepatocellular carcinoma (HCC) [70]. Specifically, TANs were able to recruit macrophages and inhibit T-cell toxicity [47]. In this study, a population of neutrophils with PCC specificity (IFIT1 + TANs) was labeled by module 3 defined by hdWGCNA. We characterized two distinct GC subtypes (C1 and C2) based on the expression profiles of module 3 member genes. C2 had a poorer OS compared to C1. Importantly, the immune infiltration algorithms showed that the high infiltration of IFIT1 + neutrophils may lead to the activation of pro-tumorigenic cells including M2 macrophages, fibroblasts, and vascular endothelial cells. Further enrichment analysis showed that C2 activated mesenchymal cell activation-associated signals and was enriched for EMT signals, and activation of these mesenchymal signals in gastric GC may be associated with poor prognosis. Investigators reported that solid tumors can be classified into immune-inflammatory, immune-desert, and immune-exclusion types based on TME characteristics [71]. Based on our results, C2 may be more inclined to be an immune-exclusion type tumor, and the high level of immunosuppressive cell infiltration may make it difficult for C2 to benefit from immunotherapy. Notably, all SRC samples from two independent cohorts were included in C2. We speculate that high levels of infiltration with IFIT1 + TANs may be one of the important features of PCC.

Tumor cells are known to promote tumor progression by educating the inflammatory microenvironment [72]. We found here that tumor cells educate neutrophils in PCC via CXCL signaling to promote their PDL1 expression. Our previous work demonstrated that the EMT transcription factor ZEB1 is highly expressed in PCC, helping the tumors to maintain mesenchymal characteristics [20]. On this basis, we demonstrated that local ZEB1 expression in tumor tissues promoted IFIT1 + TANs infiltration. Mesenchymal cell activation contributes to the tumor stroma promoting tumor cell invasiveness and metastasis, and impedes T and NK cell infiltration thereby inhibiting tumor-killing effects [73, 74]. In the present study, we found that IFIT1 + neutrophils communicated frequently with macrophages and fibroblast cells via NAMPT signaling and with endothelial cells via VEGF signaling in PCC tumors. In vitro experiments confirmed that IFIT1 + TANs have a potent ability to confer an immunosuppressive phenotype on mesenchymal cells. These facts reveal a complex signaling crosstalk in the PCC tumor microecology, but key among them may be the ZEB1 + tumor cells and IFIT1 + TANs. since ZEB1 is one of the most frequently activated signals in human cancers [75, 76], whether malignant crosstalk between these two types of cells triggers additional downstream pathways deserves further exploration.

Strong expression of PD-L1 and IFN-γ has been shown to characterize GC patients with poor prognosis and is accompanied by a high proportion of activated CD4 + T cells and fibroblasts infiltrating in the TME [77]. PDL1 results from endogenous pro-tumorigenic signaling in tumor cells and adaptive immune resistance, the latter initially maintaining immune homeostasis and preventing autoimmunity [78, 79]. A growing number of studies point to this negative feedback mechanism of the immune system as playing an important role in PDL1 expression in TME [80, 81]. As a major CD4 + T-cell effector cytokine, IFN-γ is secreted in large quantities in localized regions of the tumor and can subsequently promote the transcription of PD-L1 in both tumor and immune cells in TME [82, 83]. In the present study, we reported that IFIT1 + TANs were stimulated by exogenous IFN-γ and reduced the number and function of tumor-infiltrating activated T cells. Meanwhile, IFIT1 + TANs infiltration might decrease the efficacy of PD1 blockade. Therefore, IFIT1 + TANs could represent a therapeutic target in PCC.

We recognize that this study also has some limitations. First, part of this study was a retrospective study based on publicly available data, and correction for batch effects may have resulted in some of the evidence appearing weak due to unknown effects. We are collecting patients in a multicenter clinical cohort for further protein sequencing analysis to validate our conclusions. Second, our findings suggest that IFIT1 + TANs may play an important role in PCC, but their complex mechanisms in TME, especially signaling with other cells, are yet to be investigated. Our lab is trying to delve deeper into this direction. Finally, since immune infiltration and its effects on TME are influenced by intratumoral heterogeneity, just as different regions of the tumor contain different proportions of PCC tissue, it is appropriate to assess regional heterogeneity accordingly. Although we overcame the difficulties posed by this heterogeneity to a large extent using immunofluorescence and spatial transcriptomic data, a large number of calculations were derived from bulk data (where it was not possible to take into account positional information), and thus further large-scale analyses of spatial-omics could help to fully resolve the spatial heterogeneity of PCC.

## Conclusion

Taken together, our work provides evidence for the function of IFIT1 + TANs in PCC tumors and suggests that IFIT1 + TANs and ZEB1 + tumor cells play an important role in TME remodeling through multiple mechanisms of signaling crosstalk. These results improve our understanding of PCC, introduce combined IFIT1 and PDL1 as a promising target for anti-PCC precision therapy, and provide clues for the inefficacy/resistance of PCC to immunotherapy.

### Supplementary Information

Supplementary Material 1.

## Data Availability

The data and materials in the current study are available from the corresponding author Xi Zou (fsyy00670@njucm.edu.cn).

## Abbreviations

IFIT1: Interferon induced protein with tetratricopeptide repeats 1
TANs: Tumor-associated neutrophils
TME: Tumor microenvironment
GC: Gastric cancer
PDL1: Programmed cell death 1 ligand
SPP1: Secreted phosphoprotein 1
PCC: Poorly cohesive carcinoma
WHO: World Health Organization
MP: Mesenchymal phenotype (MP) and
EP: Epithelial phenotype
TCGA: The Cancer Genome Atlas
ACRG: Asian Cancer Research Group
EMT: Epithelial mesenchymal transition
NOS: Not-otherwise-specified
SRC: Signet cell carcinoma
ZEB1: E-Box binding homeobox 1
STAD: Stomach adenocarcinoma
GEO: Gene expression omnibus
DEGs: Differentially expressed genes
GSCA: Gene set cancer analysis
scRNA: Single cell RNA
GO: Gene ontology
KEGG: Kyoto Encyclopedia of Genomes
hdWGCNA: High dimensional weighted gene co-expression network analysis
ST: Spatial transcriptomics
TNM: Tumor-node-metastasis
NM: Normal mucosa
PBS: Phosphate-buffered saline
DMEM: Dulbecco’s modified Eagles medium
PCA: Principal component analysis
UMAP: Uniform manifold approximation and projection
IHC: Immunohistochemistry
HE: Hematoxylin/eosin
CAFs: Cancer-associated fibroblasts
HUVECs: Human umbilical vein endothelial cells
ATCC: American type culture collection
FBS: Fetal bovine serum
STR: Short tandem repeat
SEM: Standard error of the mean
RIPA: Radioimmunoprecipitation
WB: Western blotting
ELISA: Enzyme-linked immunosorbent assay
AVMA: American Veterinary Medical Association
CNV: Copy number variation
FGF: Fibroblast growth factor
TAMs: Tumor-associated macrophages
COAD: Colon adenocarcinoma
KICH: Kidney chromophobe
LUAD: Lung adenocarcinoma
KIRP: Kidney renal papillary cell carcinoma
LUSC: Lung squamous cell carcinoma
READ: Rectum adenocarcinoma
HCC: Hepatocellular carcinoma
UCEC: Uterine corpus endometrial carcinoma
MESO: Mesothelioma
LIHC: Liver hepatocellular carcinoma
KIRP: Kidney renal papillary cell carcinoma
CHOL: Cholangiocarcinoma
ACC: Adrenocortical carcinoma

## References

[1] Sung H (2021). Global cancer statistics 2020: GLOBOCAN estimates of incidence and mortality worldwide for 36 cancers in 185 countries. CA Cancer J Clin.

[2] Shin WS (2023). Updated epidemiology of gastric cancer in Asia: decreased incidence but still a big challenge. Cancers.

[3] Henson DE (2004). Differential trends in the intestinal and diffuse types of gastric carcinoma in the United States, 1973–2000: increase in the signet ring cell type. Arch Pathol Lab Med.

[4] Wu H (2009). Stomach carcinoma incidence patterns in the United States by histologic type and anatomic site. Cancer Epidemiol Biomarkers Prev.

[5] Bencivenga M, Dal Cero M, de Manzoni G, Roviello F (2022). Focus on poorly cohesive gastric cancer. Gastric cancer: the 25-year R-evolution.

[6] Nakamura K (2022). Clinicopathological characteristics and prognosis of poorly cohesive cell subtype of gastric cancer. Int J Clin Oncol.

[7] Drubay V (2022). Poorly cohesive cells gastric carcinoma including signet-ring cell cancer: updated review of definition, classification and therapeutic management. World J Gastrointest Oncol.

[8] Bass AJ (2014). Comprehensive molecular characterization of gastric adenocarcinoma. Nature.

[9] Li B (2023). A molecular classification of gastric cancer associated with distinct clinical outcomes and validated by an XGBoost-based prediction model. Mol Ther Nucleic Acids.

[10] Oh SC (2018). Clinical and genomic landscape of gastric cancer with a mesenchymal phenotype. Nat Commun.

[11] Jang E (2023). Clinical molecular subtyping reveals intrinsic mesenchymal reprogramming in gastric cancer cells. Exp Mol Med.

[12] Zhou X (2023). Relationships of tumor differentiation and immune infiltration in gastric cancers revealed by single-cell RNA-seq analyses. Cell Mol Life Sci.

[13] Jo HH (2023). The clinicopathological features of mixed carcinoma in 7,215 patients with gastric cancer in a tertiary hospital in South Korea. Gut Liver.

[14] Garcia-Pelaez J (2021). Histological and mutational profile of diffuse gastric cancer: current knowledge and future challenges. Mol Oncol.

[15] Quail DF (2022). Neutrophil phenotypes and functions in cancer: a consensus statement. J Exp Med.

[16] Bui TM, Yalom LK, Sumagin R (2021). Tumor-associated neutrophils: orchestrating cancer pathobiology and therapeutic resistance. Expert Opin Ther Targets.

[17] Singhal S (2016). Origin and role of a subset of tumor-associated neutrophils with antigen-presenting cell features in early-stage human lung cancer. Cancer Cell.

[18] Gibellini L (2023). Circulating and Tumor-Associated Neutrophils in the Era of Immune Checkpoint Inhibitors: Dynamics, Phenotypes, Metabolism, and Functions. Cancers (Basel).

[19] Sionov RV, Fridlender ZG, Granot Z (2015). The multifaceted roles neutrophils play in the tumor microenvironment. Cancer Microenviron.

[20] Liu YJ (2023). USP51/ZEB1/ACTA2 axis promotes mesenchymal phenotype in gastric cancer and is associated with low cohesion characteristics. Pharmacol Res.

[21] Ramos-Vara JA (2017). Principles and methods of immunohistochemistry. Methods Mol Biol.

[22] Liu YJ (2021). FSTL3 is a prognostic biomarker in gastric cancer and is correlated with M2 macrophage infiltration. Onco Targets Ther.

[23] Menon RT (2020). Adrenomedullin is necessary to resolve hyperoxia-induced experimental bronchopulmonary dysplasia and pulmonary hypertension in mice. Am J Pathol.

[24] Kim B (2017). Western blot techniques. Methods Mol Biol.

[25] Tabatabaei MS, Ahmed M (2022). Enzyme-linked immunosorbent assay (ELISA). Methods Mol Biol.

[26] Lin X (2022). Identification of novel immunomodulators in lung squamous cell carcinoma based on transcriptomic data. Math Biosci Eng.

[27] Miao S (2019). Cancer cell-derived immunoglobulin G activates platelets by binding to platelet FcγRIIa. Cell Death Dis.

[28] Li Y (2021). IGHG1 induces EMT in gastric cancer cells by regulating TGF-β/SMAD3 signaling pathway. J Cancer.

[29] Wang K (2017). Breast cancer cells alter the dynamics of stromal fibronectin-collagen interactions. Matrix Biol.

[30] Li J (2022). High FN1 expression correlates with gastric cancer progression. Pathol Res Pract.

[31] Antony J, Chin CV, Horsfield JA (2021). Cohesin mutations in cancer: emerging therapeutic targets. Int J Mol Sci.

[32] Liew PX, Kubes P (2019). The neutrophil's role during health and disease. Physiol Rev.

[33] Amulic B (2012). Neutrophil function: from mechanisms to disease. Annu Rev Immunol.

[34] Hedrick CC, Malanchi I (2022). Neutrophils in cancer: heterogeneous and multifaceted. Nat Rev Immunol.

[35] Coffelt SB, Wellenstein MD, de Visser KE (2016). Neutrophils in cancer: neutral no more. Nat Rev Cancer.

[36] Shaul ME, Fridlender ZG (2019). Tumour-associated neutrophils in patients with cancer. Nat Rev Clin Oncol.

[37] Giese MA, Hind LE, Huttenlocher A (2019). Neutrophil plasticity in the tumor microenvironment. Blood.

[38] Que H (2022). Tumor-associated neutrophils and neutrophil-targeted cancer therapies. Biochim Biophys Acta Rev Cancer.

[39] Xue R (2022). Liver tumour immune microenvironment subtypes and neutrophil heterogeneity. Nature.

[40] Chen J (2012). Interferon-γ-induced PD-L1 surface expression on human oral squamous carcinoma via PKD2 signal pathway. Immunobiology.

[41] Gao Y (2023). Fusobacterium nucleatum stimulates cell proliferation and promotes PD-L1 expression via IFIT1-related signal in colorectal cancer. Neoplasia.

[42] Iyer P (2020). Diffuse gastric cancer: histologic, molecular, and genetic basis of disease. Transl Gastroenterol Hepatol.

[43] Qi J (2022). Single-cell and spatial analysis reveal interaction of FAP(+) fibroblasts and SPP1(+) macrophages in colorectal cancer. Nat Commun.

[44] Liu Y (2023). Identification of a tumour immune barrier in the HCC microenvironment that determines the efficacy of immunotherapy. J Hepatol.

[45] Butterfield TA, Best TM, Merrick MA (2006). The dual roles of neutrophils and macrophages in inflammation: a critical balance between tissue damage and repair. J Athl Train.

[46] Long W (2021). Brief review on the roles of neutrophils in cancer development. J Leukoc Biol.

[47] Zhou SL (2016). Tumor-associated neutrophils recruit macrophages and t-regulatory cells to promote progression of hepatocellular carcinoma and resistance to sorafenib. Gastroenterology.

[48] Mahmud Z (2022). Mechanistic insights into the interplays between neutrophils and other immune cells in cancer development and progression. Cancer Metastasis Rev.

[49] Matsubara E (2023). The significance of SPP1 in lung cancers and its impact as a marker for protumor tumor-associated macrophages. Cancers.

[50] De Filippo K (2013). Mast cell and macrophage chemokines CXCL1/CXCL2 control the early stage of neutrophil recruitment during tissue inflammation. Blood.

[51] Cambier S, Gouwy M, Proost P (2023). The chemokines CXCL8 and CXCL12: molecular and functional properties, role in disease and efforts towards pharmacological intervention. Cell Mol Immunol.

[52] Szabo PM (2023). Cancer-associated fibroblasts are the main contributors to epithelial-to-mesenchymal signatures in the tumor microenvironment. Sci Rep.

[53] Fensterl V, Sen GC (2015). Interferon-induced Ifit proteins: their role in viral pathogenesis. J Virol.

[54] von Locquenghien M, Rozalén C, Celià-Terrassa T (2021). Interferons in cancer immunoediting: sculpting metastasis and immunotherapy response. J Clin Invest.

[55] Borden EC (2019). Interferons α and β in cancer: therapeutic opportunities from new insights. Nat Rev Drug Discov.

[56] Garcia-Diaz A (2017). Interferon receptor signaling pathways regulating PD-L1 and PD-L2 expression. Cell Rep.

[57] Spranger S (2013). Up-regulation of PD-L1, IDO, and T(regs) in the melanoma tumor microenvironment is driven by CD8(+) T cells. Sci Transl Med.

[58] de Kleijn S (2013). IFN-γ-stimulated neutrophils suppress lymphocyte proliferation through expression of PD-L1. PLoS ONE.

[59] Ayers M (2017). IFN-γ-related mRNA profile predicts clinical response to PD-1 blockade. J Clin Invest.

[60] Kock Am Brink M (2023). Intratumoral heterogeneity affects tumor regression and Ki67 proliferation index in perioperatively treated gastric carcinoma. Br J Cancer.

[61] Bonelli P (2019). Precision medicine in gastric cancer. World J Gastrointest Oncol.

[62] Koseki Y (2023). Molecular profile of poorly cohesive gastric carcinoma with special reference to survival. Gastric Cancer.

[63] Serra O (2019). Comparison and applicability of molecular classifications for gastric cancer. Cancer Treat Rev.

[64] Baek JH (2023). Clinical implications and chemo-sensitivity of adjuvant chemotherapy in patients with poorly cohesive cells-gastric cancer. Cancer Chemother Pharmacol.

[65] Gullo I (2018). Heterogeneity in gastric cancer: from pure morphology to molecular classifications. Pathobiology.

[66] Tie Y (2022). Immunosuppressive cells in cancer: mechanisms and potential therapeutic targets. J Hematol Oncol.

[67] Zhang H (2021). Regulatory mechanisms of immune checkpoints PD-L1 and CTLA-4 in cancer. J Exp Clin Cancer Res.

[68] Chandran SS, Klebanoff CA (2019). T cell receptor-based cancer immunotherapy: Emerging efficacy and pathways of resistance. Immunol Rev.

[69] Alsuliman A (2015). Bidirectional crosstalk between PD-L1 expression and epithelial to mesenchymal transition: significance in claudin-low breast cancer cells. Mol Cancer.

[70] Geh D (2022). Neutrophils as potential therapeutic targets in hepatocellular carcinoma. Nat Rev Gastroenterol Hepatol.

[71] Wang MM (2023). Resistance to immune checkpoint therapies by tumour-induced T-cell desertification and exclusion: key mechanisms, prognostication and new therapeutic opportunities. Br J Cancer.

[72] Denk D, Greten FR (2022). Inflammation: the incubator of the tumor microenvironment. Trends Cancer.

[73] Zhang Z (2022). Heterogeneous cancer-associated fibroblasts: a new perspective for understanding immunosuppression in pancreatic cancer. Immunology.

[74] Mao X (2021). Crosstalk between cancer-associated fibroblasts and immune cells in the tumor microenvironment: new findings and future perspectives. Mol Cancer.

[75] Zhang Y (2019). The roles of ZEB1 in tumorigenic progression and epigenetic modifications. Biomed Pharmacother.

[76] Krebs AM (2017). The EMT-activator Zeb1 is a key factor for cell plasticity and promotes metastasis in pancreatic cancer. Nat Cell Biol.

[77] Mandai M (2016). Dual faces of IFNγ in cancer progression: a role of PD-L1 induction in the determination of pro- and antitumor immunity. Clin Cancer Res.

[78] Salmaninejad A (2019). PD-1/PD-L1 pathway: basic biology and role in cancer immunotherapy. J Cell Physiol.

[79] Doroshow DB (2021). PD-L1 as a biomarker of response to immune-checkpoint inhibitors. Nat Rev Clin Oncol.

[80] Yamaguchi H (2022). Mechanisms regulating PD-L1 expression in cancers and associated opportunities for novel small-molecule therapeutics. Nat Rev Clin Oncol.

[81] Kornepati AVR, Vadlamudi RK, Curiel TJ (2022). Programmed death ligand 1 signals in cancer cells. Nat Rev Cancer.

[82] Bellucci R (2015). Interferon-γ-induced activation of JAK1 and JAK2 suppresses tumor cell susceptibility to NK cells through upregulation of PD-L1 expression. Oncoimmunology.

[83] Imai D (2019). IFN-γ promotes epithelial-mesenchymal transition and the expression of PD-L1 in pancreatic cancer. J Surg Res.

