# Supplementary material for: IFIT1 + neutrophil is a causative factor of immunosuppressive features of poorly cohesive carcinoma (PCC)
Source: J Transl Med. 2024 Jun 19;22:580. doi: 10.1186/s12967-024-05389-z (PMC11188200; doi:10.1186/s12967-024-05389-z)
Supplement: Supplementary file 1 — Supplementary Material 1. [file 12967_2024_5389_MOESM1_ESM.docx]

# Supplementary Material

## Supplementary Tables

### Table S1

| Antibodies and Reagents | Manufacturer, Country, Cat number, Lot number | Concentration |
| --- | --- | --- |
| Roswell Park Memorial Institute 1640 (RPMI-1640) | Gibco, USA, C11875500CP, 8123501 | - |
| Dulbecco’s modified Eagle’s medium (DMEM) | Gibco, USA, C11995500CP, 8123472 | - |
| Phosphate buffered saline (PBS) | Bio-Channel, China, BC-BPBS-01, BC20231110 | - |
| Fetal bovine serum (FBS) | Gibco, USA, 10099-141, 1907403 | - |
| Trypsin | Gibco, USA, 25300-054, R001100 | - |
| Matrigel | Corning, USA, 356234, 234507 | - |
| Puromycin | Beyotime Biotechnology, China, ST551, 041321210517 | - |
| Bicinchoninic acid (BCA)  Assay Kit | Vazyme, China, E112-02,7E710H3 | 1.5 μg/mL |
| IFN Gamma Polyclonal antibody | Proteintech, USA, 15365-1-AP, 20001029 | IF 1:200 |
| Human Recombinant IFN-γ | Procell Life Science&Technology, China, PCK062, WH0223C687 | 10ng/ml, 100ng/ml |
| Anti-β-actin Mouse polyclonal Antibody | Proteintech, USA, 6609-1-Ig, 10021293 | WB 1:5000 |
| Anti-GAPDH Mouse polyclonal Antibody | Proteintech, USA, 6004-1-Ig, 10020246 | WB 1:3000 |
| Anti-ZEB1 Rabbit polyclonal antibody | Cell Signaling Technology, USA, 3396, 6 | WB 1:1000  IF 1:200 |
| Anti-α-Smooth Actin Rabbit polyclonal antibody | Cell Signaling Technology, USA, 19245, 5 | IF 1:200 |
| Anti-F-actin Rabbit polyclonal Antibody | Cell Signaling Technology, USA, 6487, 4 | WB 1:1000  IF 1:100 |
| Anti-FAP Rabbit polyclonal Antibody | Cell Signaling Technology, USA, 66562, 5 | IF 1:100 |
| Osteopontin Polyclonal Antibody | Invitrogen, USA, PA5-141129, YK4114254D | IF 1:100 |
| CEACAM8 (CD66b) Polyclonal Antibody | Affinity Biosciences, USA, DF10151, 85u0774 | IF 1:200 |
| IFIT1 Rabbit Polyclonal Antibody | Proteintech, USA,23247-1-AP, 00093570 | WB 1:1000  IF 1:200 |
| PD-1 Rabbit Polyclonal Antibody | Proteintech, USA,18106-1-AP, 00091945 | IF 1:200 |
| Endoglin/ENG Rabbit Polyclonal Antibody | Proteintech, USA,67075-1-Ig, 10010278 | IF 1:200 |
| PD-L1 Rabbit Polyclonal Antibody | Abcam, England, ab205921,00052042 | IHC: 1:500  IF 1:200 |
| Anti-CK Rabbit polyclonal Antibody | Cell Signaling Technology, USA, 12509, 3 | WB 1:1000  IF 1:200 |
| Anti-CD8 Mouse polyclonal Antibody | Cell Signaling Technology, USA, 55397, 3 | WB 1:1000  IF 1:400 |
| CoraLite488-conjugated Goat Anti-Rabbit IgG(H+L) | Proteintech, USA, SA00013-2, 205001014 | IF 1:300 |
| CoraLite594-conjugated Goat Anti-Mouse IgG(H+L) | Proteintech, USA, SA00013-3, 20000154 | IF 1:300 |
| CoraLite647-conjugated Mouse Anti-Heavy Chain of Rabbit IgG | Proteintech, USA, SA00014-6, 20000129 | IF 1:300 |
| Highly Cross-Adsorbed Goat (Polyclonal) Anti-Mouse IgG(H+L) Antibody | LI-COR, USA, 925-68070, D20427-21 | WB 1:5000 |
| Highly Cross-Adsorbed Goat (Polyclonal) Anti-Rabbit IgG(H+L) Antibody | LI-COR, USA, 925-68071, D20601-11 | WB 1:5000 |
| Human Nicotinamide Phosphoribosyltransferase (NAMPT) ELISA Kit\| | FineTest, China, EH0651, E024 | - |
| Human VEGF-A ELISA Kit | Shanghai Enzyme Linked Biology, mlsw_E1254, 06/2023 | - |
| Human CXCL8 ELISA Kit | BYabscience, China, BY-EH113147, E20220720-BY-EH113147B | - |
| Human CXCR2 ELISA Kit | BYabscience, China, BY-EH110351, E20220720-BY-EH110351B | - |
| 4',6-diamidino-2-phenylindole  (DAPI) | Beyotime Biotechnology, China, C1002, 091620210520 | - |
| EasySep^TM^ Human Neutrophil Enrichment Kit | STEMCELL Technologies, Canada, 17957, 1000140311 | - |
| Hematoxylin-Eosin staining Kit | Solarbio life sciences, China, G1120, 20220325 | - |
| Giemsa staining Kit (20×) | Beyotime Biotechnology, China, C0131-100ml, 021352230826 |  |
| PE anti-human CD11b | BioLegend, USA, clone: LM2, 393112, B353524 | 2ul/1×10^6^ Cell |
| Alexa Fluor® 647 anti-human CD66b | BioLegend, USA, clone:6/40C, 392912, B374318 | 2ul/1×10^6^ Cell |
| Alexa Fluor® 488 anti-human CD3 | BioLegend, USA, clone: OKT3, 317310, B369206 | 2ul/1×10^6^ Cell |
| PE anti-human CD8 | BioLegend, USA, clone:SK1, 344706, B385781 | 5ul/1×10^6^ Cell |
| PE anti-human PD1 | BioLegend, USA, clone:EH12.2H7,329940,  B382493 | 2.5ul/1×10^6^ Cell |
| A MojoSort mouse neutrophil isolation kit | BioLegend, USA,480058, B397014 | - |
| Alexa Fluor® 647 anti-human CD69 | BioLegend, USA, clone: FN50, 310918, B369207 | 5ul/1×10^6^ Cell |
| FITC anti-mouse CD3 | BioLegend, USA, clone:17A2,100204,  B388315 | 2ul/1××10^6^ Cell |
| PE anti-mouse CD8b | BioLegend, USA, clone: YTS156.7.7,126608,  B393495 | 2.5ul/1××10^6^ Cell |
| Alexa Fluor® 647 anti-mouse CD69 | BioLegend, USA, clone:H1.2F3,104518,  B353524 | 5ul/1××10^6^ Cell |
| PE anti-mouse PD1 | BioLegend, USA, clone:29F.1A12,135206,  B374318 | 5ul/1××10^6^ Cell |
| FTTC anti-mouse CD45 | BioLegend, USA, clone:30-F11,103108,  B330230 | 5ul/1××10^6^ Cell |
| PE anti-mouse Ly-6G | BioLegend, USA, clone:1A8,127608,  B388208 | 5ul/×10^6^ Cell |
| ExpressPlus™ PAGE Gel, 10x8, 10%, 15 wells | GenScript, USA, M00666, C35652311 | - |
| Tricolor Prestained Protein Marker | EpiZyme, China, WJ103, 027352000 | - |
| Crystal violet | Beyotime Biotechnology, China, C0121-100ml,121322230524 | - |
| 4% Paraformaldehyde | Biosharp, China, BL539A, 22329929 | - |
| Protease and phosphatase inhibitor cocktail for genneraluse,50X | Beyotime Biotechnology, China, P1045, 051823230618 | - |
| Loading buffer | Epizyme, China, LT103s, 01752056 | - |
| RBC Lysis Buffer (10×) | Biosharp, China, CS003, 220903 | - |
| bovine serum albumin (BSA) | Vazyme,USA, B2270DBA, 027E2270DA | - |
| Collagenase I | Biosharp, China, BS164-100mg, B0013K030100 | - |
| Deoxyribonuclease I | Beyotime Biotechnology, China, D7073, 112522230619 | - |
| Radioimmunoprecipitation assay buffer (RIPA buffer) | Beyotime Biotechnology, China, P0013B, 052523230703 | - |
| Cell Counting Kit-8 (CCK-8) | APExBIO, America, K1018,  K101828 | - |
| Calcein-AM/PI | Solarbio, China, CA1630,  2310008 | 2uM (AM),  4uM (PI) |
| Human CXCL2 ELISA KIT | Solarbio, China, SEKH-0066,  2312005 | - |

### Table S2

| Module-3 members | *CD69*, *PLEK*, *HCAR*3, *SAT1*, *IL1RN*, *GK*, *TAGAP*, *FCGR2A*, *N4BP2*, *APOL6*, *HCAR2*, *IFITM3*, *PPIF*, *SOCS3*, *HERC5*, *MX1*, *IFIT2*, *RSAD2*, *IFIT3*, *RNF213*, *LCP2*, *IFIT1*, *RHOH*, *GBP1*, *ISG15* |
| --- | --- |

### Table S3

**Clinicopathological information of** **patients subjected to single-cell sequencing**

|  | **Patient number** | **Gender** | **Age (years)** | **Stage** | **Lauren classification** | **single-cell RNA-seq sample** **number** |
| --- | --- | --- | --- | --- | --- | --- |
| NPCC | #1 | female | 66 | Ⅳ  (T2aN2M1) | intestinal type | N1-T1 |
|  | #2 | male | 61 | Ⅳ  (T4aN1M) | intestinal type | N2-T2 |
|  | #3 | female | 58 | Ⅳ  (T3aN2M1) | mixed type | T4 |
|  | #4 | male | 69 | Ⅳ  (T4aN3bMx) | diffuse type | T5 |
|  | #5 | male | 57 | ⅢA (T2aN3aM0) | mixed type | T8 |
|  | #6 | male | 68 | ⅢC (T4aN3bM0) | mixed type | T9 |
| PCC | #1 | female | 56 | Ⅳ  (T2aN3aMx) | mixed type | N3-T3 |
|  | #2 | male | 52 | Ⅳ  (T4bN3aM1) | diffuse type | T6 |
|  | #3 | male | 69 | IIIA  (T2aN3aM0) | mixed type | T9 |

**IFIT1 RNAi and overexpression preparation**

**Gene information**

Gene symbol：*IFIT1* (Interferon induced protein with tetratricopeptide repeats 1)

Organism: human

### Table S4

**Easy-siRNA** **design**

| **NO.** | **Accession** | **Target Seq (5’→ 3’)** | **Titer (TU/mL)** |
| --- | --- | --- | --- |
| IFIT1-RNAi (232351-1) | NM_001270927.2 | Forward: TCTCAGAGGAGCCTGGCTAA  Reverse: TCAGGCATTTCATTCGTCATC | 6E+8 |
| IFIT1-RNAi (232352-1) | NM_001270927.2 | Forward:  GTGCTTGAAGTGGACCCTGA  Reverse:  CCTGCCTTAGGGGAAGCAAA | 8E+8 |
| IFIT1-RNAi (232353-1) | NM_001270927.2 | Forward:  CTTCGGAGAAAGGCATTAGAT  Reverse:  CGGAACAGCAGAGACACAGA | 7E+8 |

IFIT1-RNAi (232352-1) was chosen because it had the highest silencing efficiency

lentivirus vector name: GV493 (hU6-MCS-Ubiquitin-IRES-puromycin)

Negative Control insert sequence: TTCTCCGAACGTGTCACGT

### Table S5

**Obtain the target gene fragments**

| **ID** | **Seq (5’→ 3’)** |
| --- | --- |
| IFIT1 (89179-1)-p1 | Forward: TTTTCTCGAGGCCGCCACCATGAGTACAAATGGTGATGATC |
| IFIT1 (89179-1)-p2 | Reverse: AAAGCTAGCCTAAGGACCTTGTCTCACAGAGTTC |

lentivirus overexpression vector name：GV492 (Ubi-MCS-3FLAG-CBh-IRES-puromycin), Titer（TU/ml）:1.5E+9.

**Obtain the target gene fragments**

| **ID** | **Seq (5’→ 3’)** |
| --- | --- |
| SPP1  (92403-1)-p1 | Forward: CCGGAATTCATGCAGGCGCGCTACTCGG |
| SPP1 (92403-1)-p2 | Reverse:  CGGGATCCTCAGAATTTGCTACAGTCATAGACGAAAGCC |

lentivirus overexpression vector name：GV492 (Ubi-MCS-3FLAG-CBh-IRES-puromycin), Titer (TU/ml): 2.0E+9.

**ZEB1 overexpression preparation**

**Gene information**

Gene symbol：*ZEB1* (zinc finger E-box binding homeobox 1)

Organism: human

### Table S6

**Obtain the target gene fragments**

| **ID** | **Seq (5’→ 3’)** |
| --- | --- |
| ZEB1 (74048-1)-p1 | Forward: CGGGATCCCGATGGCGGATGGCCCCAGGT |
| ZEB1 (74049-1)-p2 | Reverse: CCGCTCGAGCGGTTAGGCTTCATTTGTCT |

lentivirus overexpression vector name：GV657(CMV enhancer-MCS-3flag-polyA-EF1A-sv40-puromycin) Titer（TU/ml）:1E+9.

**Table S7**

Organism: mouse

**Easy-siRNA** **design**

| **NO.** | **Accession** | **Target Seq (5’→ 3’)** | **Titer (TU/mL)** |
| --- | --- | --- | --- |
| IFIT1-RNAi (242130-1) | NM_008331.3 | Forward: CCUACCUGCAUUGCCAAAUTT  Reverse:  AUUUGGCAAUGCAGGUAGGTT | 9E+8 |
| IFIT1-RNAi (242131-1) | NM_008331.3 | Forward:  GCCAGACAAAGCGAUUGAATT  Reverse:  UUCAAUCGCUUUGUCUGGCTT | 8.5E+8 |
| IFIT1-RNAi (242132-1) | NM_008331.3 | Forward:  GCCAGACAAAGCGAUUGAATT  Reverse:  UUCAAUCGCUUUGUCUGGCTT | 8E+8 |

IFIT1-RNAi (232352-1) was chosen because it had the highest silencing efficiency

lentivirus vector name: GV493 (hU6-MCS-CBh-IRES-puromycin)

Negative Control insert sequence: UUCUCCGAACGUGUCACGUTT

**Table S8**

**Obtain the target gene fragments**

| **ID** | **Seq (5’→ 3’)** |
| --- | --- |
| IFIT1 (90674-1)-p1 | Forward: TTTTCTCGAGGCCGCCACCATGGCAGTGACAACTCGTTTGAC |
| IFIT1 (90674-1)-p2 | Reverse: CCCGCTAGCCTACCCGCTAGCCTATTTTTTTCCTTGTGCACAGTTGATAATTTCCTCCCTTAGATTC |

lentivirus overexpression vector name：GV492 (Ubi-MCS-3FLAG-CBh-IRES-puromycin), Titer（TU/ml）:8E+8.

**Table S9**

Organism: human

**Easy-siRNA** **design**

| **NO.** | **Accession** | **Target Seq (5’→ 3’)** | **Titer (TU/mL)** |
| --- | --- | --- | --- |
| SPP1-RNAi (243671-1) | NM_001040060 | Forward: GCTCAGGCCGAGTGTACTA  Reverse:  GCTCAGGCCGAGTGTACTA | 2.5E+9 |
| SPP1-RNAi (243672-1) | NM_001040060 | Forward:  TAGTACACTCGGCCTGAGC  Reverse:  TAGTACACTCGGCCTGAGC | 9E+8 |
| SPP1-RNAi (243673-1) | NM_001040060 | Forward:  GATCCGAACTACACCTTC  Reverse:  TGAAGGTGTAGTTCGGATC | 9.5E+8 |

SPP1-RNAi (243673-1) was chosen because it had the highest silencing efficiency

lentivirus vector name: GV493 (hU6-MCS-CBh-IRES-puromycin)

Negative Control insert sequence: CAACAAGATGAAGAGCACCAA

**Table S10**

**Clinicopathological information of PCC and NPCC patients**

| Characteristics |  |
| --- | --- |
| Age (years) |  |
| Mean (SD) | 61.56 ± 12.16 |
| Median (min, max) | 52 (33, 79) |
| Gender, n (%) |  |
| Female | 31 (48.44%) |
| Male | 33 (51.56%) |
| pathological type, n (%) |  |
| PCC | 30 (33.33%) |
| NPCC | 34 (6.67%) |
| TNM stage, n (%) |  |
| I | 17 (26.56%) |
| II | 26 (40.63%) |
| III&IV | 21 (32.81%) |

(There were 3 PCC with paired adjacent normal samples, and 3 NPCC with paired adjacent normal samples. Adjacent normal tissues were obtained from regions outside the tumor margin >10cm in patients.)

# Supplementary Methods

## Inclusion/exclusion criteria for participants

To evaluate the association between IFIT1 expression levels and immunotherapy efficacy, a retrospective cohort observational study was established. This study involved a cohort of 40 patients who underwent treatment for GC at the Jiangsu Province Hospital of Chinese Medicine over the period from May 1, 2021 to May 1, 2023. Specimens obtained from patients were collected and stored following the approved protocols. Anonymization and de-identification of all patient records were conducted before analysis. Inclusion criteria: (1) All patients diagnosed with GC for the first time had clinical examination, gastroscopy, magnetic resonance imaging (MRI), positron emission tomography (PET)-Computed tomography (CT)/CT, and hematological diagnostics. All patient’s pathology samples were saved for independent validation by two different specialists. (2) Complete clinical data were available for every patient. We evaluated the efficacy of those patients who were receiving traditional chemotherapy (SOX: S-1 plus Oxaliplatin or XELOX: Capecitabine plus Oxaliplatin) in combination with Nivolumab (360mg intravenously every 3 weeks). A PET-CT or CT scan was conducted every 6 weeks or as required by the patient's clinical condition to assess their radiologic status. was the basis for tumor response evaluation. The study excluded the following patients: (1) those with a pathological diagnosis other than GC, such as a gastric stromal tumor; (2) patients who died during treatment; (3) patients with incomplete data or who were lost to follow-up; (4) patients with an infection, immune system disorder, or blood system condition; (5) patients who underwent local radiotherapy or radiofrequency ablation; (6) patients who were HIV positive; and (7) patients who were unable to tolerate adverse reactions.

## Public datasets

For 375 Stomach adenocarcinoma (STAD) patients, RNA-seq transcriptome information was downloaded from The Cancer Genome Atlas (TCGA) portal along with clinical data(21), while a portion of the TCGA data was processed using the Gene Set Cancer Analysis (GSCA) software. In addition, pan-cancer data were obtained by the same approach. For GC, additional independent validation cohorts (GSE66229, GSE15459, GSE13911, GSE79973, and GSE54129) were obtained from the Gene Expression Omnibus (GEO) database(10, 22-24). Afterward, the 5 datasets were integrated as a merged GEO (mGEO) dataset, and the “Combat” function of the “SVA” package was conducted to eliminate batch effect. To validate the Differentially expressed genes (DEGs) between PCC and NPCC malignant cells obtained from single cell RNA (scRNA) data, a dataset GSE211512 containing sequencing data of xenograft tumors from MKN45 and MKN74 cell lines was used(25). In addition, two immunotherapy datasets (PRJEB25780 and GSE120575) were utilized to measure the significance of the study objectives for immunotherapy(26, 27), where GSE120575 was processed through Tumor Immunotherapy Gene Expression Resource (TIGER) online tool. All the gene expression data that do not depend on online tools for processing are output in the form of a normalized data matrix with the help of R software (version: 4.1.2).

## high dimensional weighted gene co-expression network analysis (hdWGCNA)

In high-dimensional data sets, such as single-cell RNA-seq, hdWGCNA can be used to perform WGCNA(28). In addition to constructing co-expression networks in a cell-type-specific manner, hdWGCNA identifies robust modules of related genes and provides their biological context. The “SetDatExpr” function was conducted to specify neutrophils for constructing the expression matrix. Then, the “TestSoftPowers” function was performed for parameter scans to determine the optimal soft power threshold β (β = 4) for constructing the WGCNA network. Based on the given threshold value, the adjacency matrix and topological overlap matrix (TOM) were generated. We performed the dynamicTreeCut algorithm to identify co-expression modules from the TOM and assigned each module a unique color. In addition, hub genes within each module were assessed for connectivity using eigengene-based connectivity (kME) values obtained by the “ModuleConnectivity” function. Genes with higher kME values are considered more important. To summarize the gene signatures in each expression module, the “GetMEs” function was used to calculate the module eigengenes (MEs), and the correlation among MEs was measured using the Pearson method. Finally, based on kME ordering, a PCC subgroup gene signature was screened for subsequent bioinformatics analysis.

## Construction of neutrophils (module 3 obtained by hdWGCNA) related phenotypes

We extracted a PCC-associated neutrophil module from the hdWGCNA results (Module 3, Table S2). In order to stratify patients based on module-3 phenotypes, we initially performed unsupervised clustering analysis using transcriptional profiles of 25 module-3 molecules. Consensus clustering algorithm determined the optimal clustering number for the STAD-Cohort based on multiple criteria, and its repeatability was verified in mGEO. The consensus clustering procedure was carried out using the R package “ConsensusClusterPlus”(21).

## Identification of DEGs

The R package “Limma” was used to identify DEGs between two phenotypes(21). The significance criterion was set at adjust P < 0.05, and |log2 fold change (FC)| > 1.

## Enrichment analysis

To analyze the enrichment of Gene Ontology (GO) and Kyoto Encyclopedia of Genomes (KEGG), and HALLMARK, the “ClusterProfiler” toolkit was used(22).

## Friends analysis

The Friends analysis approach assesses the functional correlation between different genes in a pathway, suggesting that a gene is more likely to be expressed if it interacts with other genes in the same pathway, and it is widely used to identify critical genes. In this study, the “GOSemSim” package was used to identify critical genes of interest to us.

## Proteomic analysis

For proteomics data, all analyses were performed on the LinkedOmicsKB platform(23).

## Spatial transcriptomics (ST) analysis

The STOmicsDB online tool (https://db.cngb.org/) was conducted to analyze the spatial expression of genes at the level of pan-cancer. Moreover, the ST dataset GSE186290(24), was downloaded to analyze the spatial expression levels and overlapping of *IFIT1* and other cell markers in GC.

## Preparation of single-cell suspensions from surgical specimen

Single-cell sequencing was performed to analyze 12 samples from 9 GC patients who had not received adjuvant treatment, including chemotherapy or radiotherapy, prior to the surgery in order to eliminate potential treatment-induced changes to gene expression profiles. Specifically, information about specimen site, cancer diagnosis, cancer subtype, and pathological Tumor-node-metastasis (TNM) stage information was shown in Table S3. 3 Normal mucosa (NM) samples refer to samples were cut at least 10 cm away from tumor margin. 9 GC tissues were obtained from 3 PCC patients and 6 NPCC patients. Every fresh excised specimen from the surgical intervention underwent subsequent preparation: Prior to digestion, the biopsy material was finely dissected with an Iris scissors and then subjected to a 30-minute incubation at 37 °C and 800 rpm with a digestion solution consisting of Phosphate-buffered saline (PBS). This was followed by a one-hour incubation at 37 °C with trypsin, collagenase I, and DNase. Subsequent to the addition of 4 mL of Dulbecco's modified Eagles medium (DMEM), the suspension was filtered via a 40-μm cell mesh. After centrifuging at 250 g for 5 minutes, the supernatant was discarded, and the cells were washed twice in PBS. Afterwards, the tube was subjected to centrifugation at 250 g for 10 minutes after the resuspended of 10 mL of precooled PBS. The precipitated cells were then resuspended in 5 mL of PBS without calcium. Ultimately, the single-cell suspensions were quantified using an inverted microscope and a hemocytometer. The count of live cells was detected using trypan blue. The proportion of living cells was great than 90%.

Single-cell sequencing was performed at the single-cell sequencing core in BMK Biotechnology Company. Single cells were loaded into chromium microfluidic chips with v3 chemistry and barcoded with a 10× chromium controller (10× Genomics). RNA from the barcoded cells was reverse-transcribed, and sequencing libraries were constructed with a Chromium Single Cell v3 reagent kit (10× Genomics). Sequencing was performed on NovaSeq 6000 (Illumina).

## Single-cell transcriptome sequencing analysis

A single-cell RNA matrix was constructed after identifying cells with CellRanger, low-quality cells (the number of mitochondrial transcripts > 20%, and expressed genes < 200 or > 9,000) were excluded according to standard scRNA-seq filtering, and finally the data were obtained for cluster analysis. First, highly variable genes (top 2000 genes with the highest variance) were selected for data normalization, principal component analysis (PCA) was performed using the top 30 principal components, and the “Harmony” function was used to correct for batch effects among the samples. A total of nine large cell lineages (T cells, B cells, neutrophils, epithelial cells, endothelial cells, fibroblasts, macrophages, mast cells, and NK cells) were identified by UMAP cluster analysis. Gene expression matrices for each cell population were then extracted for further subpopulation analysis. Wilcoxon rank sum test was used to identify genes that were differentially expressed between subpopulations.

For cell developmental trajectory analysis, “Monocle3” was used for inferring pseudotime series(25). After constructing the cell trajectories, we used the “graph_test” function to obtain the DEGs along the pseudotime and used the q-value < 1e-5 as the screening threshold. For cell-cell communication analysis, ligand-receptor information from the “CellChat” library was used to analyze the potential information exchange between each cell subpopulation, and the standard analysis procedure was described previously(26).

## Immune infiltration estimation based on bulk RNA data

6 immune estimation algorithms are included in the “IOBR” package, a newly developed software package(27). The relationship between TME composition and our phenotypes of interest was explored by applying the “IOBR” package. Next, the “BisqueRNA” package was used to construct reference bases for expression profiles across specialized cell types from scRNA-seq data and estimate cell proportions from bulk expression (TCGA-STAD and mGEO)(28). Furthermore, estimation of stroma and immune cells in malignant tumor tissues was performed using bulk data (ESTIMATE) algorithm to quantify stroma score, immune score, and estimated score for each GC sample(29).

## Cell culture

Human GC cell lines MKN74 (from moderately differentiated tubular adenocarcinoma) and MKN45 (from poorly differentiated adenocarcinoma, signet ring cell carcinoma) were provided by a 113-cell repository of the China Academy of Sciences (Shanghai, China). Procell Life Science and Technology Co. Ltd. supplied Mouse Forestomach Carcinoma (MFC) and human cancer-associated fibroblasts (CAFs) derived from GC tissue. Human Umbilical Vein Endothelial Cells (HUVECs) and the human monocytic leukemia cell line THP-1/macrophages were obtained from the American Type Culture Collection (ATCC). The MKN74, MKN45, MFC and THP-1 cell lines were cultured in RPMI-1640 medium supplemented with 10% fetal bovine serum (FBS), whereas the CAFs and HUVECs cell lines were cultured in Dulbecco’s modified Eagle medium (DMEM) supplemented with 10% FBS. All cell lines were incubated at 37 °C in a 5% CO_2_ environment. Furthermore, Short tandem repeat (STR) profiling was utilized to authenticate the cell lines, and effective detection of Mycoplasma contamination in the cells was accomplished.

## Neutrophil isolation

The protocol for separating tumor-infiltrated neutrophils involved sectioning Human gastric cancer tissues and C57BL/6 mice xenograft tumor into 0.5 cm × 0.5 cm blocks, which were then placed in a tube containing 10 mL DMEM medium, 100 μL collagenase I, and 100 μL DNase I. The resulting mixture was thoroughly mixed, and the tissue blocks underwent centrifugation, grinding, and filtration using a 200-mesh steel mesh. Subsequently, the resulting single-cell suspension was collected in a 50-mL centrifuge tube and centrifuged at 1700 rpm for 10 minutes. The cells were subsequently resuspended in phosphate-buffered saline (PBS) and filtered once more.

Human Neutrophils were isolated using the EasySep^TM^ Human Neutrophil Enrichment Kit, a method known to consistently yield a purity of over 95% neutrophils. Following the manufacturer's instructions, the single cell suspension was incubated with the EasySep Human Neutrophil Enrichment cocktail for 10 minutes at room temperature (25 ± 22°C). After this incubation period, EasySep nanoparticles were added to the mixture and incubated for an additional 10 minutes. Subsequently, the suspension was thoroughly mixed and adjusted to a final volume of 2.5 mL. To measure neutrophil purity, we used two methods: Giemsa staining and flow cytometry. CD66b+CD16+ neutrophils were identified. The purity of the neutrophils after enrichment was over 90% (as shown in Figure S1A).

A MojoSort mouse neutrophil isolation kit (BioLegend) was used to isolate murine neutrophils as per the manufacturer’s instructions. To measure neutrophil purity, we used two methods: Giemsa staining and flow cytometry. CD45+Ly6G+ neutrophils were identified. The purity of the neutrophils after enrichment was over 95% (as shown in Figure S1B).

## CD3+ T cells Isolation

CD3+T cells were isolated by flow cytometry sorting (Sony, MA900, Tokyo, Japan). Peripheral blood was collected from the patients and healthy individuals. 50 μl of peripheral blood were stained with anti-CD3 5μL and sorted by flow cytometry.The purity of the isolated CD3+T cells was over 95% (as shown in Figure S1C).

## Lentiviral vector and Plasmid construction and transfection

All the plasmids and lentiviruses were designed and constructed by GeneChem. Detailed information on the construction of various plasmids and production of the lentivirus and Plasmid are described in the Table S4-9. The transfection protocol was carried out per the manufacturer’s guide. The accuracy of transduction, knockdown, and overexpression, were assessed via western blotting (WB) (Figure S2). The protocol with the highest transfection effectiveness was selected.

## Establishment of co-culture units

In this investigation, non-contact co-culture units to simulate the tumor microenvironment. Different co-culture procedures were performed depending on the experimental requirements. We used schematic diagrams to explain different co-culture experiments.

## Xenograft tumor model

Four-week-old male BALB/c nude mice (weigh 18-22g) bought from the Beijing Weitong Lihua Experimental Animal Technology Co., Ltd. (Certificate No. SYXK2019-0010). were used in this study (six randomly allocated for each group). All mice were housed in a specific pathogen-free environment. A total of 1 × 10^5^ Neutrophils transfected with or without NC/knockdown/overexpression IFIT1 were injected subcutaneously with 1 × 10^6^ luciferase-labeled MKN45 (MKN45: Neutrophils = 10:1). Different groups of neutrophils were injected into the tumor every other day, beginning on day 7 after inoculation. Following 5 weeks, mice were intravenously injected with D-luciferin (100 mg/kg) through the caudal vein and imaged 10 minutes post-injection using the IVIS Illumina System (Caliper Life Sciences, Waltham, MA, USA). Subsequently, the mice were anesthetized in accordance with the American Veterinary Medical Association (AVMA) Guidelines for Humane Animal Euthanasia, employing CO_2_. Serum samples were collected, and tumor specimens were obtained for volume analysis using the formula V = 1/2ab^2^, as well as for generating growth curves. The in-vivo investigations were authorized by the ethical board of the Animal Ethics Committee of Jiangsu Province Hospital of Chinese Medicine (2022DW-44-02).

## Sample collection

64 patients with GC (30 PCC and 34 NPCC) were enrolled from the Jiangsu Province Hospital of Chinese Medicine. None of the patients had received any treatment, including chemotherapy, radiotherapy, and antitumor treatment with biological products, before surgery. After being informed, patients read and signed their consent for collection of samples during surgery. Tumor staging and grading for each patient were performed using the American Joint Committee on Cancer (AJCC) TNM staging system. All samples were confirmed by the Department of Pathology in our hospital. Detailed patient information is outlined in Table S10.

# Supplementary Figures

## Figure S1


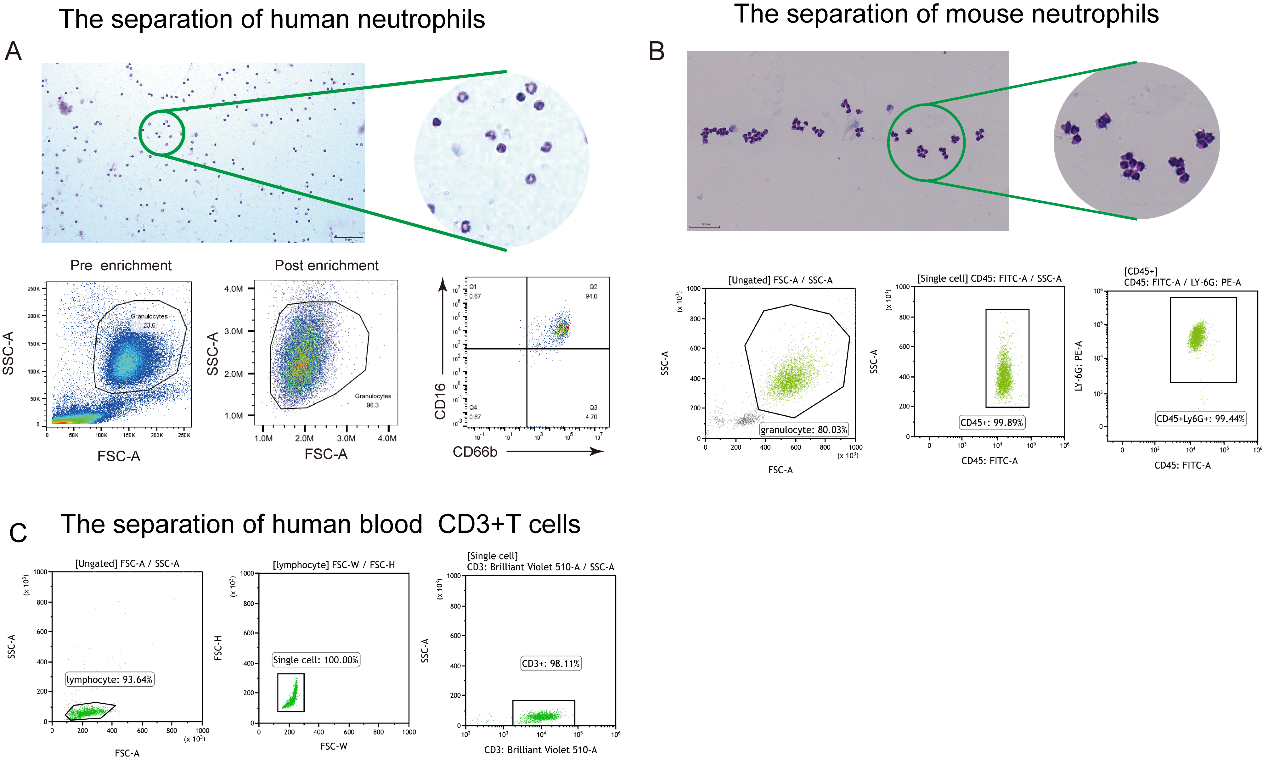


Figure S1. Isolation, purification and identification of immune cells.

(A-B) The human and mouse neutrophil purity was qualified by Wright-Giemsa stain method. Flow cytometry analysis of the purity of isolated neutrophils (human: CD16+CD66b+; mouse CD45+Ly6G+). Purity was greater than 90%.

(C) The purity of CD3+ T cells was more than 95%.

## Figure S2


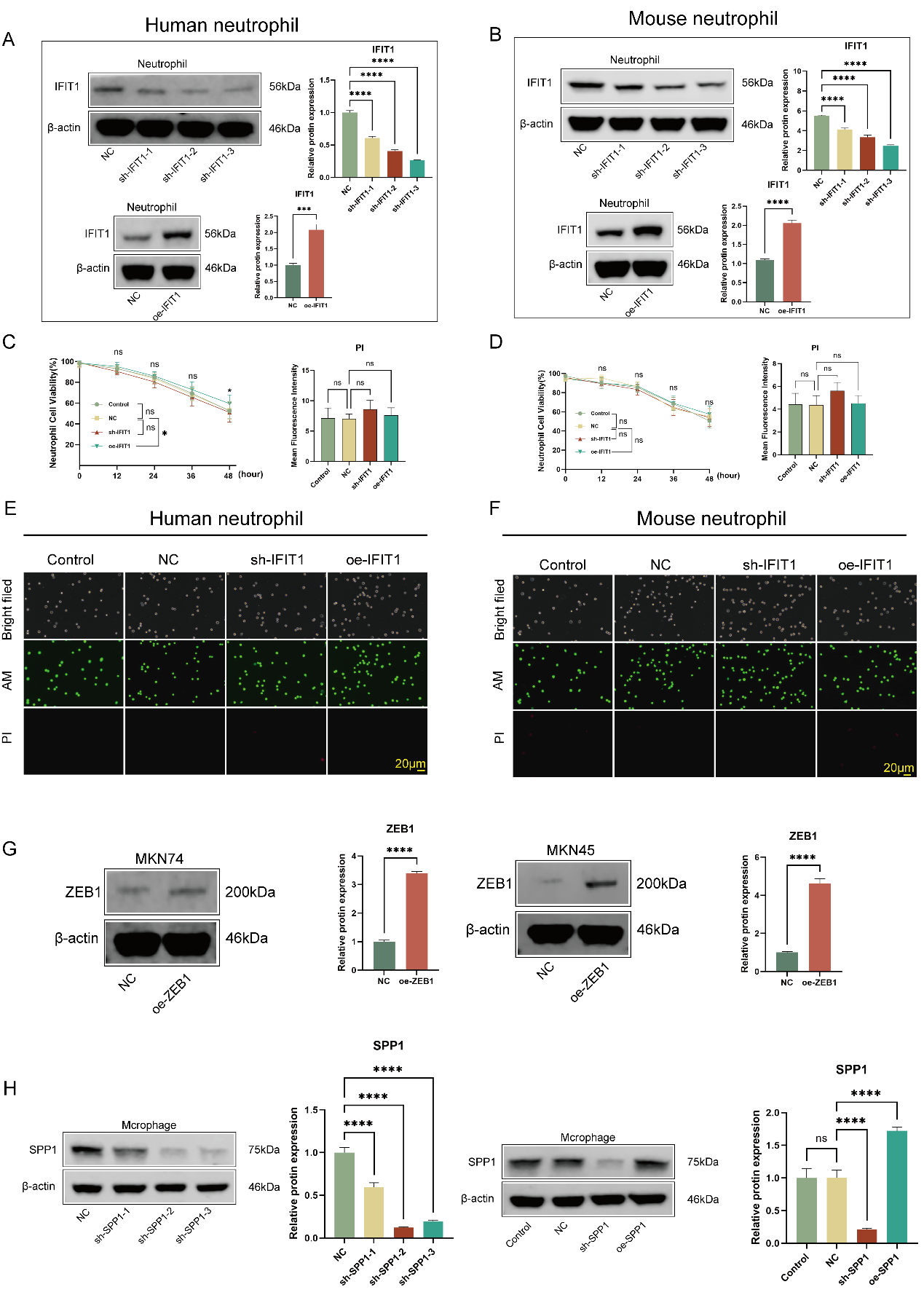


Figure S2. Transfection verification.

(A-B) Transfection efficiency of IFIT1 was verified by WB assay.

(C-F) IFIT1 knockdown in neutrophil has no effect on cell growth.

(G-H) Transfection efficiency of ZEB1and SPP1 was verified by WB assay.

## Figure S3


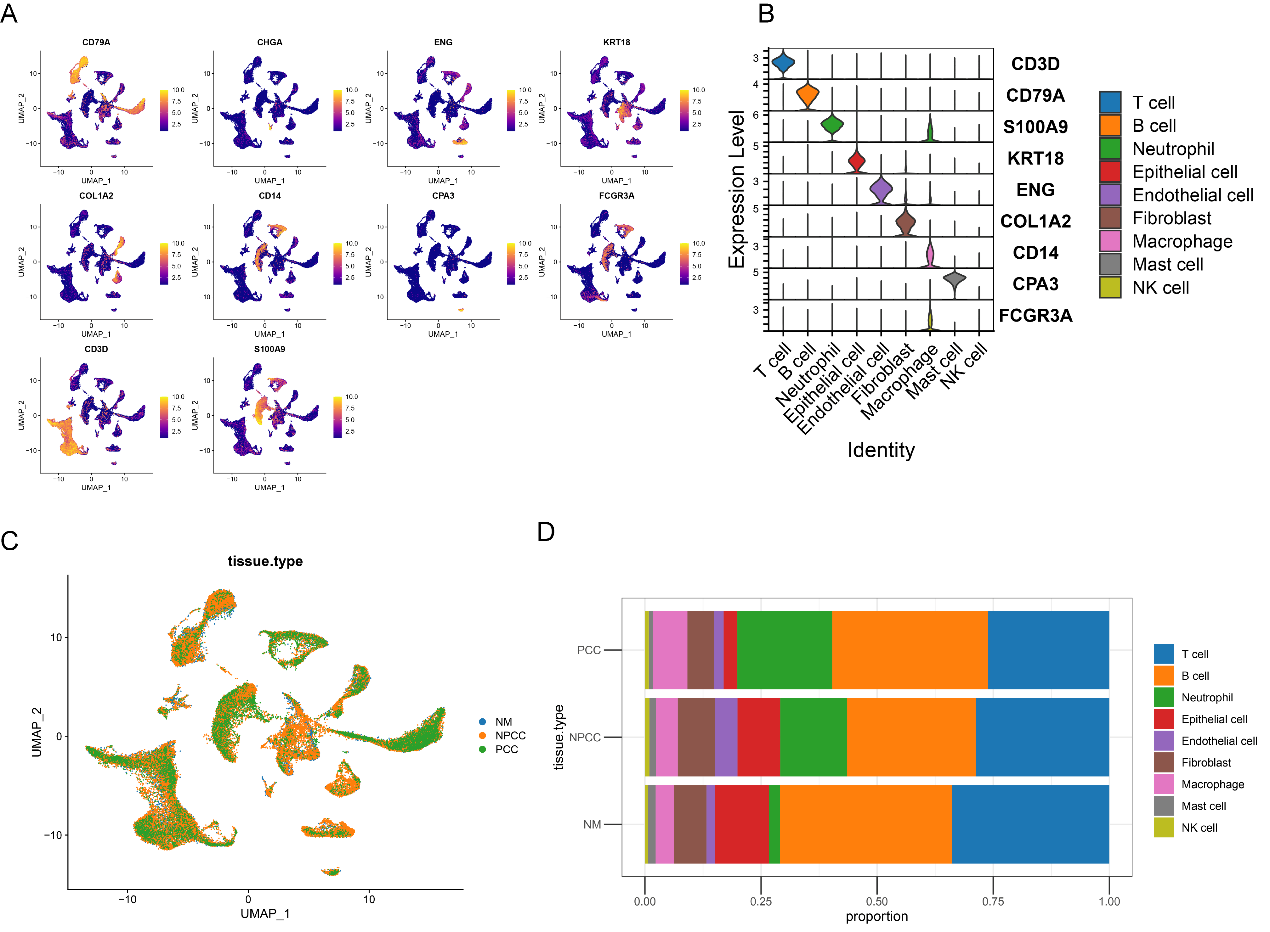


Figure S3. Representative single-cell transcriptome landscape of gastric cancer (GC). See also Figure 1A.

(A) Uniform Manifold Approximation and Projection (UMAP) plots showing the expression distribution of marker genes in nine cell types.

(B) Violin plots showing the smoothed expression distribution of corresponding markers for different cell types.

(C) UMAP plots for the 64,454 retained cells showing tissue origin. poorly cohesive carcinoma (PCC), non-poorly cohesive carcinoma (NPCC), and Normal mucosa (NM)

(D) The proportion of each cell type in 3 tissue types, including PCC, non-poorly NPCC, and NM.

## Figure S4


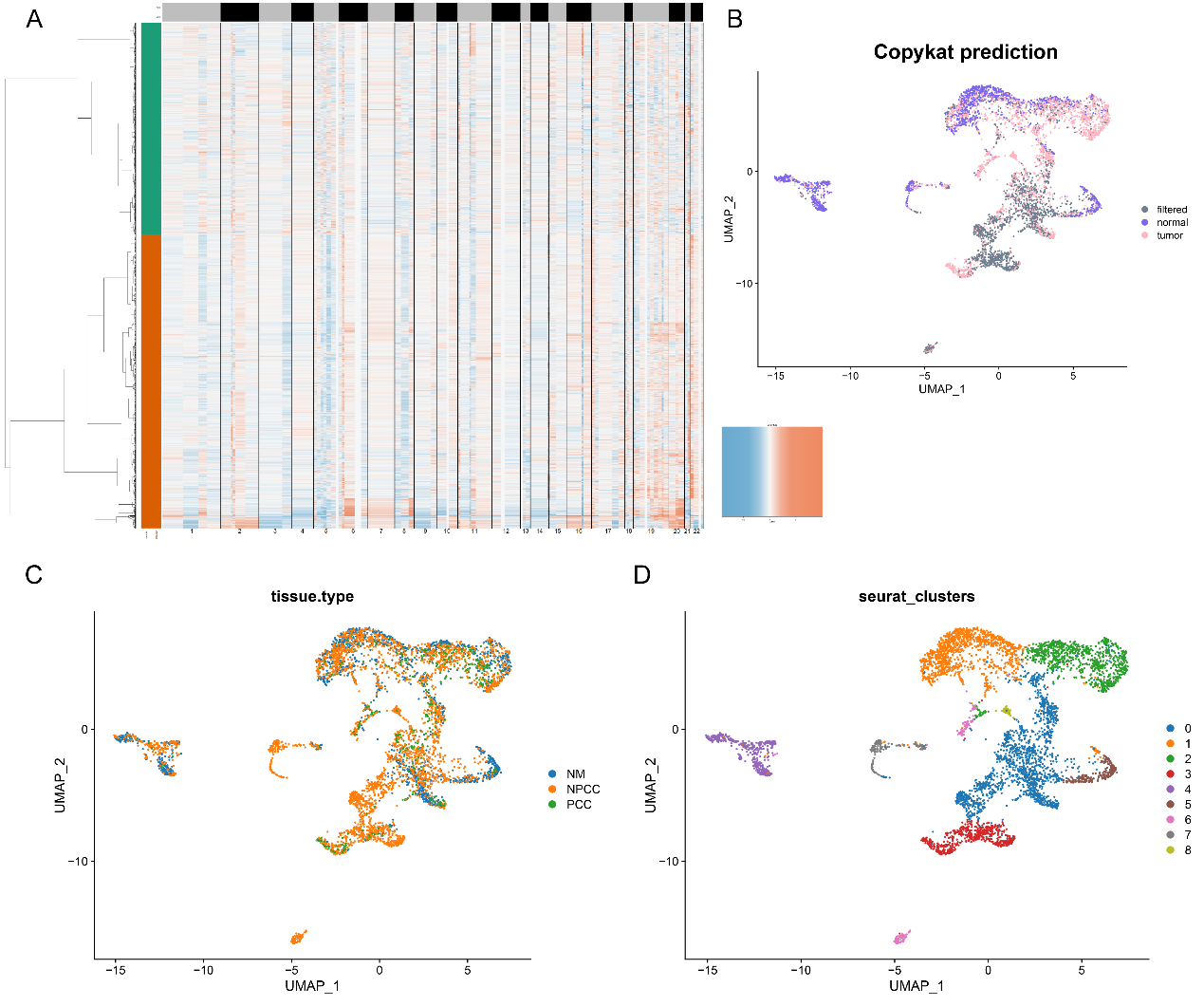


Figure S4. Malignant and non-malignant cell annotations. See also Figure 1E.

(A) Heatmap showing chromosomal landscape of large-scale copy number variations (CNVs) inferred with Copykat from single cell RNA data.

(B) UMAP plot showing different Copykat predictions of epithelial cells.
(C-D) UMAP plots for 5,210 Epithelial cells showing tissue origin (C) and seurat clusters (D).

## Figure S5


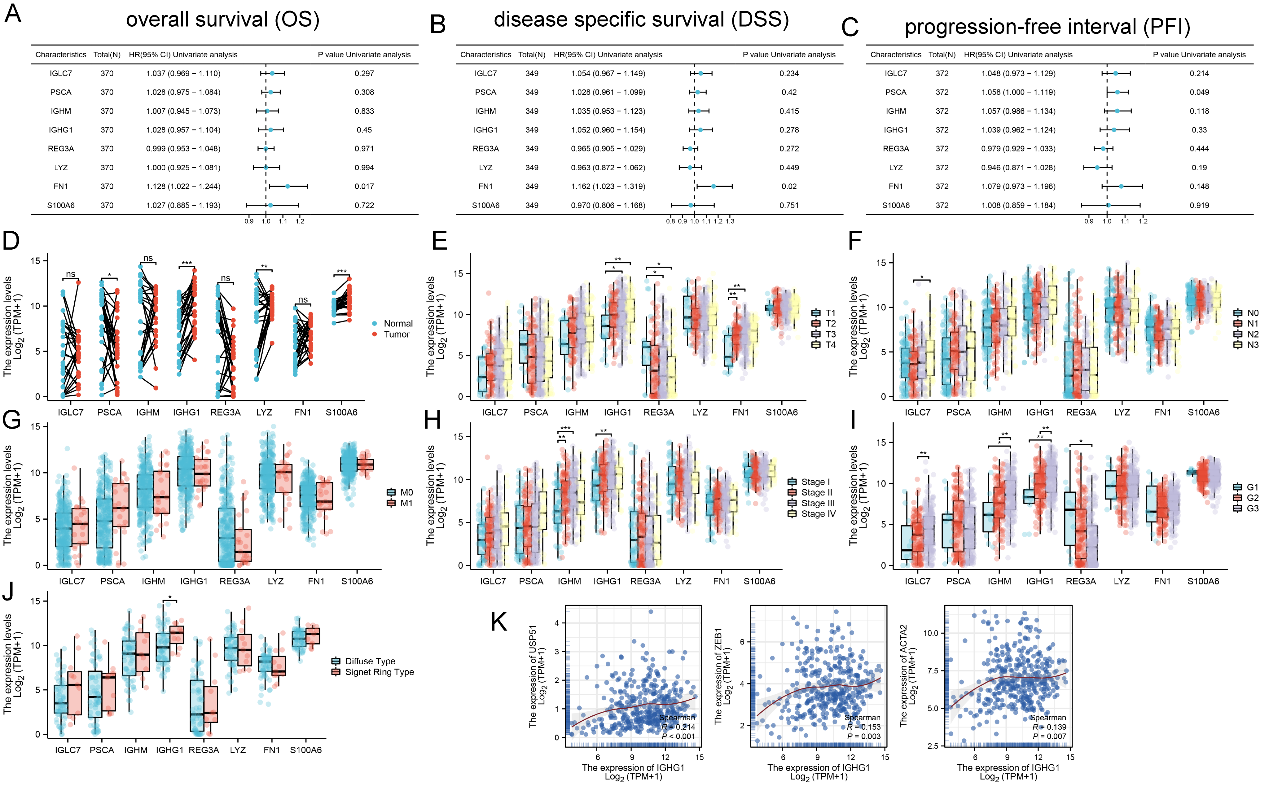
Figure S5. Bulk analysis of differentially expressed genes (DEGs) between PCC and NPCC malignant cells. See also Figure 1H.

(A-C) Forest maps showing the results of Cox regression analysis on the average overall survival (A, OS), disease specific survival (B, DSS), and progression free interval (C, PFI) rate of 8 differential expression genes (DEGs) in the The Cancer Genome Atlas (TCGA-STAD) cohort.

(D) Box plots showing the mRNA expression of 8 DEGs in paired normal and tumor tissues (n = 54). Ns: not significant, *P < 0.05, **P < 0.01, ***P < 0.001. T test was conducted.

(E-J) Analysis of 8 DEGs mRNA expression in different subtypes of STAD tissues from TCGA database, including T stages (E), N stages (F), M stages (G), pathological stages H), tumor grades (I), and histological types (J). For two-group comparisons, the Wilcoxon rank sum test was used; for multiple-group comparisons, the Kruskal-Wallis test was used. *P < 0.05, ** P < 0.01, *** P < 0.001.

(K) Spearman correlation between *IGHG1* expression and expression levels of USP51-ZEB1-ACTA2 signaling as analyzed in TCGA-STAD.

## Figure S6


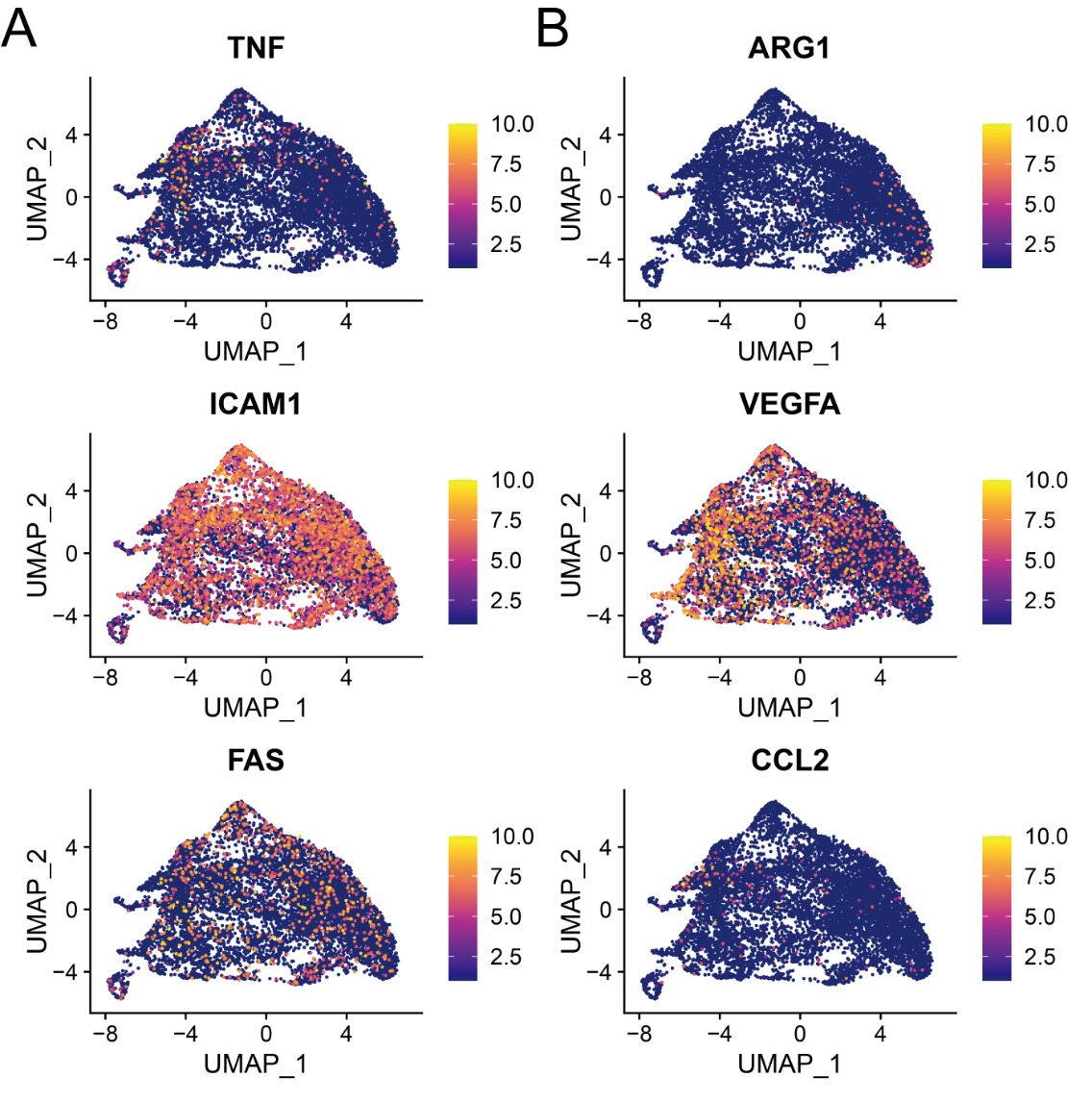


Figure S6. Expression of N1 and N2 markers in neutrophils. See also Figure 2E.

(A) UMAP plots of N1 markers in neutrophils, including Tumor Necrosis Factor (TNF), Intercellular Adhesion Molecule 1 (ICAM1), and Fas Cell Surface Death Receptor (FAS).

(B) UMAP plots of N2 markers in neutrophils, including Arginase 1, (ARG1), Vascular Endothelial Growth Factor (VEGF), and C-C Motif Chemokine Ligand 2 (CCL2).

## Figure S7


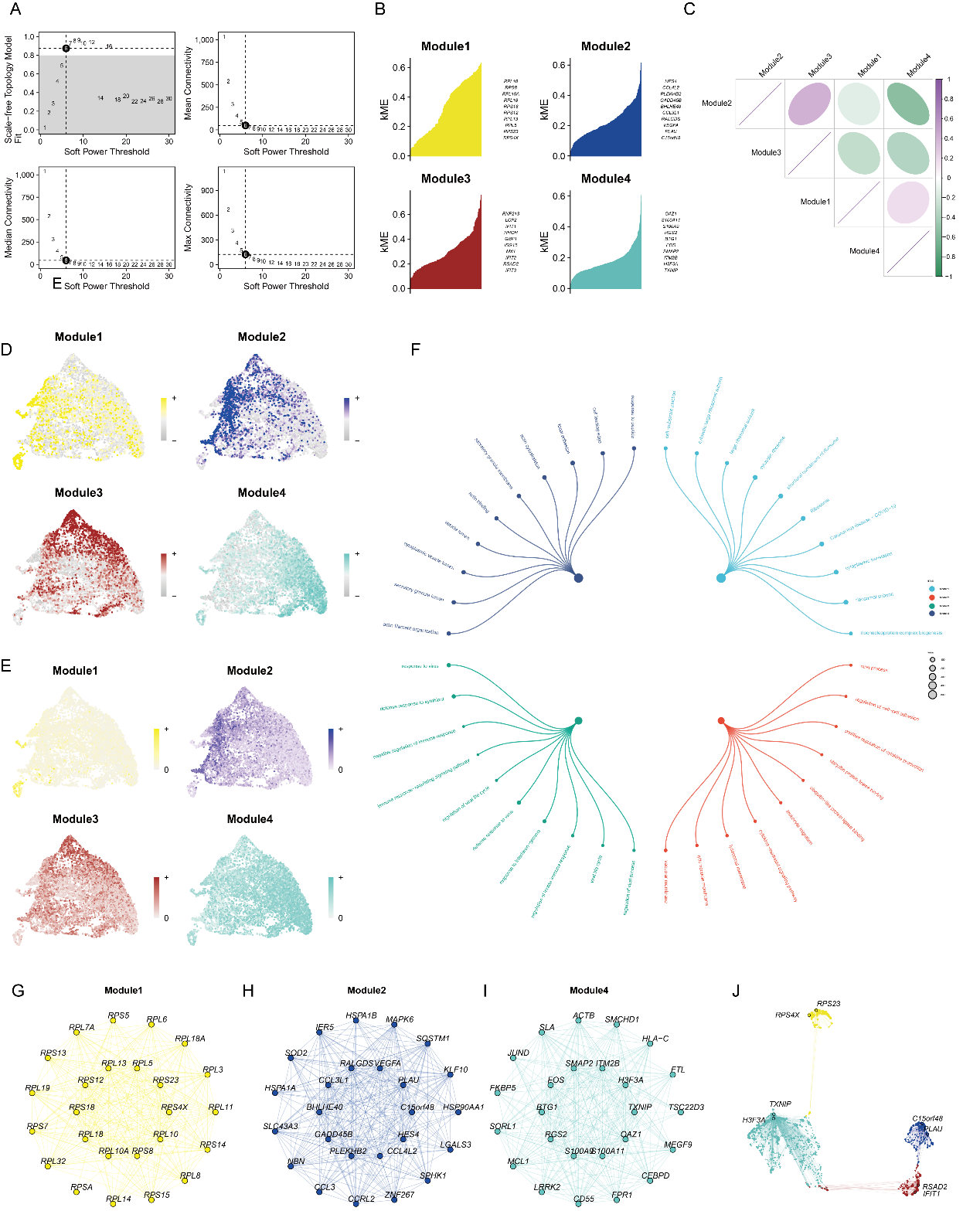


Figure S7. high-dimensional Weighted Gene Co-expression Network Analysis (hdWGCNA) defines a *CD274*-related neutrophil gene module, which represents PCC-specific neutrophil infiltration. See also Figure 2F.

(A) Soft power = 6 was selected to construct the scale-free network.

(B) Highly variable genes were clustered into 4 modules through hdWGCNA.

(C) Correlation analysis between four identified modules by hdWGCNA.

(D-E) Distribution of module scores (D) and hub-gene expressions (R) in neutrophils. There are total four different gene modules for neutrophils.

(F) Gene Ontology (GO) and Kyoto Encyclopedia of Genes and Genomes (KEGG) pathway enrichment analysis of genes in the four modules. The larger the bubbles, the more significant the enriched terms.

(G-I) The specific network structures of Module 1 (G), 2 (H), and 4 (I).

(J) Network diagram showing the interactions between the four modules. Important gene nodes are labeled.

## Figure S8


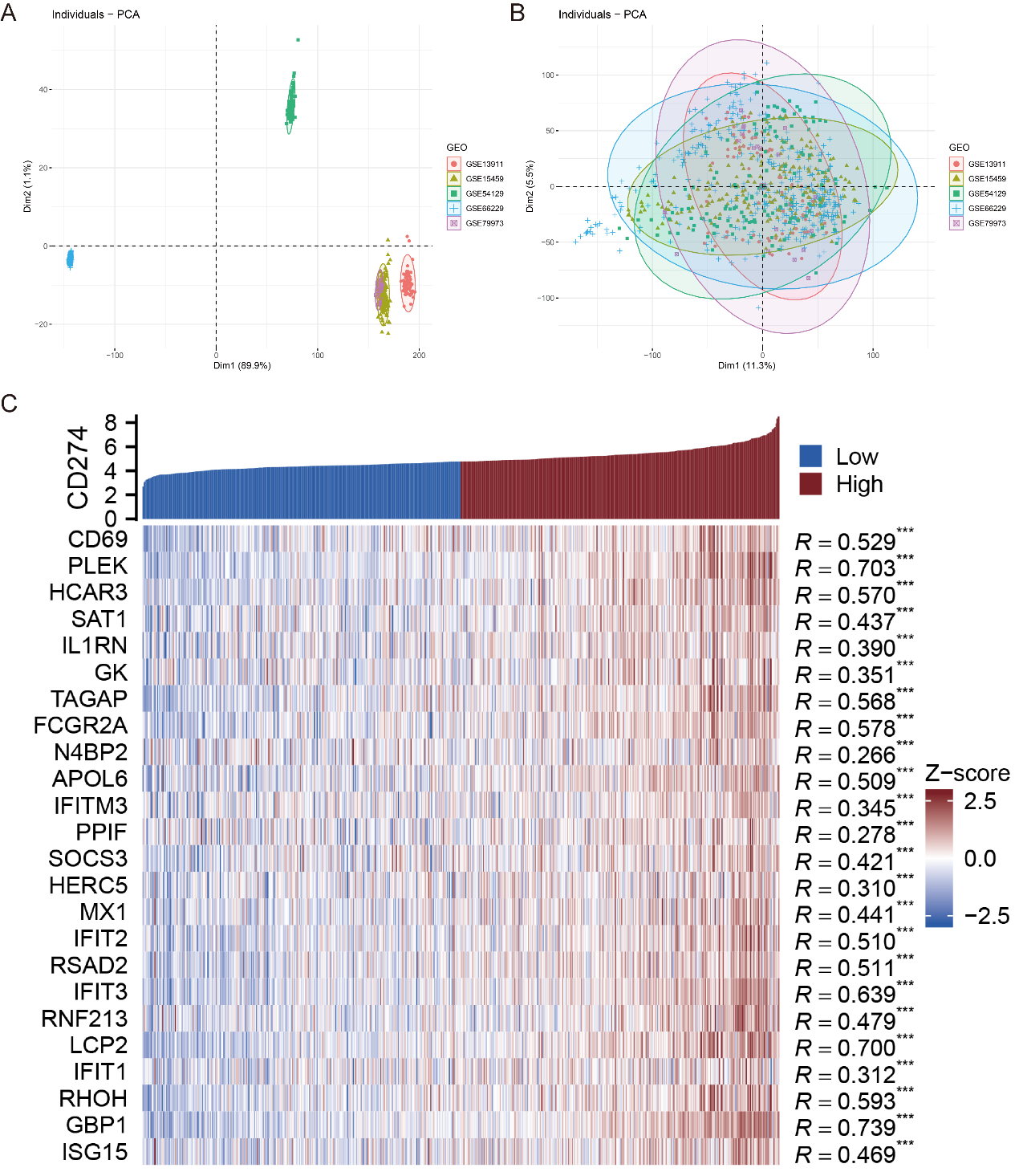


Figure S8. The merged Gene-Expression Omnibus (mGEO) data was generated to be used as an external validation. See also Figure 2J.

(A-B) Principal component analysis (PCA) shows the gene expression distribution in 5 GC cohort samples (GSE66229, GSE15459, GSE13911, GSE79973, GSE54129) before (A) and after (B) batch effect correction.

(C) Heatmap showing the correlations between *CD274* and Module-3 members in mGEO. Red represents positive values and blue represents negative, normalizing gene expression to a Z-score.

## Figure S9


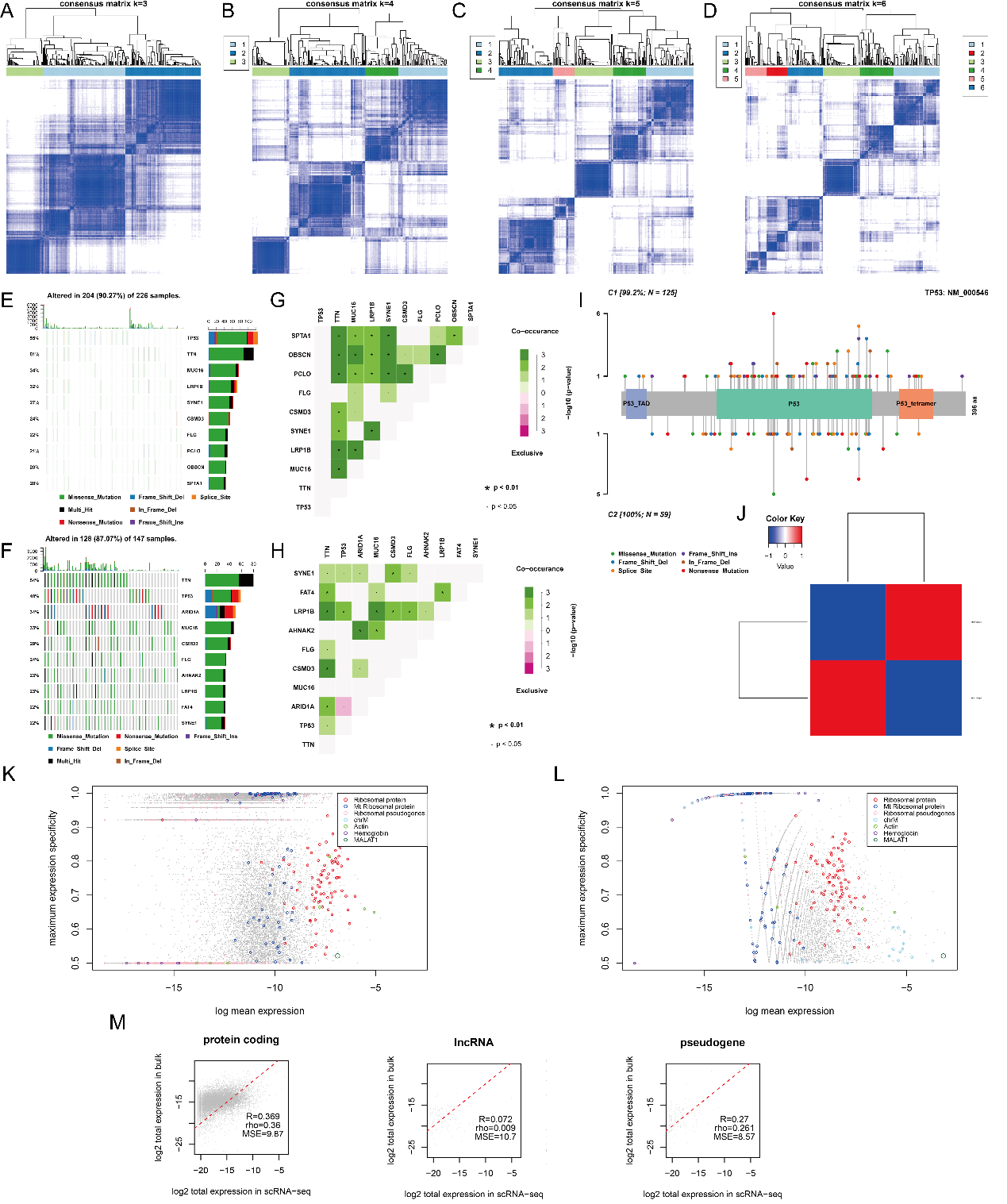


Figure S9. Unsupervised learning to identify two classification keywords by module 3. See also Figure 3.

(A-D) The consensus clustering matrix for k = 3 (A), 4 (B), 5 (C), and 6 (D).

(E-F) Oncoplot displaying mutation profile of a union set of the top 10 genes with highest mutation frequency in cluster 1 (E) and 2 (F), respectively.

(G-H) Graphs showing mutually exclusive and co-occurrence of mutational events in cluster 1 (G) and 2 (H), respectively.

(I) The mutation site profile of the *TP53* gene is shown in cluster 1 (above) and 2 (below), respectively.

(J) Heatmap showing distinct gene expression profiles of IFIT1+ TANs and IFIT1- TANs.

(K-L) Filtration of outlier genes for single-cell data matrix (K) and bulk data matrix (L).

(M) Scatter plots showing the consistency of different types of gene expression between bulk data and single cell RNA data.

## Figure S10


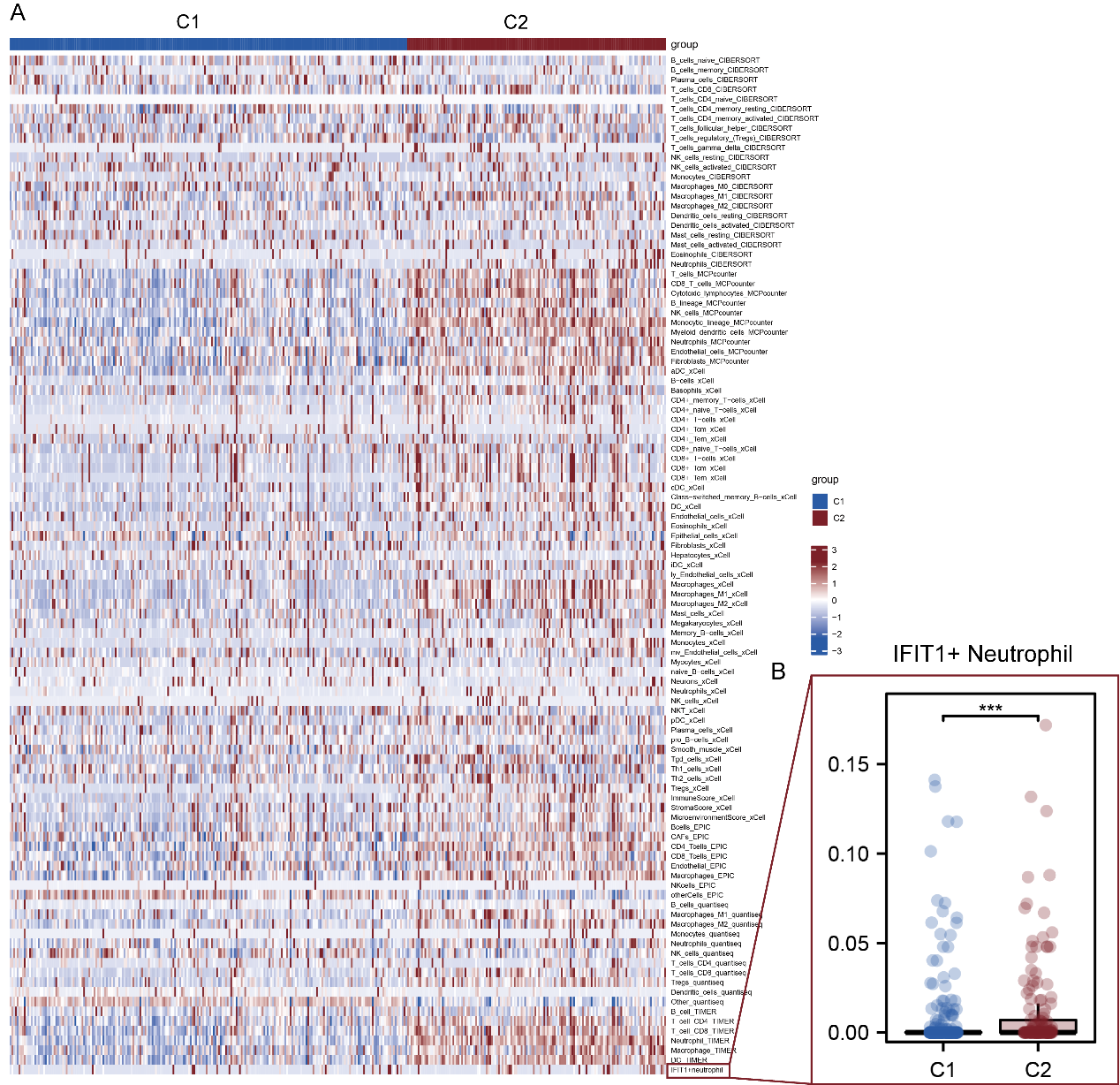


Figure S10. The variations in levels of immune cell infiltration estimated by six algorithms between the two levels of module 3. See also Figure 3K.

(A) Heatmap demonstrating differences in the level of infiltration of all immune cells. Blue represents low levels and red represents high levels.

(B) Heatmap demonstrating differences in IFIT1+ neutrophils.

## Figure S11


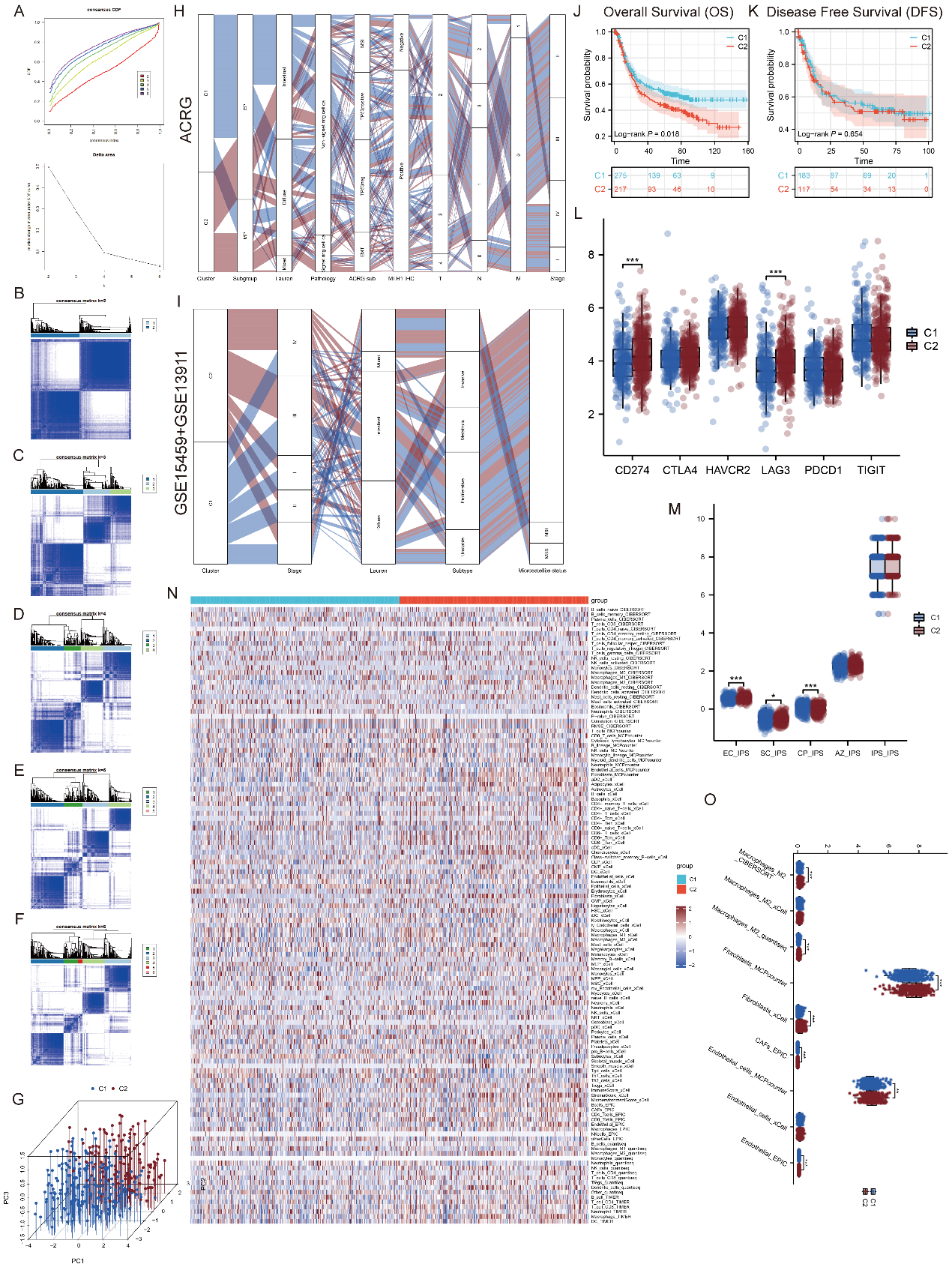


Figure S11. Unsupervised learning to identify two classification keywords by module 3 in mGEO. See also Figure 3.

(A-F) The consensus clustering matrix for k = 2 was determined by CDF for k = 2-6.

(G) PCA showed that the expression of the 25 module-3 molecules defined distinct phenotypes.

(H-I) Sankey diagram showing the correlation between GC classifications and clinical parameters, ACRG subtype, and Singapore subtype.

(J-K) The Kaplan-Meier curve shows significant Overall Survival (OS, J) and Disease-Free Survival (DFS, K) rate differences between the two kinds of phenotypes in mGEO.

(L-M) Box plots showing the expression of the immune checkpoint genes and Immune cell Proportion (IPS) Score in different phenotypes (mGEO, n = 651). ***P < 0.001, Wilcoxon rank sum test.

(N) Heatmap showing variations in levels of immune cell infiltration estimated by six algorithms between the two levels of module 3. Blue represents low levels and red represents high levels.

(O) Box plots highlighting the level of pro-tumor cell infiltration we focus on. **P < 0.01, ***P < 0.001, Wilcoxon rank sum test.

## Figure S12


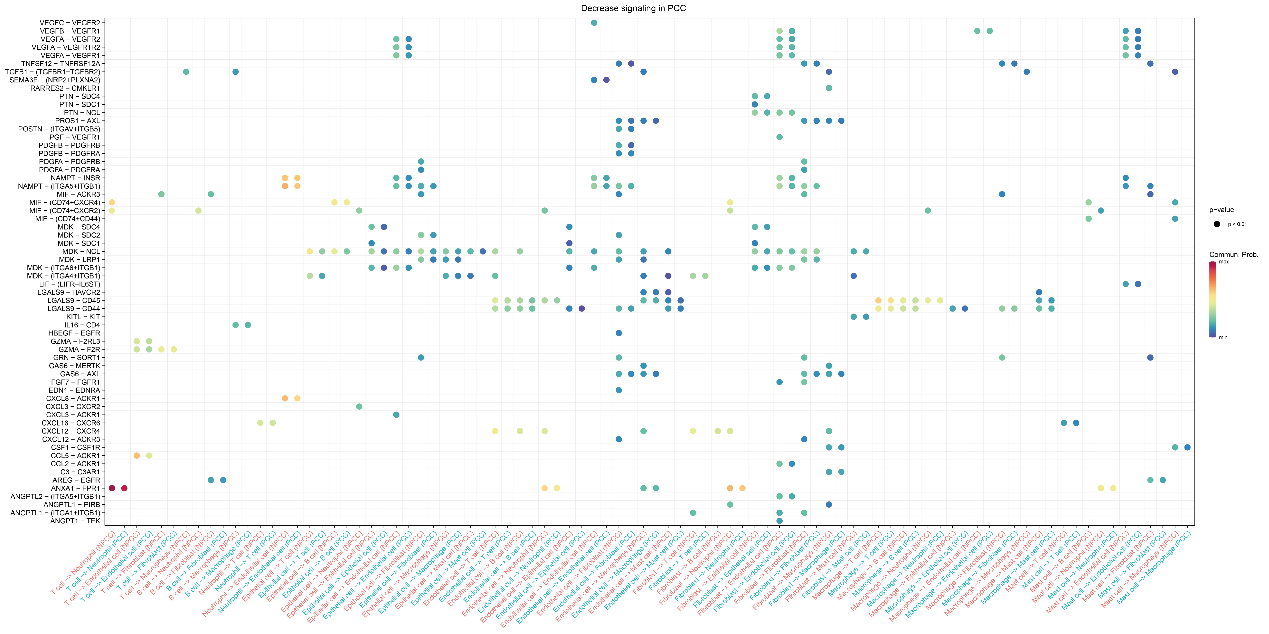


Figure S12. Bubble diagram showing that fibroblasts, macrophages, endothelial cells and epithelial cells may communicate with neutrophils frequently through multiple signals in PCC samples. See also Figure 4.

## Figure S13


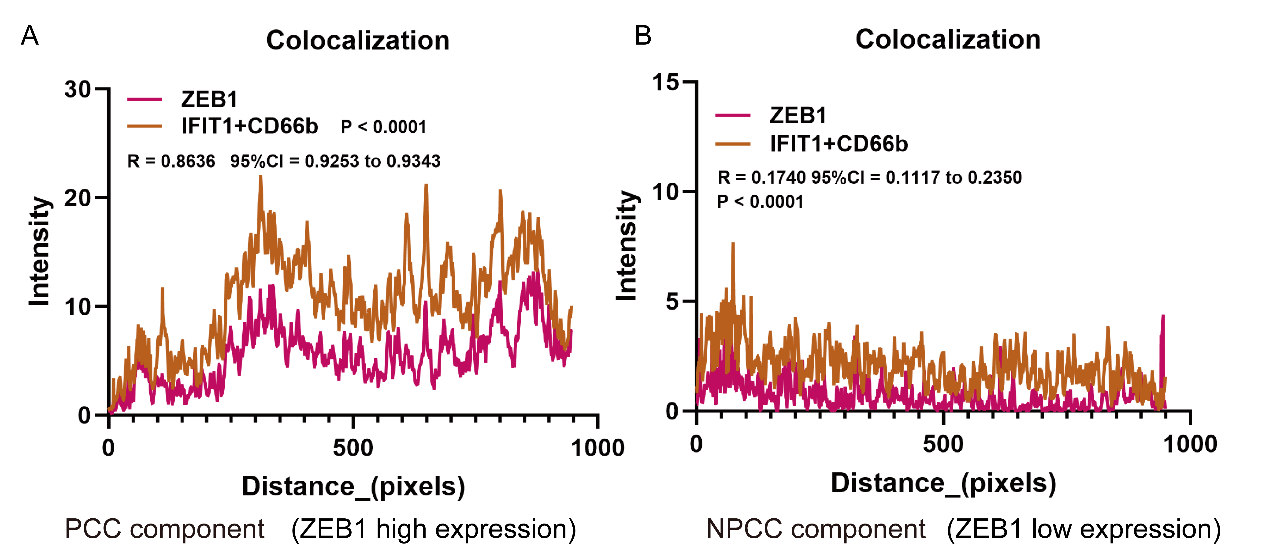


Figure S13. The degree of co-localization of IFIT1 and ZEB1 in the PCC component (ZEB1 high expression) and the NPCC component (ZEB1 low expression). See also Figure 5H.

(A-B) Co-localization was quantified using the Spearman correlation coefficient in PCC component (A) and NPCC component (B).

## Figure S14


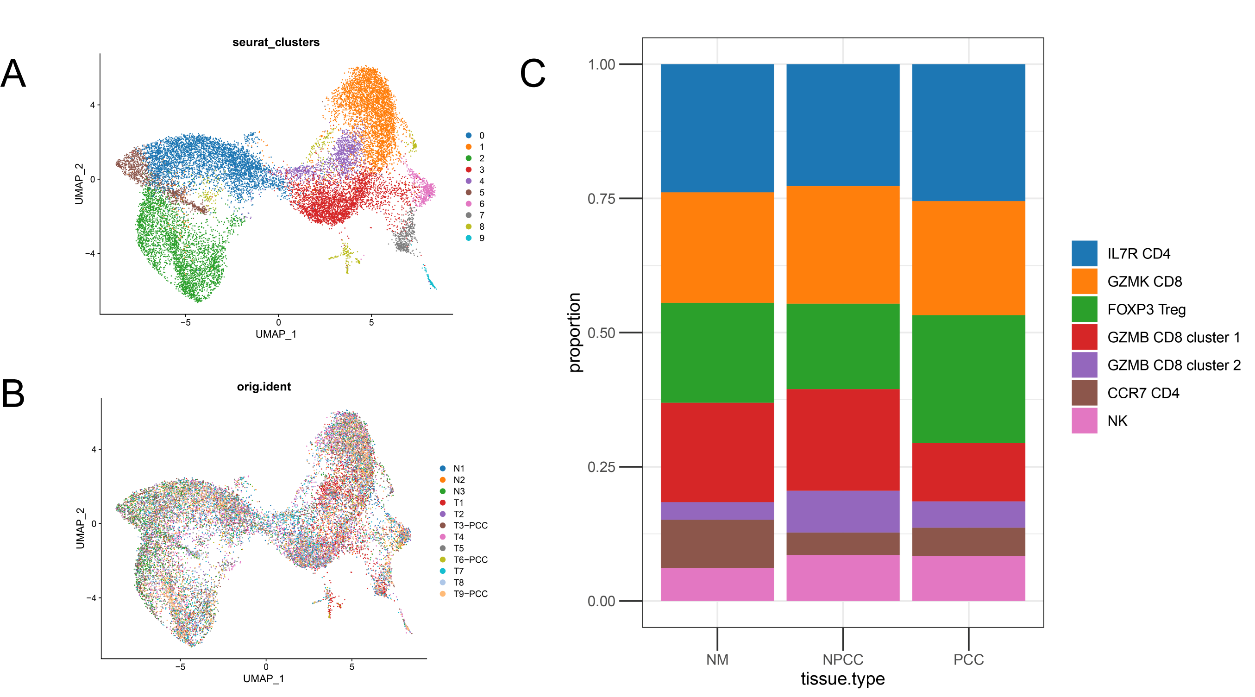


Figure S14. Representative single-cell transcriptional profiles of T cells and NK cells.

(A) UMAP plot showing different clusters of T and NK cells. See also Figure 6A.

(B) UMAP plot showing different sample origins of T and NK cells. See also Figure 6A.

(C) The proportion of each NK/T cell type in 3 tissue types, including PCC, NPCC, and NM. See also Figure 6D.

## Figure S15


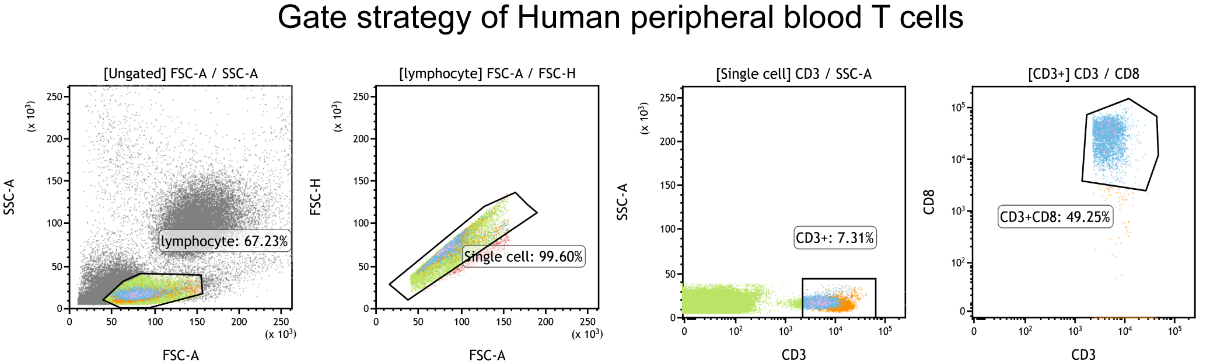


Figure S15. Flow cytometry gating strategy for detection of T cell activity and exhaustion. We identified Non-debris Cells by FSC (forward scatter) and SSC (side scatter) characteristics as indicated (left 1). Single cells were then gated based on FSC-A and FSC-H (left 2). The final sort for the GC fractions was of single cells from the CD3-compartment (right 2) and CD3/CD8 (right 1). Percentages reflect the fraction of the (previous) parent population. See also Figure 6G.

## Figure S16


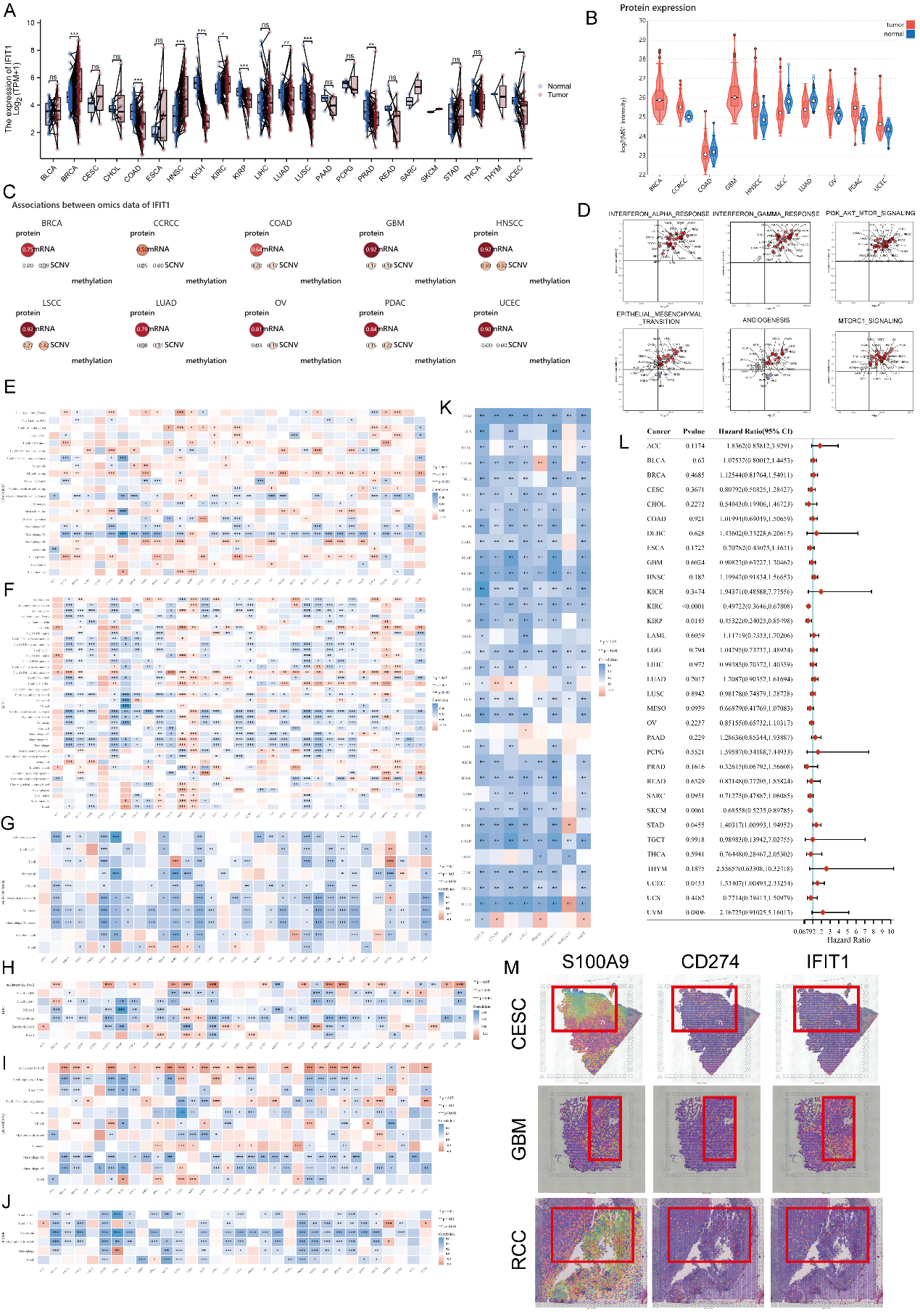


Figure S16. Role of IFIT1 in pan-cancer. See also Figure 9.

(A) The mRNA expression of *IFIT1* between paired tumor and normal control tissues was evaluated in TCGA database.

(B) The protein expression of *IFIT1* between paired tumor and normal control tissues was evaluated by LinkedOmicsKB database.

(C) Associations between transcriptome data and proteomics/Somatic copy number variation (SCNV)/methylation data of IFIT1 were assessed by LinkedOmicsKB database.

(D) Association of *IFIT1* with several important biological signatures in human cancers from TCGA database.

(E-J) Spearman correlation of *IFIT1* with the infiltration level of immune cells in human cancers from TCGA database. The CIBERSORT (E), XCELL (F), MCPCOUNTER (G), EPIC (H), QUANTISEQ (I), and TIMER (J) algorithms were applied. Blue represents positive correlation and red represents negative correlation. *P < 0.05, **P < 0.01, ***P < 0.001.

(K) Spearman correlation of *IFIT1* with the level of immune checkpoint genes. Blue represents positive correlation and red represents negative correlation. *P < 0.05, **P < 0.01, ***P < 0.001.

(L) Univariate Cox regression analysis estimating prognostic value (OS) of *IFIT1* in pan-cancer database (TCGA). The length of horizontal line represents the 95% confidence interval (CI) for each cancer type. The vertical blue line represents HR = 1. HR > 1.0 indicates the overexpression of *IFIT1* is detrimental to OS. P value is displayed.

(M) Spatial transcription sections show the spatial expression of *S100A9*, *CD274*, and *IFIT1*. The dot color represents the expression level of the genes. Upper: Cervical Cancer (CESC), middle: Glioblastoma (GBM), bottom: Renal Cell Carcinoma (RCC).

## Figure S17


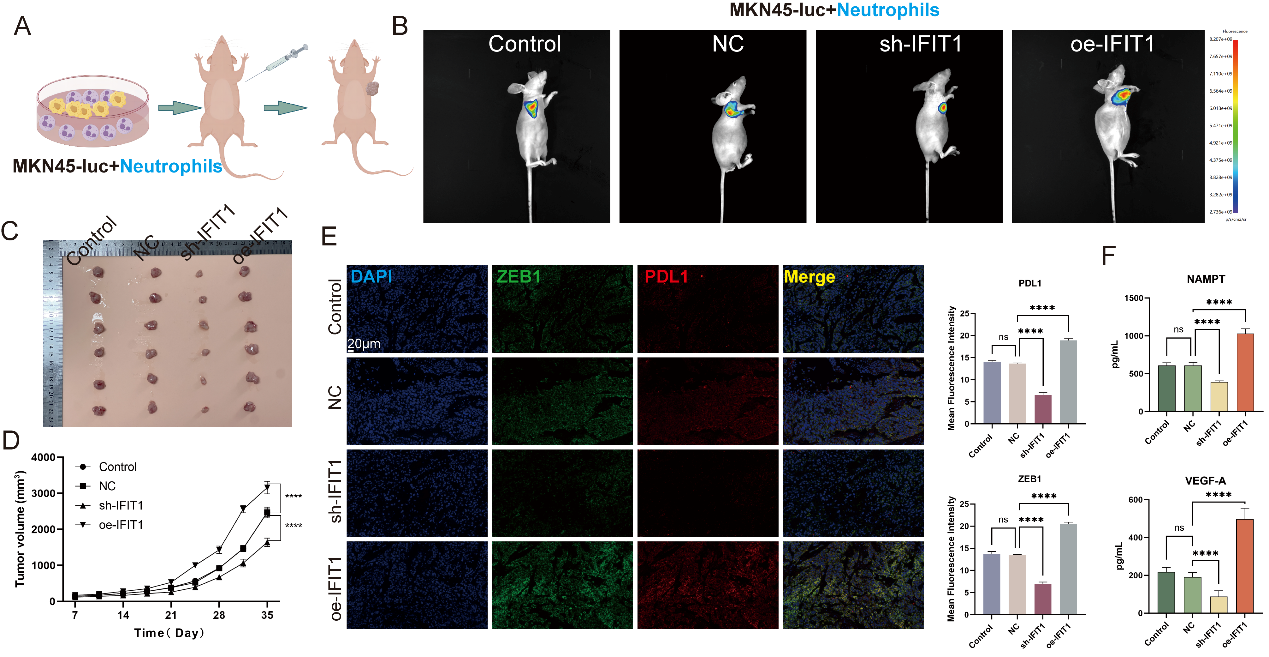


Figure S17. IFIT1+ neutrophils promote tumor growth in immunodeficient mice in vivo. See also Figure 9.

(A) Diagram of in vivo experimental design.

(B) The live imaging of mice (n = 6 per group).

(C-D) IFIT1 overexpressed neutrophils promoted tumor growth in the xenograft nude mouse model, whereas IFIT1 underexpression exerted the opposite effect. Control, TANs infected with a Flag-IFIT1 over-/under-expression vector (oe/sh IFIT1) mixed with MKN45-luc cells were injected into nude mice, and the tumor volume was measured at the indicated time points. ****P < 0.0001.

(E) mIF of ZEB1 and PDL1 expression in xenograft with the intervention of neutrophils (mixed with MKN45 cells) over-/under-expressing IFIT1. Scale bars, 20 μm. ns, not significant, ****P < 0.0001. vs. respective control by t-test.

(F) ELISA assay of NAMPT and VEGF-A in nude mouse blood serum. ns, not significant, ****P < 0.0001 vs. NC by t-test.

## Figure S18


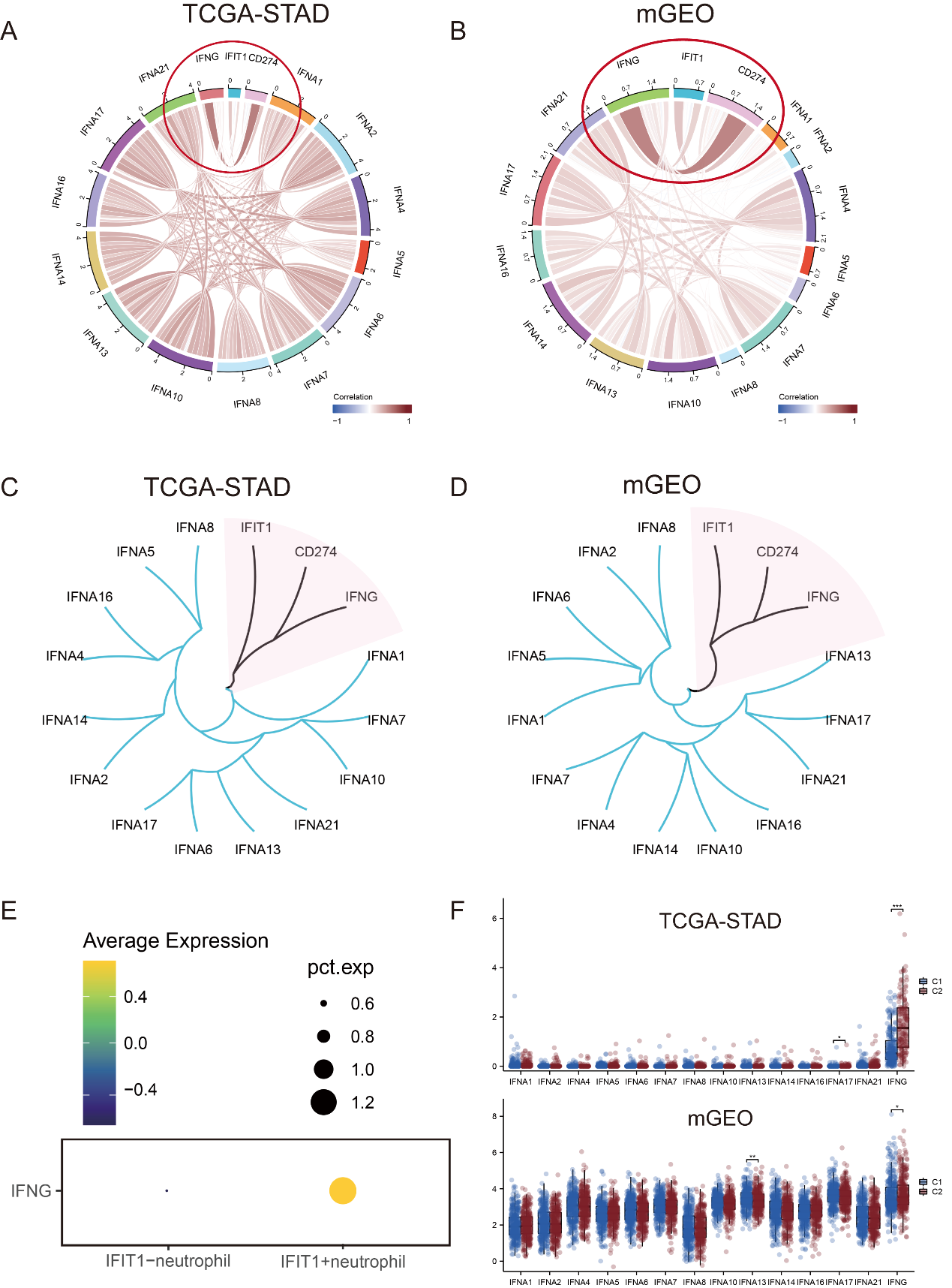


Figure S18. Transcriptomic analysis showing the correlation between IFIT1, PDL1, and IFNG. See also Figure 9A.

(A-B) Chord diagram showing the Spearman correlation among *IFIT1*, *CD274*, *IFNA*, and *IFNG* gene expression in TCGA-STAD (A) and mGEO (B). Red, positive correlation; blue, negative correlation. *IFIT1*, *CD274*, and *IFNG* were highlighted in red because of the high degree of correlation among the three.

(C-D) Cluster analysis of IFNA and IFNG gene expression in TCGA-STAD (C) and mGEO (D). a hierarchical clustering method with Manhattan distance was conducted. *IFIT1*, *CD274*, and *IFNG* were highlighted in pink because of similar expression patterns.

(E) Bubble plot showing the expression of *IFNG* in IFIT1+ TANs and IFIT1- TANs.

(L) Box plots showing the high *IFNG* expression in PCC samples (above, TCGA-STAD; below, mGEO). *P < 0.05, ***P < 0.001 vs. respective control by wilcoxon rank sum test.

(F) Box plots showing the high *IFNG* expression in PCC samples (above, TCGA-STAD; below, mGEO). *P < 0.05, ***P < 0.001 vs. respective control by wilcoxon rank sum test.

## Figure S19


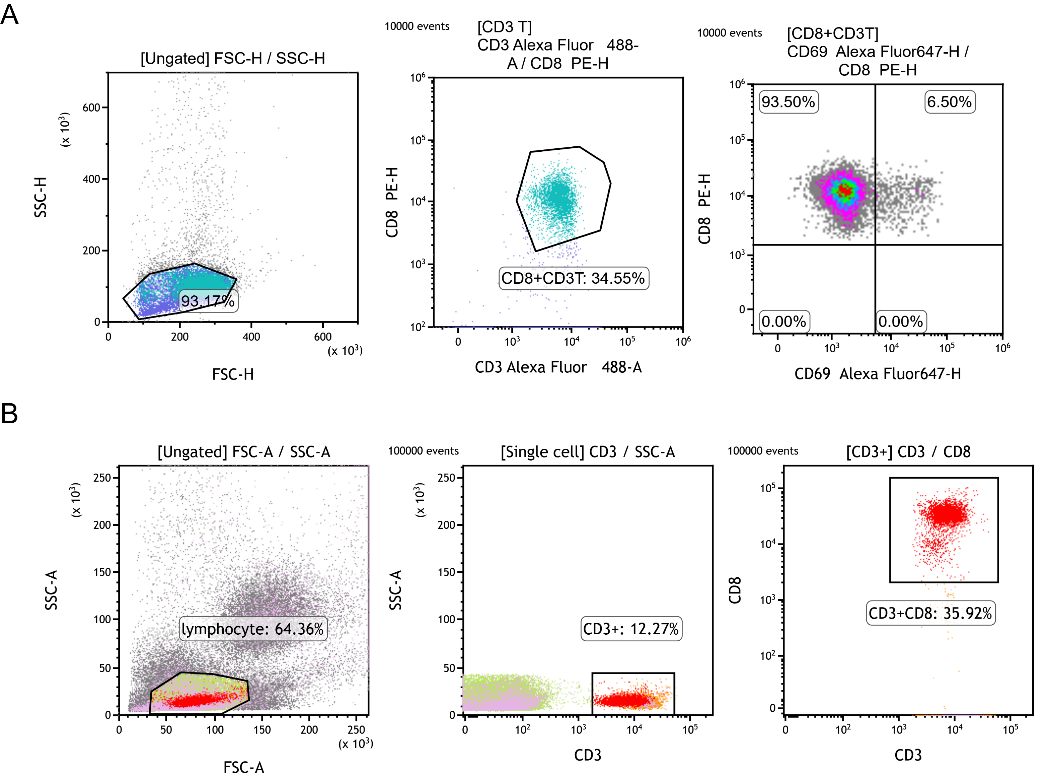


Figure S19. Flow cytometry gating strategy for detection of T cell activity and exhaustion.

(A) Flow cytometry gating strategy of in vitro experiments. See also Figure 9E.

(B) Flow cytometry gating strategy of in vivo experiments. See also Figures 9G and 9J.

## Figure S20


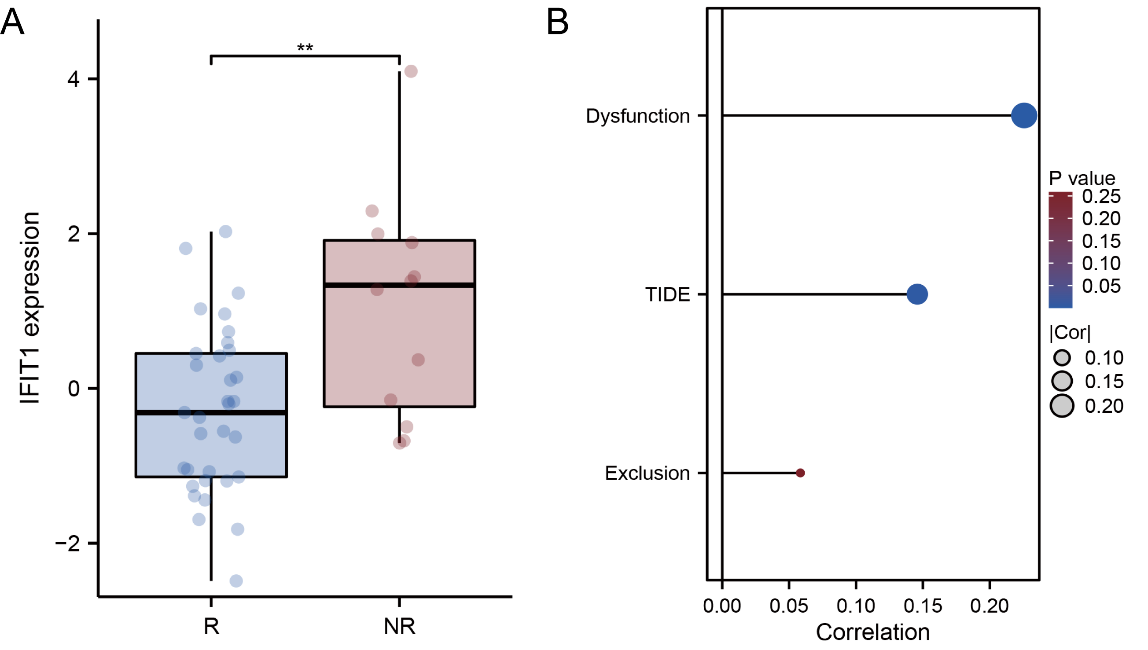


Figure S20. Bulk analysis showing the relationship between IFIT1 and immunotherapy response. See also Figure 9K.

(A) Box plot showing distinct *IFIT1* expression between responder and non-responder after anti-PD1 therapy in 45 GC patients from PRJEB25780 cohort.

(B) Bubble plot showing Spearman correlation between *IFIT1* expression with the tumor immune dysfunction and exclusion (TIDE) score in TCGA-STAD.

## Figure S21


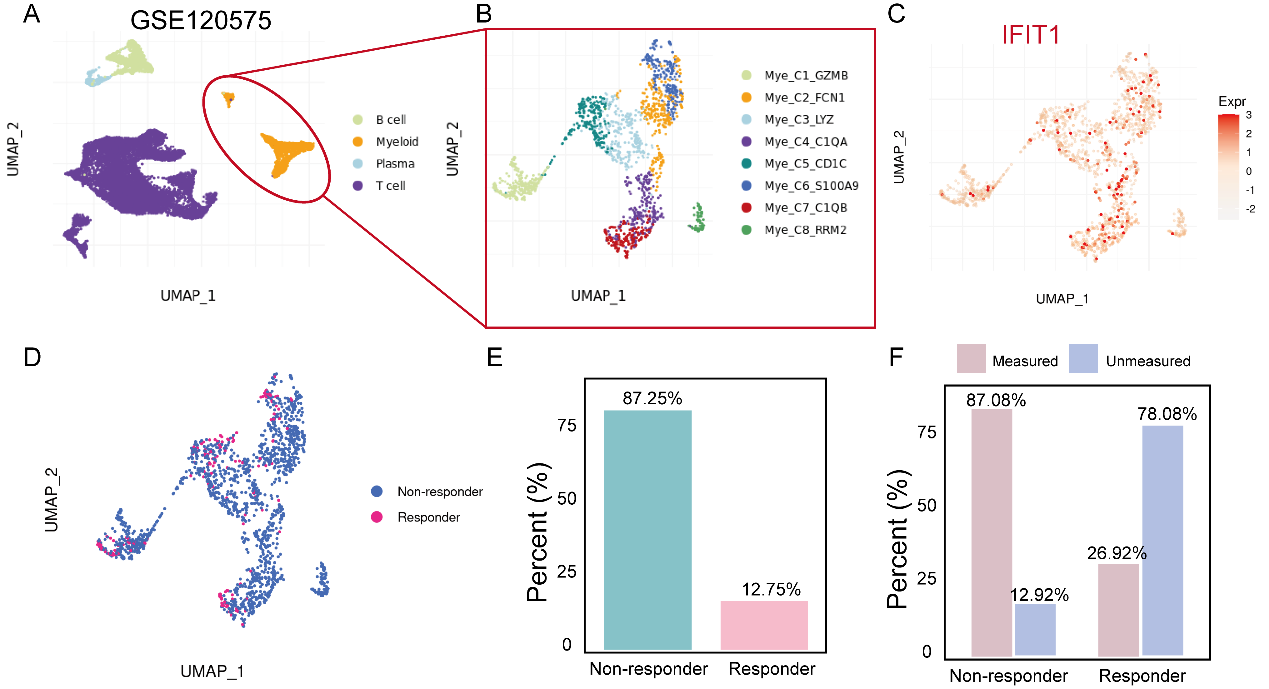


Figure S21. Single cell analysis showing the relationship between IFIT1 and immunotherapy response. See also Figure 9K.

(A-B) UMAP plot showing cell types identified by using single-cell RNA-seq dataset GSE120575.

(C) Distribution of *IFIT1* expression in different myeloid cell clusters.

(D-E) UMAP plot (E) and histogram (F) showing that S100A9+ myeloid cells were highly enriched in non-responder patients.

(F) Histogram showing the proportional expression values of *IFIT1* in S100A9+ myeloid cells of responder (26.92%) and non-responder (87.08%) patients.
